# The role of hypervalent iodine(iii) reagents in promoting alkoxylation of unactivated C(sp^3^)–H bonds catalyzed by palladium(ii) complexes†

**DOI:** 10.1039/d1sc01230d

**Published:** 2021-04-14

**Authors:** Payam Abdolalian, Samaneh K. Tizhoush, Kaveh Farshadfar, Alireza Ariafard

**Affiliations:** Department of Chemistry, Islamic Azad University Central Tehran Branch, Poonak Tehran 1469669191 Iran; School of Natural Sciences – Chemistry, University of Tasmania Private Bag 75 Hobart TAS 7001 Australia Alireza.Ariafard@utas.edu.au

## Abstract

Although Pd(OAc)_2_-catalysed alkoxylation of the C(sp^3^)–H bonds mediated by hypervalent iodine(iii) reagents (ArIX_2_) has been developed by several prominent researchers, there is no clear mechanism yet for such crucial transformations. In this study, we shed light on this important issue with the aid of the density functional theory (DFT) calculations for alkoxylation of butyramide derivatives. We found that the previously proposed mechanism in the literature is not consistent with the experimental observations and thus cannot be operating. The calculations allowed us to discover an unprecedented mechanism composed of four main steps as follows: (i) activation of the C(sp^3^)–H bond, (ii) oxidative addition, (iii) reductive elimination and (iv) regeneration of the active catalyst. After completion of step (i) *via* the CMD mechanism, the oxidative addition commences with an X ligand transfer from the iodine(iii) reagent (ArIX_2_) to Pd(ii) to form a square pyramidal complex in which an iodonium occupies the apical position. Interestingly, a simple isomerization of the resultant five-coordinate complex triggers the Pd(ii) oxidation. Accordingly, the movement of the ligand trans to the Pd–C(sp^3^) bond to the apical position promotes the electron transfer from Pd(ii) to iodine(iii), resulting in the reduction of iodine(iii) concomitant with the ejection of the second X ligand as a free anion. The ensuing Pd(iv) complex then undergoes the C–O reductive elimination by nucleophilic attack of the solvent (alcohol) on the sp^3^ carbon *via* an outer-sphere S_N_2 mechanism assisted by the X^−^ anion. Noteworthy, starting from the five coordinate complex, the oxidative addition and reductive elimination processes occur with a very low activation barrier (Δ*G*^‡^ 0–6 kcal mol^−1^). The strong coordination of the alkoxylated product to the Pd(ii) centre causes the regeneration of the active catalyst, *i.e.* step (iv), to be considerably endergonic, leading to subsequent catalytic cycles to proceed with a much higher activation barrier than the first cycle. We also found that although, in most cases, the alkoxylation reactions proceed *via* a Pd(ii)–Pd(iv)–Pd(ii) catalytic cycle, the other alternative in which the oxidation state of the Pd(ii) centre remains unchanged during the catalysis could be operative, depending on the nature of the organic substrate.

## Abstract 2

This work uses DFT calculations to explore Pd(ii)-catalysed iodine(iii)-mediated alkoxylation of unactivated C(sp^3^)–H bonds and reveals how important the isomerization is in triggering the oxidative addition of ArIX_2_ to Pd(ii).

## Graphical abstract

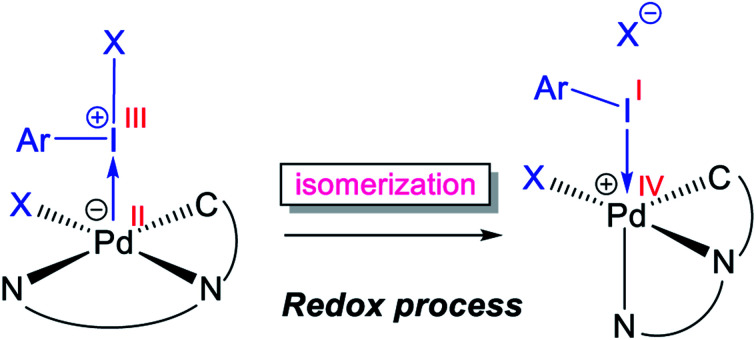

## Introduction

Hypervalent iodine compounds have been widely used in organic synthesis as oxidants and reaction promoters over recent decades.^1^ Although these compounds themselves have the capacity to mediate many organic reactions, the addition of a catalyst is usually a prerequisite for certain transformations to occur.^2^ Among these catalysts, palladium complexes have been demonstrated to have great potential to promote numerous iodine(iii)-mediated processes including the C–H functionalization and coupling reactions.^3^

**Scheme 1:**
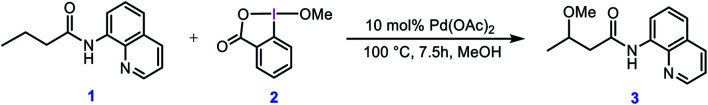
Palladium-catalyzed C(sp^3^)–H alkoxylation using the cyclic iodine(iii) reagent **2** (BI–OMe) developed by Rao *et al.*

In this context, Rao *et al.* developed a method for alkoxylation of unactivated methylene and methyl C(sp^3^)–H bonds to prepare alkyl ethers using hypervalent iodine(iii) reagents in the presence of a Pd(ii) catalyst.^4^ According to the developed method, they showed that butyramide derivative **1** is alkoxylated in the presence of an alcohol as the solvent and the alkoxylation reagent, methoxybenziodoxole (BI–OMe, **2**) as the oxidant, and Pd(OAc)_2_ as the catalyst (Scheme 1). This strategy for alkoxylation might receive specific attention due to its significance in modification of anti-inflammatory drugs such as ibuprofen and naproxen. It is worth mentioning that the importance of such a transformation has also prompted others to use similar methodologies for installing alkoxy groups onto other organic molecules using hypervalent iodine(iii) reagents under Pd(ii) catalysis even, in some cases, before that developed by Rao *et al.*^5,6^

**Scheme 2:**
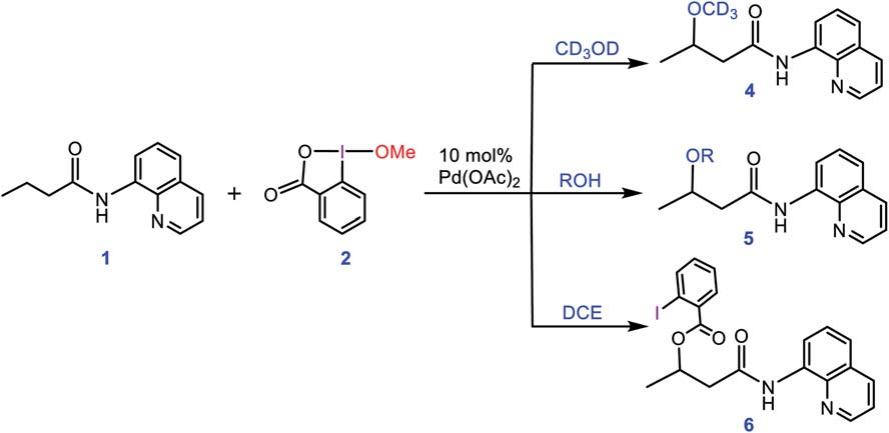
Different experiments performed by Rao *et al.* for confirming that the alkoxylation reagent is the alcohol solvent and the methoxy group of the oxidant **2** acts as a spectator.

In an attempt to explore the reaction mechanism, Rao *et al.* conducted an isotope labeling experiment in deuterated methanol (Scheme 2). On that basis, the only observed product was **4**, implying that the methoxy group installed on the product must originate from the solvent and not from the oxidant. Interestingly, when different alcohols were used as the solvent, only product **5** was practically observed and when the reaction was run in 1,2-dichloroethane (DCE), the carboxylated product **6** was attained. All these findings suggest that the methoxy group of the oxidant must act as a spectator ligand and thus is never involved in the reductive elimination step in the catalytic cycle (*vide infra*).

**Scheme 3:**
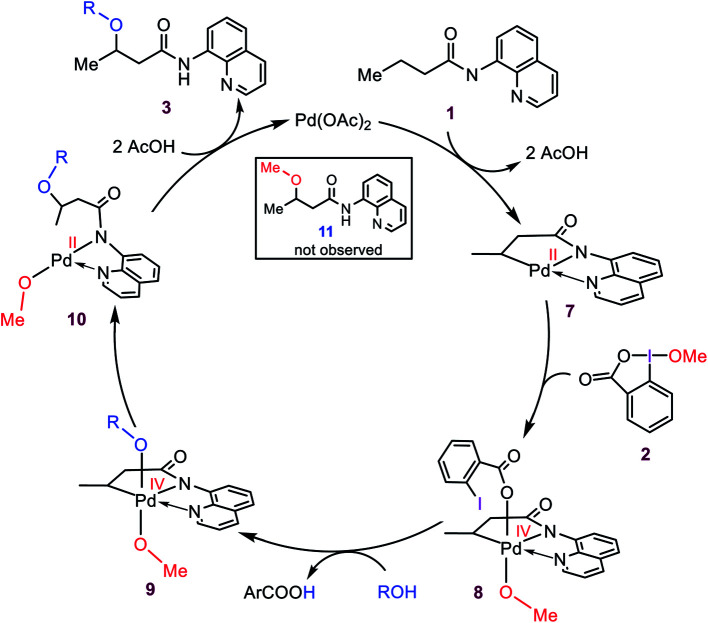
Catalytic cycle proposed by Rao *et al.*

Based on the preliminary results briefly discussed above, Rao *et al.* proposed the catalytic cycle outlined in Scheme 3. Accordingly, the reaction was surmised to commence with the coordination of substrate **1** to Pd(OAc)_2_, followed by OAc ligands-assisted N–H and C(sp^3^)–H activation processes *via* the concerted metallation–deprotonation (CMD) mechanism^7^ to yield cyclopalladated intermediate **7**. Subsequently, the oxidation of Pd(ii) to Pd(iv) by the cyclic iodine(iii) reagent **2** generates intermediate **8** from which a ligand exchange with the alcohol occurs to afford **9**. The resultant intermediate finally forms product **3** by undergoing the C–OR reductive elimination followed by a substitution reaction. It is worth noting that this plausible mechanism has also been summarized in several recent reviews,^3*e*,8^ and proposed by several other researchers for interpreting alkoxylation reactions mediated by iodine(iii) and catalyzed by palladium(ii).^4,5,6*e*,*f*,9*a*,*b*,*f*,*h*–*j*,*m*,*n*^

The above-simplified description prompted us to investigate the detailed mechanism of the title reaction by applying density functional theory (DFT) with the aim of addressing the following questions: (i) how the Pd(ii) is oxidized to the Pd(iv) by BI–OMe? (ii) Are **8** and **9** key intermediates on the catalytic cycle? If so, why does not the reductive elimination occur from **9** to give **11**? If not, what are the key intermediates? Why is the carboxylate group, and not the methoxy, installed on the organic molecule when the solvent is not the alcohol (Scheme 2)? Is the formation of a Pd(iv) intermediate inevitable in the catalytic process? Does the C–O reductive elimination take place *via* an inner-sphere mechanism? Why does the reaction require a high temperature to occur? Through answering these questions, we hope to enhance understanding of the fundamental processes involved in numerous Pd(ii)-catalyzed I(iii)-mediated alkoxylation reactions.^4,5,6*e-g*,9^

## Results and discussion

### CMD mechanism

**Fig. 1:**
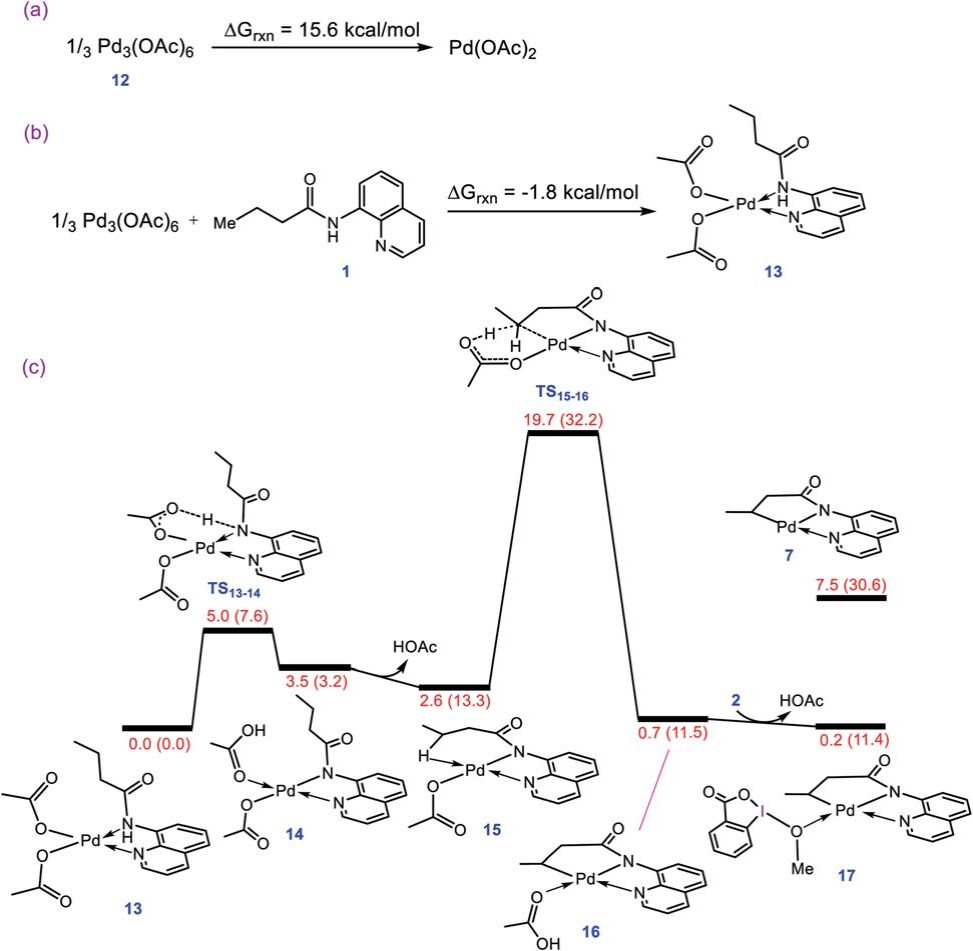
(a) Calculated reaction free energy for conversion of precatalyst **12** to Pd(OAc)_2_. (b) Calculated reaction free energy for formation of adduct **13** from precatalyst **12**. (c) Calculated energy profile for activation of the N–H and C–H bonds of substrate **1** by Pd(OAc)_2_. Free energies (potential energies) are given in kcal mol^−1^.

As described in the Introduction, the reaction is proposed to be initiated by activation of the N–H and C–H bonds of substrate **1** by Pd(OAc)_2_*via* the CMD mechanism. In analogy with the previous DFT studies,^10^ trimeric Pd_3_(OAc)_6_ is considered as the precatalyst for the palladium acetate; the trimeric form is computed to be 15.6 kcal mol^−1^ more stable than the monomeric Pd(OAc)_2_ (Fig. 1a). The breakdown of Pd_3_(OAc)_6_ into square planar complex **13** is computed to be exergonic by about −1.8 kcal mol^−1^ (Fig. 1b).

The N–H deprotonation of the coordinated substrate by one of the acetate ligands gives complex **14** in which acetic acid occupies a vacant coordination site of Pd(ii). This process is predicted to be extremely fast with Δ*G*^‡^ = 5.0 kcal mol^−1^. The acetic acid in this complex (**14**) binds weakly to the metal center and thus it can be easily replaced to give complex **15** with a Pd–H–C agostic interaction (Fig. 1c). The resultant complex then involves a second deprotonation process by the other acetate ligand to yield **16**. We found that this key step is nearly thermoneutral and proceeds with an overall activation barrier of 19.7 kcal mol^−1^. The replacement of the weakly bonded acetic acid in **16** by BI–OMe gives **17** as an active species on the catalytic cycle (*vide infra*).

### A preliminary evaluation of the mechanism proposed by Rao *et al.*

**Fig. 2:**
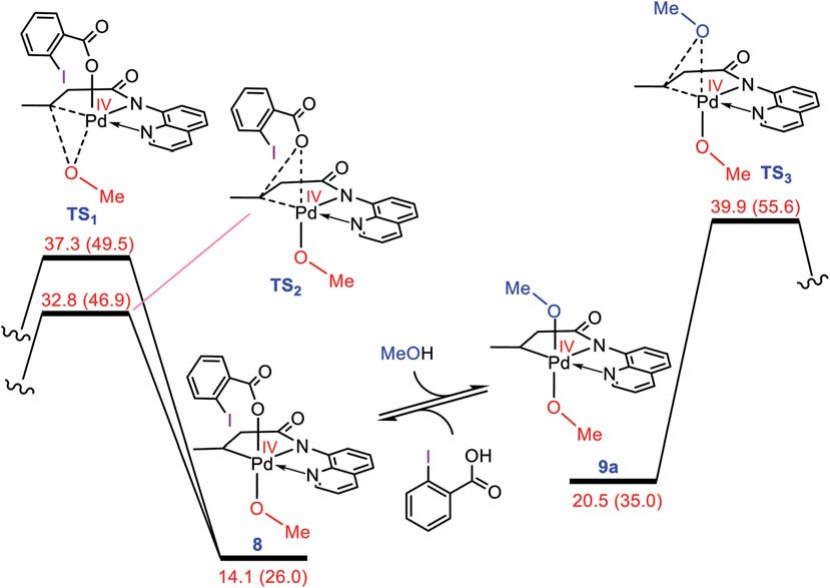
Calculated activation barrier to the reductive elimination from **8** and **9a**. Structure **13** is set as the reference point for this profile. Free energies (potential energies) are given in kcal mol^−1^.

As discussed in the Introduction, Rao *et al.* proposed **8** and **9** as the key intermediates responsible for the catalytic alkoxylation process (Scheme 3). On that basis, the reductive elimination from **9** is expected to produce both products **3** and **11**, which disagrees with the experimental findings. Similar key intermediates were also suggested by some other researchers for analogous alkoxylation reactions catalyzed by Pd(ii) complexes.^4,5,6*e*,*f*,9*a*,*b*,*f*,*h*–*j*,*m*,*n*^ To confirm the proposed mechanism is inoperative, we calculated the energy of intermediates **8** and **9a**, and then evaluated the C–O reductive elimination from these two intermediates (Fig. 2). Several trends are apparent from this DFT investigation. First, based on the proposed mechanism (Scheme 3), the ligand exchange between **8** and the alcohol (solvent) is a prerequisite for the reductive elimination to produce the desired product. Our calculations show that the ligand exchange process is endergonic by 6.4 kcal mol^−1^ where the alcohol is MeOH, implying that the Pd center prefers to remain coordinated with the carboxylate ligand. Second, transition structures **TS1** and **TS2** are significantly lower in energy than **TS3**. It follows that the reductive elimination should preferentially occur from **8** and not **9a**. Third, transition structure **TS2** is by 4.5 kcal mol^−1^ lower in energy than **TS1** indicating that **8** is more prone to be involved in the carbon–carboxylate coupling and not the carbon–alkoxy coupling.

It is inferred from the above results that the mechanism proposed in the literature is not capable of accounting for the experimental data. This inconsistency spurred us to seek for some other alternatives. The following discloses a novel mechanism by which one can easily interpret the results of the Pd(ii)-catalyzed I(iii)-mediated alkoxylation reactions developed by Rao *et al.* and others.^4,5,6*e-g*,9^

### Oxidative addition

**Fig. 3:**
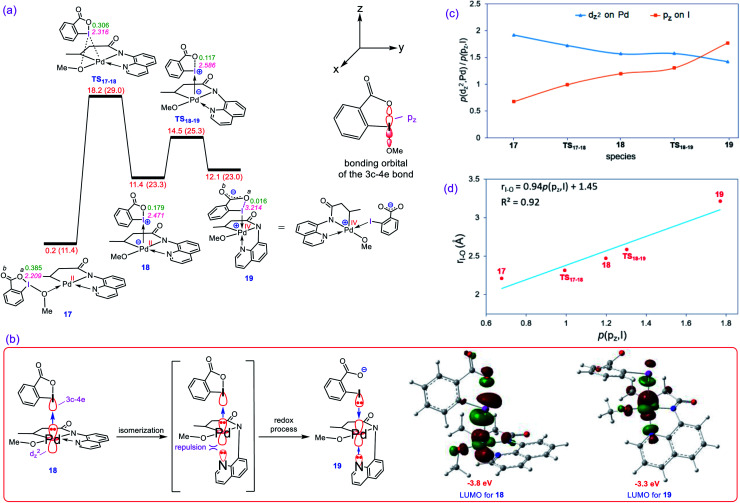
(a) Calculated energy profile for oxidation of Pd(ii) to Pd(iv) by BI–OMe. The I–O^*a*^ distances (Å) and the WBI values between I and O^*a*^ are annotated in pink and green, respectively. Free energies (potential energies) are given in kcal mol^−1^. (b) Important orbital interactions involved in the redox process and the spatial distribution of the LUMO orbitals for **18** and **19.** (c) Change in population of the palladium d_*z*^2^_ and iodine p_*z*_ orbitals upon moving from **17** to **19** obtained by NBO calculations. (d) Correlation between the population of iodine p_*z*_ orbital, *p*(p_*z*_, I), and the I–O^*a*^ distance.

Our calculations show that the oxidative addition step starts with the formation of adduct **17** in which reagent BI–OMe binds to the palladium center through its methoxy group (Fig. 3a). Next, the OMe ligand is transferred from the iodine to the palladium *via* transition structure **TS17-18** to give square pyramidal complex **18**. The resultant five coordinate complex is formally an iodonium salt in which the Pd d_*z*^2^_ orbital interacts with an empty orbital on the iodine(iii) having three-center-four-electron (3c-4e) character (Fig. 3b); the spatial distribution for the LUMO of **18** (Fig. 3b) indicates the antibonding orbital relating to such an interaction. Interestingly, we found that this complex is extremely reactive toward a redox process through undergoing a simple isomerization. The strong trans-influencing feature of the alkyl group causes the quinoline moiety of the tridentate ligand in **17** to coordinate relatively weakly to the Pd center and thus be highly susceptible to rearrangement. In this situation, the quinoline readily moves from the basal to the apical position *via* trigonal bipyramidal transition structure **TS18-19** lying only 3.1 kcal mol^−1^ above **18** to give another square pyramidal structure (**19**) in that the alkyl group occupies the apical position. This ligand movement turns on a repulsive interaction between the nitrogen lone pair and the filled d_*z*^2^_ orbital, destabilizing the d_*z*^2^_ orbital, resulting in two electrons being transferred from the Pd(ii) to the iodine(iii) center, furnishing **19**.^11^ As depicted in Fig. 3b, such an electron transfer turns off the Pd–N repulsive interaction and formally changes the oxidation state of the palladium center from +2 to +4. The spatial distribution for the LUMO of **19** (Fig. 3b) indicates the antibonding orbital relating to the simultaneous interaction of the lone pairs of the iodine and quinoline nitrogen atoms with the Pd(iv) d_*z*^2^_ orbital.

The population changes of the palladium d_*z*^2^_ and iodine p_*z*_ orbitals obtained by the NBO calculations for transformation **17** → **19** are shown in Fig. 3c. It follows from this figure that along the sequence, the Pd d_*z*^2^_ population decreases while the iodine p_*z*_ population increases. The considerable changes in the p_*z*_ population of the iodine atom supports the fact that the iodine(III) atom receives electrons from the Pd center and finally is reduced to the iodine(i) in **19**. Due to an increase in population of the iodine p_*z*_ orbital, the I–O^*a*^ bond distance becomes longer upon going from **17** to **19** (Fig. 3a) and finally is cleaved in **19** supported by Wiberg bond index (WBI) analysis showing an almost zero-bond order (0.016) between the I and O^*a*^ atoms. We found an excellent correlation between the I–O^*a*^ bond distance and the population of the iodine p_*z*_ orbital with *R*^2^ = 0.92 (Fig. 3d).^12^ These computational results are clearly consistent with the oxidation of Pd(ii) by the iodine(iii) reagent through the unprecedented mechanism outlined in Fig. 3a.

### Reductive elimination

**Fig. 4:**
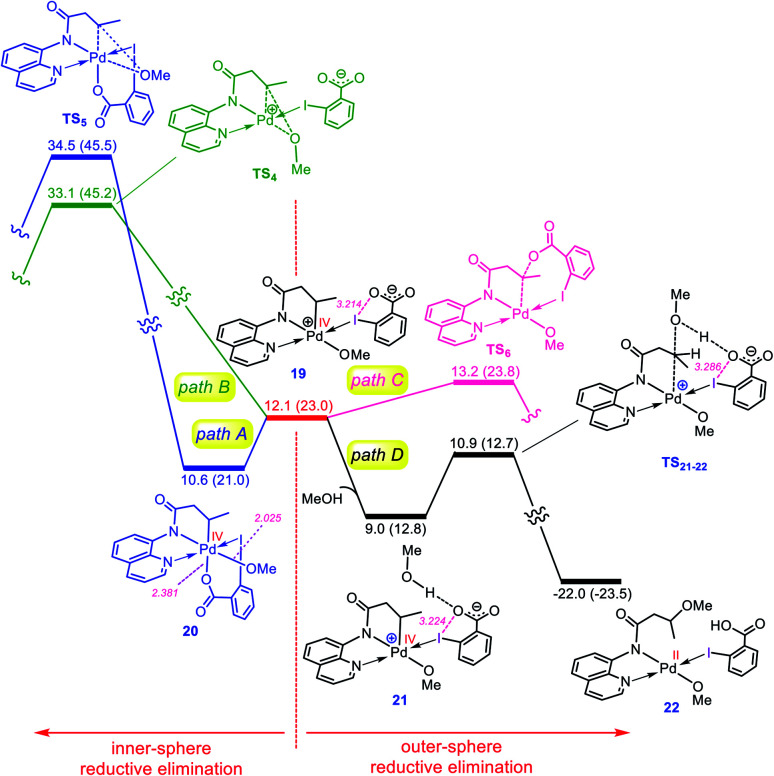
Calculated energy profile for C–O reductive elimination *via* pathways A–D. The selected distances (Å) are annotated in pink. Free energies (potential energies) are given in kcal mol^−1^.

Once the oxidative addition has taken place, the five-coordinate Pd(iv) complex **19** with a pendant carboxylate group is formed. The sixth coordination site of this Pd(iv) complex can be filled by this pendant carboxylate group to furnish complex **20** (Fig. 4). However, owing to the strong trans-influencing character of the alkyl moiety, the carboxylate binds weakly to the Pd(iv) center in **20**, indicating a comparable stability for these two intermediates (**19** and **20**).

Now, we turn our attention to investigation of the C–O reductive elimination from these two Pd(iv) complexes. Based on our calculations, this step can proceed *via* at least four different variants, as shown in Fig. 4. Accordingly, pathways A and B involve the inner-sphere reductive elimination from complexes **19** and **20** by crossing concerted transition structures **TS4** and **TS5**, respectively. These two pathways install the methoxy group of the oxidant on the final product. In pathway C, the C–O reductive elimination occurs through an outer-sphere S_N_2 mechanism involving the nucleophilic addition of the pendant carboxylate to the sp^3^ carbon bonded to the palladium by passing through transition structure **TS6**. This pathway leads to formation of the carboxylated product. In pathway D, a methanol pre-activated by the pendant carboxylate nucleophilically attacks the sp^3^ carbon to yield **22***via* transition structure **TS21-22**. In this pathway, the solvent serves as the alkoxylation reagent, and addition of the methanol to the sp^3^ carbon is accompanied by its deprotonation by the pendant carboxylate group. As can be seen from Fig. 4, **TS21-22** is much lower in energy than other transition structures, implying that pathway D is kinetically favored over pathways A, B, and C. However, when the solvent is not the alcohol, pathway D is turned off and thus, in this case, the reaction should preferentially proceed *via* pathway C, confirmed by the fact that **TS6** energetically lies considerably below **TS4** and **TS5**.

The energy profile given in Fig. 4 can answer several questions asked in the Introduction. For example, on that basis, one can explain why the OMe group on the oxidant does not appear in the product, why the solvent (alcohol) serves as the alkoxylation reagent and why when the solvent is not an alcohol, the carboxylate group is installed on the product.

### Regeneration of active catalyst **13**

**Fig. 5:**
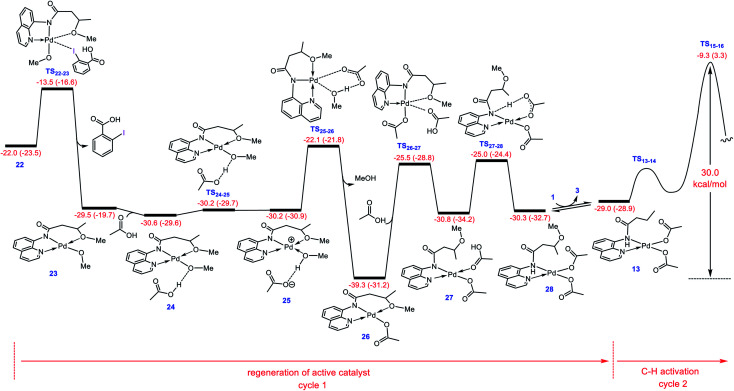
Calculated energy profile for regeneration of active catalyst **13** from **22**. Free energies (potential energies) are given in kcal mol^−1^.

After completion of the C–O reductive elimination *via* pathway D, intermediate **22** is formed. The dissociation of the carboxylic acid from the resulting intermediate *via* trigonal bipyramidal transition structure **TS22-23** yields **23** in which the methoxylated organic molecule acts as a tridentate ligand (Fig. 5). This complex subsequently participates in ligand exchange with one of the acetic acids produced from the CMD mechanism (Fig. 1) and generates the more stable intermediate **26** by passing through two transition structures **TS24-25** and **TS25-26**. The higher stability of **26** than **23** implies that the Pd(ii) center prefers to coordinate to the acetate rather than the methoxy ligand. The addition of the second acetic acid to **26***via* transition structure **TS26-27** gives **27** from which **28** is formed by a proton transfer from the coordinated acetic acid to the nitrogen atom of the methoxylated molecule. The overall activation barrier for formation of **28** from **22** is computed to be less than 15 kcal mol^−1^. Finally, displacement of the coordinated product by substrate **1** leads to regeneration of active catalyst **13**. From this point on, the second catalytic cycle begins. Since the active catalyst **13** lies 10.3 kcal mol^−1^ higher in energy than **26**, the overall activation barrier to the C(sp^3^)–H activation in the second catalytic cycle increases to 30.0 kcal mol^−1^ (Fig. 5). Indeed, the ability of the N–H deprotonated product to form a tridentate complex causes this species to bind more strongly to the palladium(ii) center than substrate **1**. This feature retards the alkoxylation reaction by decreasing the activity of the catalyst, resulting in the process requiring a high temperature for completion.

### Catalytic cycle proposed by the DFT calculations

**Scheme 4:**
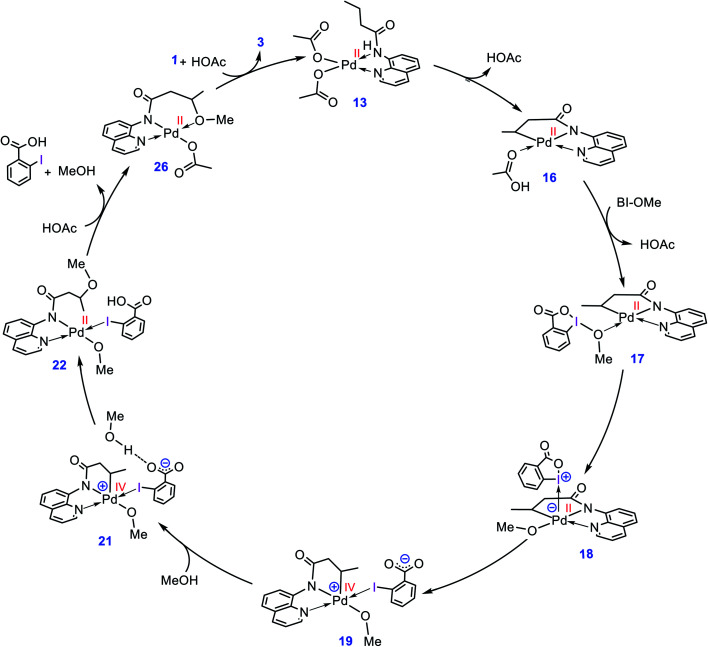
Catalytic cycle proposed by the DFT calculations for palladium-catalyzed C(sp^3^)–H alkoxylation using BI–OMe.

The catalytic cycle shown in Scheme 4 summarizes our calculation results related to the mechanism of the title reaction. The reaction is initiated by coordination of substrate **1** to the Pd complex followed by deprotonation of the N–H and C(sp^3^)–H bonds by the OAc ligands to give cyclopalladated intermediate **16**.^13^ Subsequently, the substitution of BI–OMe for HOAc affords **17**. The OMe ligand in the resultant intermediate (**17**) is then transferred from iodine(iii) to palladium(ii) to give iodonium **18** stabilized by an anionic palladium(ii) complex. Afterward, intermediate **18** undergoes isomerization by moving the quinoline moiety from the basal to the apical position, triggering a redox process by promoting two electrons from Pd(ii) to iodine(iii), leading to formation of Pd(iv) complex **19** with a pendant carboxylate group. This isomerization not only promotes the Pd(ii) oxidation but also sets the stage ready for the reductive elimination by formation of a five-coordinate complex in which the reacting alkyl moiety occupies a position trans to the empty site.^14^ The addition of a methanol (solvent) to the ensuing complex then affords **21**. In this intermediate, the methanol is stabilized by a hydrogen bonding interaction with the pendant carboxylate group. Next, the C–O reductive elimination takes place by nucleophilic attack of the methanol on the sp^3^ carbon *via* an outer-sphere S_N_2 mechanism to yield **22**. Our calculations predict that starting from iodonium salt **18**, the oxidative addition and the reductive elimination steps are extremely fast with Δ*G*^‡^ < 3.5 kcal mol^−1^ (Fig. 3 and 4). Later, the more stable complex **26** is furnished by following a series of chemical steps. The high stability of this species causes the regeneration of the active catalyst **13** to be considerably endergonic (∼10 kcal mol^−1^), resulting in the overall activation barrier to the subsequent catalytic cycle increasing to 30.0 kcal mol^−1^ (Fig. 5). This finding clearly explains why the alkoxylation reaction developed by Rao *et al.* requires a high temperature for completion (Scheme 1).

### Impact of the substituent on the reaction mechanism

**Scheme 5:**
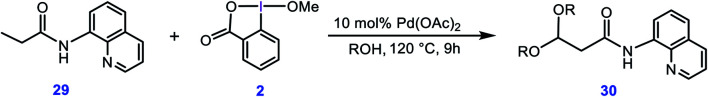
Palladium-catalyzed double alkoxylation of C(sp^3^)–H bonds using the cyclic iodine(iii) reagent **2** (BI–OMe) developed by Rao *et al.*

In a separate study, Rao *et al.* used a similar method for preparation of acetals through double alkoxylation of the C(sp^3^)–H bonds of butyramide derivative **29** using BI–OMe as the oxidant and Pd(OAc)_2_ as the catalyst (Scheme 5).^9*b*^ Based on the preliminary results, the authors proposed that the product of the first alkoxylation is the substrate for the second one.

**Fig. 6:**
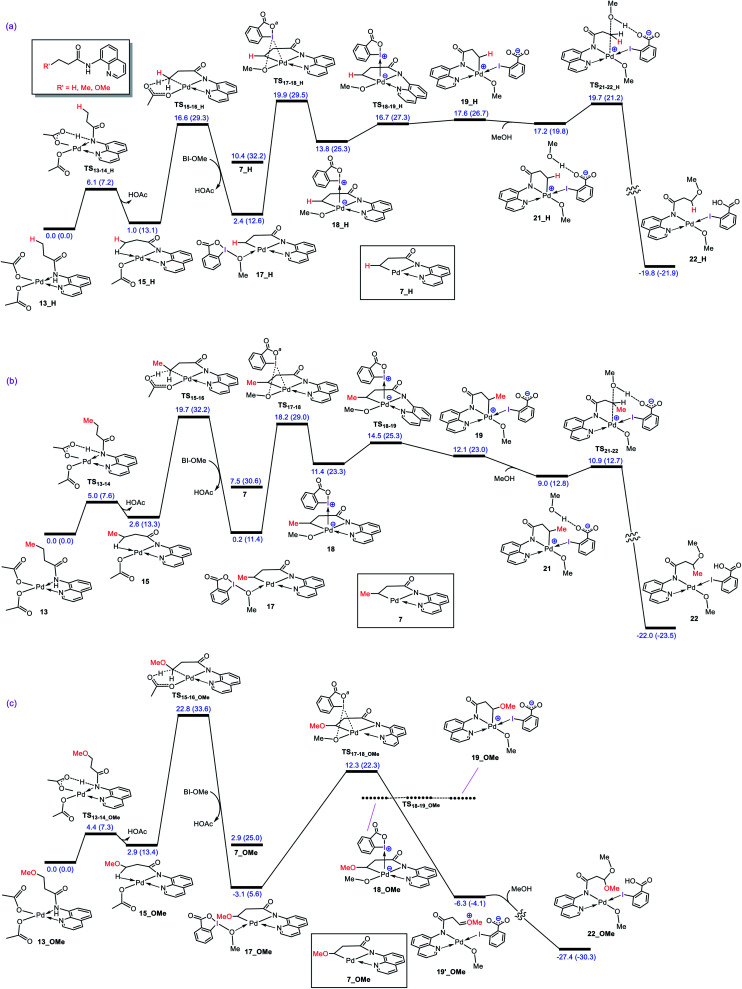
Calculated energy profiles for palladium-catalyzed alkoxylation of the C(sp^3^)–H bond of butyramide derivatives with different R′ substituent. Free energies (potential energies) are given in kcal mol^−1^.

**Scheme 6:**
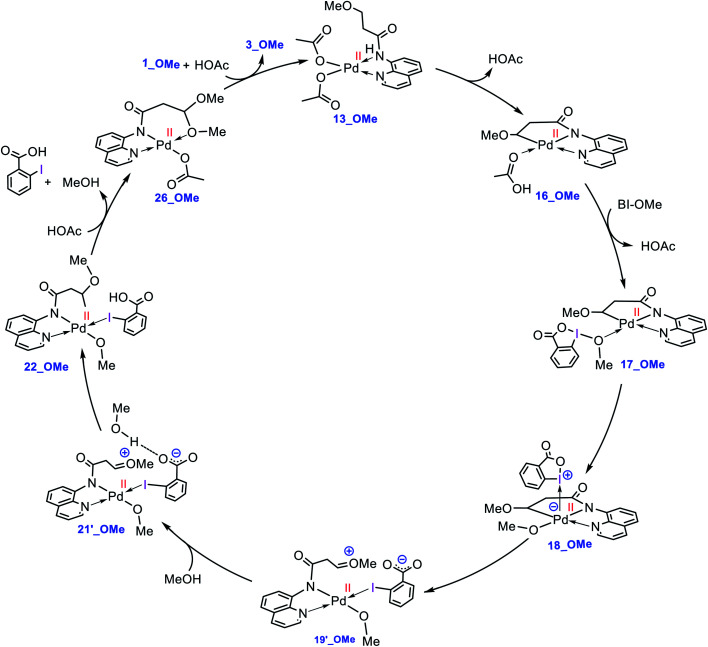
Catalytic cycle proposed by the DFT calculations for palladium-catalyzed C(sp^3^)−H alkoxylation of **1_OMe** using BI–OMe.

What interest us here is to explore how the electronic feature of the R′ substituent on a substrate affects the alkoxylation mechanism. Fig. 6 compares the energy profiles for alkoxylation of the substrates with R′ = H, Me, and OMe. Several points emerge from this comparison. First, the overall activation energy of the C–H activation step increases in the order R′ = H < Me < OMe. It is inferred from this result that the greater the electron donor property of the R group, the higher the activation barrier of the C–H activation. Indeed, a substituent with strong electron donating ability decreases the acidity of the hydrogen being abstracted, leading to the C–H activation to become more energy demanding. Second, the stability of three coordinate complex **7** (**7_R′**) is determined by the nature of the R′ group; an R′ group with strong electron donating ability increases the intrinsic stability of this coordinatively unsaturated species. It also finds that the relative energy of the transition structure **TS17-18** (**TS17-18_R′**) depends on the intrinsic stability of this three coordinate species and decreases in the order R = H > Me > OMe. Third, although the formation of a Pd(iv) complex is unavoidable for the substrates with R′ = H and Me, this is not the case for the substrate with R′ = OMe. The IRC calculation shows that transition structure **TS17-18_OMe** directly connects **17-OMe** to **19′_OMe**. It follows that intermediates **18_OMe** and **19_OMe** are not local minimum and thus no Pd(iv) intermediate is formed in this case. Indeed, if we assume that **19_OMe** is generated during the reaction, it is highly unstable and rapidly undergoes a redox process. This is because of the strong π-donor property of the OMe substituent, forcing the Pd^IV^–C(sp^3^) σ-bond in **19_OMe** to be completely polarized toward the palladium center, giving zwitterion complex **19′_OMe** in which the Pd center bears an oxidation state of +2. In this case, the C–O coupling process from this zwitterion complex does not involve the reductive elimination step, and instead, it takes place *via* nucleophilic addition of the carboxylate-activated MeOH to the oxonium ion. The DFT calculations show that the addition of the methanol to the oxonium ion is extremely fast and proceeds without involvement of an intermediate. As a result, it is evident from our calculations that a change in the R substituent leads to an abrupt alteration in the reaction mechanism. Scheme 6 shows the modified catalytic cycle for alkoxylation of the substrate with R = OMe.

### Assessing the generality of our proposed mechanism

**Scheme 7:**
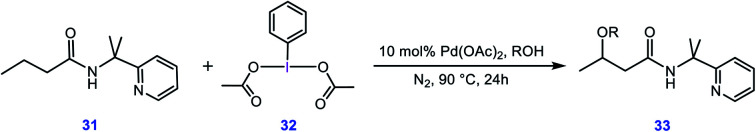
Palladium-catalyzed C(sp^3^)–H alkoxylation using the iodine(iii) reagent **32** (PIDA) developed by Shi *et al.*

**Fig. 7:**
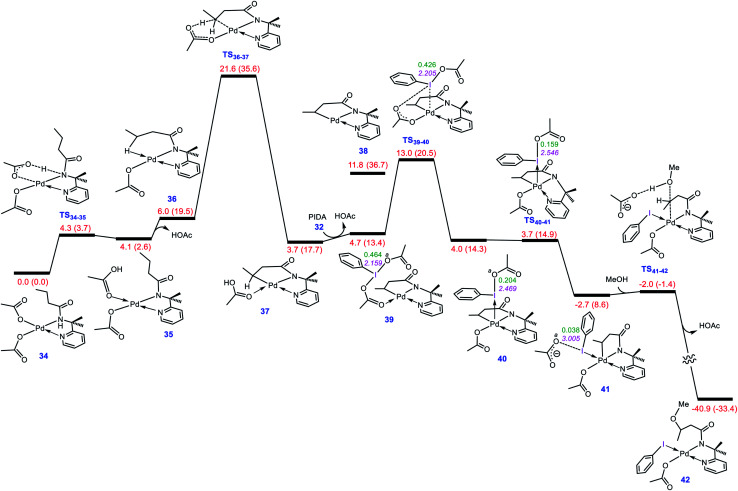
Calculated energy profile for palladium-catalyzed C(sp^3^)–H alkoxylation using the iodine(iii) reagent PIDA. The I–O^*a*^ distances (Å) and the WBI values between I and O^*a*^ are annotated in pink and green, respectively. Free energies (potential energies) are given in kcal mol^−1^.

To evaluate whether the mechanism proposed in this study is applicable to interpret other alkoxylation reactions catalysed by Pd(ii) complexes, we investigated the mechanism of the reaction shown in Scheme 7 developed by Shi.^5^ In this reaction, iodobenzenediacetate (PIDA) is used as the oxidant. The energy profile given in Fig. 7 indicates that the reaction is commenced by activation of the N–H and C–H bonds by the OAC ligands by surmounting an activation barrier of 21.6 kcal mol^−1^. The coordination of PIDA to the Pd(ii) centre then sets the stage for the redox process. The transfer of the acetate ligand from the iodine to the Pd atom *via* transition structure **TS39-40** gives iodonium ion **40**. The overall activation barrier for this crucial step is calculated to be only 13.0 kcal mol^−1^, implying that similar to BI–OMe, PIDA is susceptible to forming the key iodonium intermediate. The ensuing species is extremely elusive and involves the required isomerization in a barrierless fashion to furnish **41** with a relative free energy of −2.7 kcal mol^−1^. The isomerization triggers the oxidation of Pd(ii) to Pd(iv) through electron transfer to the iodine(iii) atom. The redox process causes the I–O^*a*^ bond in **41** to be cleaved, supported by the negligible WBI value (0.038) between the I and O^*a*^ atoms. Starting from **41**, addition of the methanol to the sp^3^ carbon atom takes place *via* transition structure **TS41-42** with a free energy barrier as low as 0.7 kcal mol^−1^. According to our calculation results, once the OAc transfer from the iodine to the palladium has taken place *via* transition structure **TS39-40**, the oxidative addition and reductive elimination steps proceed in a barrierless fashion. These additional results lend support to the generality of our novel mechanism found by the DFT calculations for Pd(ii)-catalyzed I(III)-mediated alkoxylation of C(sp^3^)–H bonds.

## Conclusion

In this work, we performed DFT calculations to elucidate the mechanism of the alkoxylation of the C(sp^3^)–H bonds using hypervalent iodine(iii) reagents (ArIX_2_) catalysed by Pd(OAc)_2_. An unprecedented mechanism for this transformation is revealed by clarifying the oxidative addition step. The calculations indicate that this key step begins with the transfer of an X ligand from ArIX_2_ to a Pd–alkyl intermediate. The ligand transfer forms a square pyramidal Pd(ii) complex in which iodonium [ArIX]^+^ occupies the apical position and is stabilized by interaction with the Pd d_*z*^2^_ orbital. The resultant complex then undergoes an isomerization by moving the ligand trans to the Pd–alkyl bond to the apical position. This isomerization considerably destabilizes the d_*z*^2^_ orbital, promoting the electron transfer from Pd(ii) to I(iii), resulting in the reduction of I(iii) concomitant with the extrusion of the second X ligand as a free anion. The released anion then assists the alcohol (solvent) to nucleophilically attack the Pd(iv)–alkyl bond *via* an S_N_2 mechanism to form a new C–O bond. The information reported in this study is important in enhancing our understanding of the fundamental processes that underpin many catalytic reactions mediated by iodine(iii) reagents and catalyzed by Pd(ii) complexes and could assist scientists to design new catalytic reactions.

## Computational details

Gaussian 16 (ref. 15) was used to fully optimize all the structures reported in this paper at the M06 level of theory.^16^ For all the calculations, solvent effects were considered using the SMD solvation model^17^ with methanol as the solvent. The SDD basis set^18^ with effective core potential (ECP) was chosen to describe iodine and palladium. The 6-31G(d) basis set^19^ was used for other atoms. This basis set combination will be referred to as BS1. Frequency calculations were carried out at the same level of theory as those for the structural optimization. Transition structures were located using the Berny algorithm. Intrinsic reaction coordinate (IRC) calculations were used to confirm the connectivity between transition structures and minima.^20^ To further refine the energies obtained from the SMD/M06/BS1 calculations, we carried out single-point energy calculations in methanol using the M06 functional method for all of the structures with a larger basis set (BS2). BS2 utilizes the def2-TZVP basis set^21^ on all atoms with an effective core potential including scalar relativistic effect for palladium and iodine. Tight convergence criterion was also employed to increase the accuracy of the calculations. In this work, the free energy for each species in solution was calculated using the following formula:

1 *G* = *E*(BS2) + *G*(BS1) − *E*(BS1) + Δ*G*^1 atm→1 M^

where Δ*G*^1 atm→1 M^ = 1.89 kcal mol^−1^ is the free-energy change for compression of 1 mol of an ideal gas from 1 atm to the 1 M solution phase standard state.

An additional correction to Gibbs free energies was made to consider methanol concentration where a MeOH is directly involved in transformations. In such a case, the free energy of MeOH is described as follows:

2 *G*(MeOH) = *E*(BS2) + *G*(BS1) − *E*(BS1) + Δ*G*^1 atm→1 M^ + *RT* ln(24.72)

where the last term corresponds to the free energy required to change the standard state of MeOH from 24.72 M to 1 M.^22^

The orbital population analysis and determination of the WBI bond orders were carried out by the NBO7 program.^23^

## Author contributions

P. A., S. K. T. and K. F. performed the calculations. A. A. supervised the work and wrote the manuscript with contribution from the other authors.

## Conflicts of interest

There are no conflicts to declare.

## Supplementary Material

SC-012-D1SC01230D-s001
