# Supplementary material for: The role of hypervalent iodine(iii) reagents in promoting alkoxylation of unactivated C(sp3)–H bonds catalyzed by palladium(ii) complexes
Source: Chem Sci. 2021 Apr 14;12(20):7185–95. doi: 10.1039/d1sc01230d (PMC8153247; doi:10.1039/d1sc01230d)
Supplement: SC-012-D1SC01230D-s001 [file SC-012-D1SC01230D-s001.pdf]

Electronic Supplementary Material (ESI) for Chemical Science.
This journal is © The Royal Society of Chemistry 2021

*"Supporting Information for"*

# The role of hypervalent iodine(III) reagents in promoting alkoxylation of unactivated C(sp3)–H bonds catalyzed by palladium(II) complexes

Payam Abdolalian, ‡ Samaneh K. Tizhoush, ‡ Kaveh Farshadfar, ‡ Alireza Ariafard†*

‡*Department of Chemistry, Islamic Azad University, Central Tehran Branch, Poonak, Tehran, 1469669191, Iran*
†*School of Natural Sciences - Chemistry, University of Tasmania, Private Bag 75, Hobart, TAS 7001, Australia*

**Content:**

**Fig. S1** Comparing the calculated mechanisms of oxidative addition from **18** *via* two different pathways. (page S2)
**Fig S2.** Wave function analysis carried out along the IRC of $\mathbf{TS_{17\text{-}18}}$ and $\mathbf{TS_{18\text{-}19}}$. (page S3)
**Fig. S3** Comparing the calculated mechanisms of cyclopalladation via removal of α-, β- and γ-C-H protons by the OAc ligand. (page S4)
**Table S1**. Total potential (E), enthalpy (H) and Gibbs free energies (G) of all structures optimized at the SMD/M06/BS1 level of theory along with the total potential energies calculated by SMD/M06/BS2//SMD/M06/BS1 and Cartesian coordinates for all of the calculated structures. (page S5)

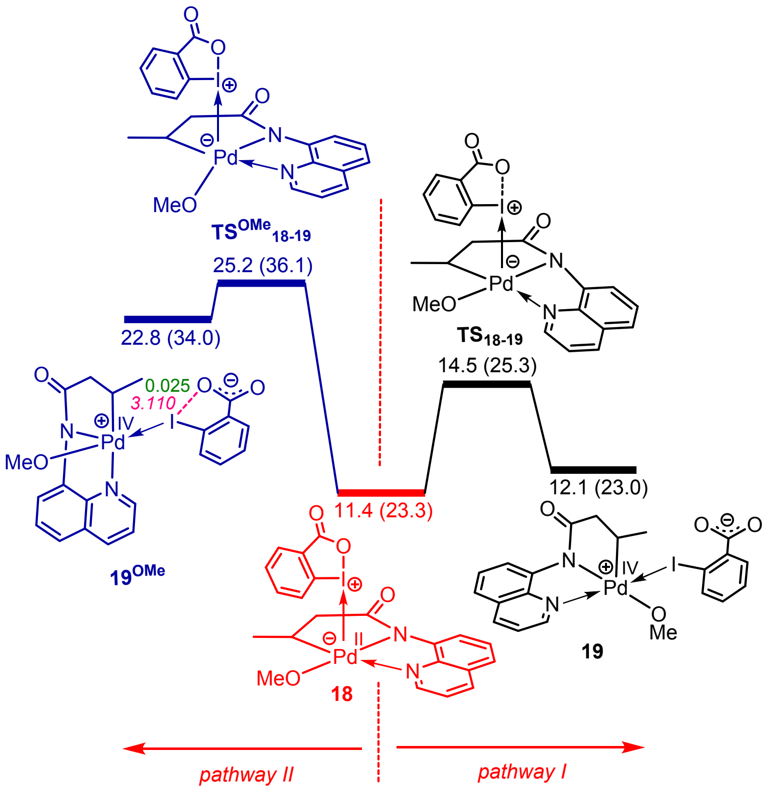


**Fig. S1** Comparing the calculated mechanisms of oxidative addition from **18** *via* pathways I and II. Free energies (potential energies) are given in kcal/mol. The I-O distance (Å) and the WBI value between I and O are annotated in pink and green, respectively. Pathway I involves the migration of the quinoline to the apical position while in pathway II, the isomerization occurs via the movement of the OMe ligand. Although both pathways lead to oxidation of the Pd(II) center, pathway I is computed to be much more favorable than pathway II. This result supports the importance of the isomerization through moving the ligand trans to the strong σ-donor alkyl ligand, as discussed in the oxidative addition section.

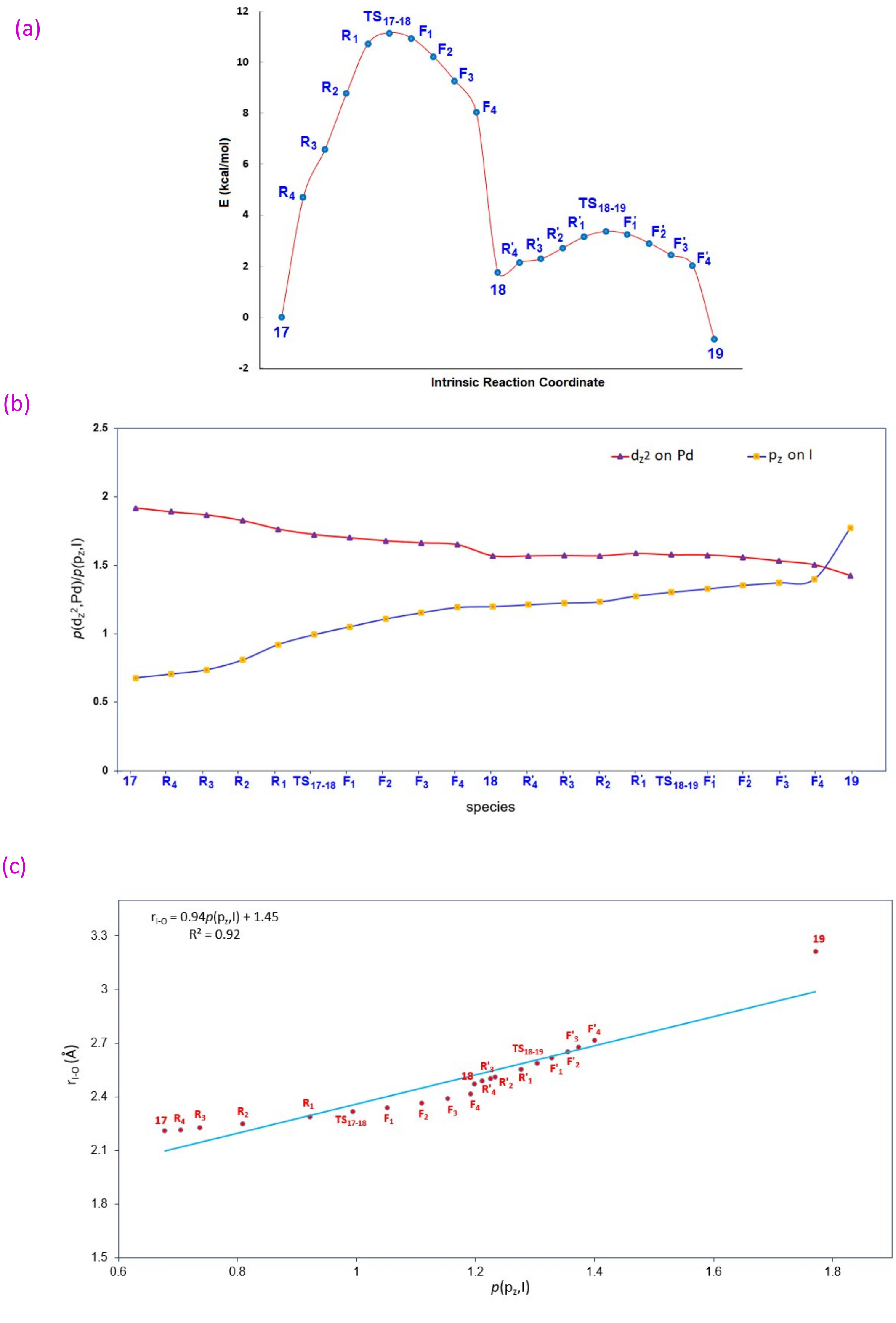


**Fig. S2** (a) IRC path for oxidation of Pd(II) to Pd(IV) by BI-OMe (transformation **17** → $\mathbf{TS_{17\text{-}18}}$ → **18** → $\mathbf{TS_{18\text{-}19}}$ → **19**) calculated at the SDD/M06/BS1 level of theory. (b) Change in population of the palladium $d_z{}^2$ and iodine $p_z$ orbitals upon moving from **17** to **19** obtained by NBO calculations. (d) Correlation between the population of iodine $p_z$ orbital, $p(p_z, \text{I})$, and the I-O[a] distance.

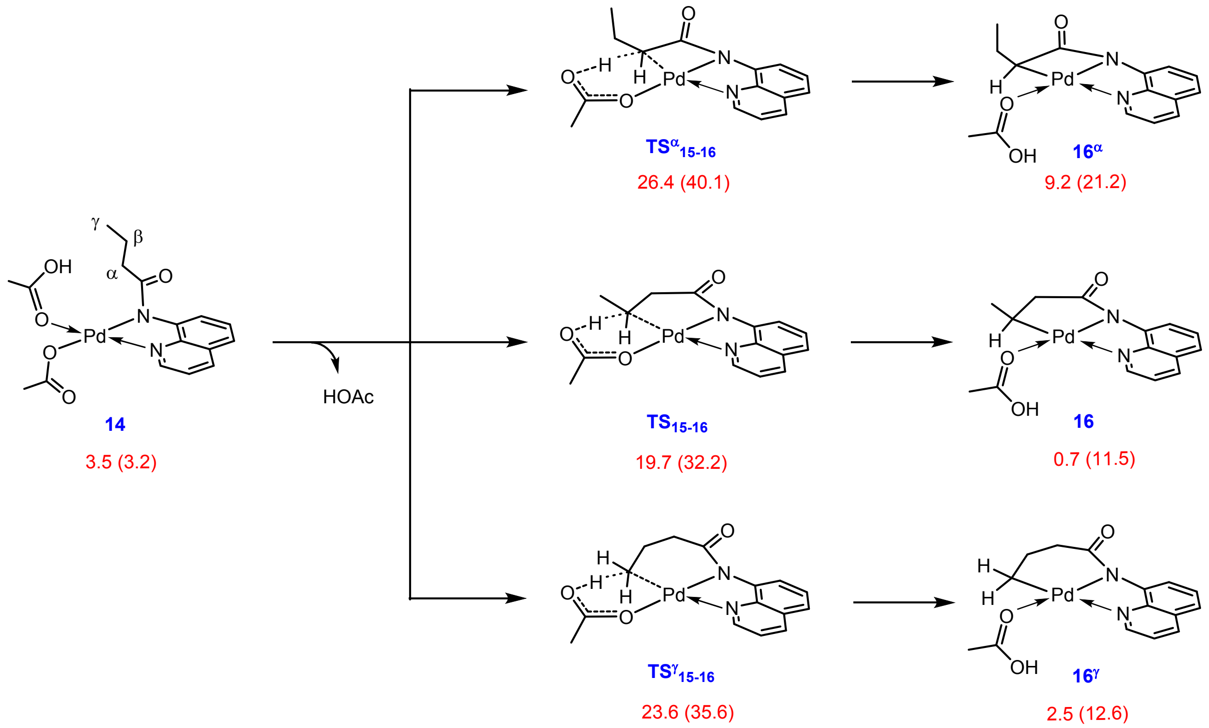


**Fig. S3** Comparing the calculated mechanisms of cyclopalladation via removal of α-, β- and γ-C-H protons by the OAc ligand. Free energies (potential energies) are given in kcal/mol. It follows that **TS**$_{\mathbf{15\text{-}16}}$ is significantly lower in energy than **TS**$^{\alpha}_{\mathbf{15\text{-}16}}$ and **TS**$^{\gamma}_{\mathbf{15\text{-}16}}$, implying that the C-H activation at the β position is kinetically favoured over other positions. Since **TS**$_{\mathbf{15\text{-}16}}$ lies below **TS**$_{\mathbf{17\text{-}18}}$, **TS**$_{\mathbf{18\text{-}19}}$ and **TS**$_{\mathbf{21\text{-}22}}$ (Figs 3a and 4), the C-H activation in this case is unlikely to be reversible and thus the regioselectivity of the alkoxylation reaction should be controlled by a kinetic preference for deprotonation of the β-C-H bond.

**Table S1**. Total potential (E), enthalpy (H) and Gibbs free energies (G) of all structures optimized at the SMD/M06/BS1 level of theory along with the total potential energies calculated by SMD/M06/BS2//SMD/M06/BS1 and Cartesian coordinates for all of the calculated structures.

**1**
E (M06-SMD/BS1) = -688.0848934 au
H (M06-SMD/BS1) = -687.822439 au
G (M06-SMD/BS1) = -687.879235 au
E (M06-SMD/BS2//M06-SMD/BS1) = -688.3457287 au

| | | | |
|---|---|---|---|
| C | 0.10878900 | 1.51496100 | -0.28906900 |
| C | -0.22434700 | 0.17848100 | -0.19636500 |
| C | -1.60661100 | -0.17953900 | -0.03689900 |
| C | -2.59018800 | 0.84088200 | 0.05016300 |
| C | -2.20599500 | 2.20016900 | -0.02329200 |
| C | -0.88295100 | 2.51490600 | -0.19348000 |
| H | 1.13442500 | 1.82780600 | -0.44880100 |
| C | -3.93475900 | 0.43124800 | 0.20647000 |
| H | -2.97006900 | 2.97338800 | 0.04964300 |
| H | -0.57306100 | 3.55619000 | -0.26441200 |
| C | -4.23679700 | -0.90487800 | 0.26198100 |
| C | -3.18421300 | -1.83715700 | 0.15911300 |
| H | -4.71294900 | 1.19134500 | 0.27823000 |
| H | -5.26028600 | -1.25310900 | 0.38013900 |
| H | -3.40446400 | -2.90582700 | 0.19652200 |
| N | -1.91996100 | -1.49990800 | 0.01755800 |
| N | 0.64616000 | -0.91117700 | -0.27376800 |
| H | 0.14346100 | -1.80049100 | -0.29910900 |
| C | 2.00447200 | -1.07760500 | -0.18804400 |
| C | 2.89417300 | 0.12123100 | -0.02131900 |
| H | 2.81654100 | 0.74169500 | -0.92900600 |
| H | 2.50720300 | 0.74270000 | 0.80218900 |
| C | 4.34789100 | -0.24274300 | 0.22957500 |
| H | 4.72043300 | -0.85272900 | -0.60577500 |
| H | 4.41616700 | -0.87727600 | 1.12543900 |
| C | 5.20745600 | 0.99698400 | 0.39779400 |
| H | 4.86590900 | 1.60687400 | 1.24648100 |
| H | 6.25830400 | 0.73614700 | 0.57755900 |
| H | 5.17073400 | 1.63157300 | -0.49939200 |
| O | 2.45284400 | -2.22279200 | -0.24450200 |

**2** (BI-OMe)
E (M06-SMD/BS1) = -545.7135263 au
H (M06-SMD/BS1) = -545.566294 au
G (M06-SMD/BS1) = -545.617131 au

E (M06-SMD/BS2//M06-SMD/BS1) = -832.3361573 au

| | | | |
|---|---|---|---|
| C | -2.93306700 | 0.60943400 | 0.11731200 |
| C | -1.73941000 | -0.11061000 | 0.06520900 |
| C | -0.56254800 | 0.59754800 | -0.10691500 |
| C | -0.50256400 | 1.96962600 | -0.24512500 |
| C | -1.70894800 | 2.66807500 | -0.18972800 |
| C | -2.91472000 | 1.99300100 | -0.00627000 |
| H | -3.86643700 | 0.06542700 | 0.25283400 |
| H | 0.44669500 | 2.47874300 | -0.39778500 |
| H | -1.70053400 | 3.75159700 | -0.29393300 |
| H | -3.84692500 | 2.55281500 | 0.03515000 |
| I | 1.18775700 | -0.62872300 | -0.15655800 |
| C | -1.70146400 | -1.59996200 | 0.17117900 |
| O | -2.73049200 | -2.25551500 | 0.33617200 |
| O | -0.51921500 | -2.11991000 | 0.06964700 |
| O | 2.36312200 | 1.03535900 | -0.32982500 |
| C | 2.78933800 | 1.55094300 | 0.92062900 |
| H | 3.39550400 | 2.43940400 | 0.70094200 |
| H | 1.94299400 | 1.85393700 | 1.55770800 |
| H | 3.41054600 | 0.83260400 | 1.47695500 |

**3**

E (M06-SMD/BS1) = -802.5368415 au
H (M06-SMD/BS1) = -802.239691 au
G (M06-SMD/BS1) = -802.303405 au
E (M06-SMD/BS2//M06-SMD/BS1) = -802.8443413 au

| | | | |
|---|---|---|---|
| C | -1.63872400 | 2.26884500 | 0.03603400 |
| C | -1.10504500 | 1.09505600 | 0.51861700 |
| C | -1.78492300 | -0.14074100 | 0.30394600 |
| C | -2.99627800 | -0.12244700 | -0.44557300 |
| C | -3.51253100 | 1.10109100 | -0.93223000 |
| C | -2.84862100 | 2.27672000 | -0.68716200 |
| H | -1.10697200 | 3.20111000 | 0.22201400 |
| C | -3.65127300 | -1.35758800 | -0.66050000 |
| H | -4.44428400 | 1.08861600 | -1.49760000 |
| H | -3.24427900 | 3.22128300 | -1.05540100 |
| C | -3.11366300 | -2.50547900 | -0.14119500 |
| C | -1.91624300 | -2.40989400 | 0.60016500 |
| H | -4.57712700 | -1.37219200 | -1.23575100 |
| H | -3.58823500 | -3.47363000 | -0.28363600 |
| H | -1.48211700 | -3.31518200 | 1.03056000 |

| | | | |
|---|---|---|---|
| N | -1.26706100 | -1.28683600 | 0.82192000 |
| N | 0.08430300 | 1.13692300 | 1.29008900 |
| C | 1.29187400 | 0.56317700 | 1.02362000 |
| C | 1.45739700 | -0.09115700 | -0.32605100 |
| H | 1.11989800 | -1.13326000 | -0.22782200 |
| H | 0.78911400 | 0.38278500 | -1.05779000 |
| C | 2.87191300 | -0.08662900 | -0.88703600 |
| H | 2.79980900 | -0.48566400 | -1.91787800 |
| C | 3.48726000 | 1.30047200 | -0.95454700 |
| H | 2.79467600 | 1.99457600 | -1.44955300 |
| H | 3.70072700 | 1.68212500 | 0.05186600 |
| H | 4.42085900 | 1.30170200 | -1.53061700 |
| O | 2.19015100 | 0.61183900 | 1.86501000 |
| O | 3.64683300 | -0.99766100 | -0.12129500 |
| C | 4.89851200 | -1.29068200 | -0.69175400 |
| H | 5.60842200 | -0.45239000 | -0.60849900 |
| H | 5.31334200 | -2.14779200 | -0.14912300 |
| H | 4.80229700 | -1.56055100 | -1.75799100 |
| H | 0.03937400 | 1.59997600 | 2.19543500 |

**$Pd_3(OAc)_6$**

E (M06-SMD/BS1) = -1754.105696 au

H (M06-SMD/BS1) = -1753.756817 au

G (M06-SMD/BS1) = -1753.864489 au

E (M06-SMD/BS2//M06-SMD/BS1) = -1754.737721 au

| | | | |
|---|---|---|---|
| Pd | -1.79376100 | 0.05833100 | -0.00038000 |
| O | -1.93306900 | 1.20231200 | -1.68086200 |
| O | -2.05882800 | 1.66595200 | 1.25226600 |
| C | -1.17749700 | 2.31228700 | 1.88706000 |
| O | 0.07208900 | 2.27780800 | 1.67841800 |
| Pd | 0.94613200 | 1.52227700 | -0.00058100 |
| C | -1.26857400 | 2.26109700 | -1.88971600 |
| O | -0.24075300 | 2.63496600 | -1.25624700 |
| O | -2.00982800 | -1.07482600 | 1.67950600 |
| O | -2.16675200 | -1.52717500 | -1.25155100 |
| C | -1.32996900 | -2.23455800 | -1.88138400 |
| O | -0.08070100 | -2.28080400 | -1.67342400 |
| Pd | 0.84791700 | -1.58229500 | 0.00151400 |
| C | -1.41006400 | -2.17055000 | 1.89461700 |
| O | -0.40580700 | -2.60901900 | 1.26516900 |
| O | 1.94875500 | -1.19978900 | 1.67516800 |

| | | | |
|---|---|---|---|
| O | 2.39957700 | -1.11208700 | -1.26160400 |
| C | 2.59860000 | -0.13199100 | 1.88469000 |
| C | 2.58737000 | -0.03459900 | -1.89519400 |
| O | 2.47216200 | 0.95563100 | 1.25342200 |
| O | 2.00626300 | 1.07112100 | -1.68144200 |
| C | -1.77126700 | 3.16832100 | -2.96880000 |
| H | -2.46582700 | 3.88838400 | -2.51602800 |
| H | -0.94711300 | 3.72443800 | -3.42508700 |
| H | -2.31673400 | 2.60004400 | -3.72824900 |
| C | -1.86505100 | -3.13038300 | -2.95455600 |
| H | -2.10643100 | -4.10127900 | -2.50169100 |
| H | -2.77805500 | -2.71521200 | -3.39099400 |
| H | -1.11075200 | -3.29589500 | -3.72988500 |
| C | -1.97015800 | -3.03994400 | 2.97673000 |
| H | -1.19300400 | -3.67873100 | 3.40610000 |
| H | -2.43828200 | -2.43040200 | 3.75578500 |
| H | -2.74459800 | -3.68172000 | 2.53609000 |
| C | 3.63885300 | -0.17638800 | 2.95997500 |
| H | 3.75144100 | 0.80429700 | 3.43164200 |
| H | 3.39087900 | -0.93520800 | 3.70809400 |
| H | 4.59744300 | -0.44930000 | 2.49888400 |
| C | 3.61826000 | -0.05336900 | -2.98027400 |
| H | 4.59072000 | 0.19398700 | -2.53410000 |
| H | 3.68902700 | -1.04659600 | -3.43319100 |
| H | 3.39165400 | 0.69942100 | -3.74133700 |
| C | -1.65464400 | 3.23444900 | 2.96522800 |
| H | -2.57510200 | 2.85729200 | 3.42043200 |
| H | -0.88026600 | 3.37361900 | 3.72558400 |
| H | -1.86827500 | 4.21152300 | 2.51170400 |

**$Pd(OAc)_2$**

E (M06-SMD/BS1) = -584.6589628 au

H (M06-SMD/BS1) = -584.544567 au

G (M06-SMD/BS1) = -584.59454 au

E (M06-SMD/BS2//M06-SMD/BS1) = -584.8738101 au

| | | | |
|---|---|---|---|
| Pd | 0.00003200 | 0.00004500 | -0.01574500 |
| O | -1.76187800 | -1.08333600 | -0.01316900 |
| C | -2.43934000 | -0.00002100 | -0.00199700 |
| O | -1.76190300 | 1.08341200 | -0.01314400 |
| O | 1.76191200 | 1.08328100 | -0.01359600 |
| C | 2.43933000 | -0.00013200 | -0.00221700 |

| | | | |
|---|---|---|---|
| O | 1.76174200 | -1.08338900 | -0.01358200 |
| C | -3.91522900 | -0.00003400 | 0.05424200 |
| H | -4.22654500 | -0.00374200 | 1.10739000 |
| H | -4.31555800 | 0.90161300 | -0.42004900 |
| H | -4.31560600 | -0.89852800 | -0.42602300 |
| C | 3.91518800 | -0.00008600 | 0.05463900 |
| H | 4.31572300 | -0.90214400 | -0.41870200 |
| H | 4.31568100 | 0.89799600 | -0.42631200 |
| H | 4.22613000 | 0.00463800 | 1.10788900 |

**13**

E (M06-SMD/BS1) = -1272.796451 au

H (M06-SMD/BS1) = -1272.417225 au

G (M06-SMD/BS1) = -1272.503352 au

E (M06-SMD/BS2//M06-SMD/BS1) = -1273.266857 au

| | | | |
|---|---|---|---|
| C | 2.86726400 | -1.07327200 | 1.63780900 |
| C | 1.82154700 | -0.46804400 | 0.98610300 |
| C | 1.99305500 | 0.78683800 | 0.37041500 |
| C | 3.25141200 | 1.43143600 | 0.42380600 |
| C | 4.31387400 | 0.79031100 | 1.10249000 |
| C | 4.12368400 | -0.43435700 | 1.69554200 |
| H | 2.71896800 | -2.04512300 | 2.10795300 |
| C | 3.36893200 | 2.68921100 | -0.21026300 |
| H | 5.28124300 | 1.28854600 | 1.14436500 |
| H | 4.94294900 | -0.92405200 | 2.21669800 |
| C | 2.28405000 | 3.23613300 | -0.84923700 |
| C | 1.06633600 | 2.53472000 | -0.86172300 |
| H | 4.32547400 | 3.20917500 | -0.18312400 |
| H | 2.34197600 | 4.20007900 | -1.34673400 |
| H | 0.18545400 | 2.93554800 | -1.36113400 |
| N | 0.92765200 | 1.35749200 | -0.27582200 |
| N | 0.50858200 | -1.08171600 | 0.90218000 |
| H | 0.04423700 | -1.17053800 | 1.81728600 |
| C | 0.38144500 | -2.33426100 | 0.19260000 |
| C | 1.21110800 | -2.49284700 | -1.04342600 |
| H | 2.12354900 | -3.04450500 | -0.76040100 |
| H | 1.54468500 | -1.51205000 | -1.41068200 |
| C | 0.45396300 | -3.24816600 | -2.12913100 |
| H | 0.15945300 | -4.23523200 | -1.74722400 |
| H | -0.48012100 | -2.70704300 | -2.35131400 |
| C | 1.28970900 | -3.39444700 | -3.38655400 |

| | | | |
|---|---|---|---|
| H | 1.56869800 | -2.41295100 | -3.79544800 |
| H | 0.74364500 | -3.93816400 | -4.16766600 |
| H | 2.21909100 | -3.94486700 | -3.18241000 |
| O | -0.41348800 | -3.15062100 | 0.60241100 |
| Pd | -0.77680000 | 0.25520600 | -0.13174400 |
| O | -1.90028900 | 1.60206900 | -1.16410800 |
| C | -2.50164700 | 2.46437300 | -0.40002500 |
| O | -2.40659200 | 2.49016600 | 0.83132700 |
| O | -2.44288800 | -0.90362000 | 0.04783700 |
| C | -2.75347700 | -1.23723000 | 1.26332200 |
| O | -2.13672200 | -0.86918300 | 2.27097200 |
| C | -3.31840500 | 3.47556700 | -1.15835400 |
| H | -2.64395900 | 4.13792400 | -1.71703300 |
| H | -3.92236100 | 4.07708800 | -0.47261000 |
| H | -3.96734600 | 2.97908600 | -1.88893100 |
| C | -3.93042800 | -2.16892000 | 1.35737400 |
| H | -4.33400100 | -2.17924300 | 2.37450300 |
| H | -3.59281300 | -3.18341200 | 1.10386200 |
| H | -4.71433200 | -1.89292800 | 0.64373900 |

**$TS_{13\text{-}14}$**

E (M06-SMD/BS1) = -1272.784221 au

H (M06-SMD/BS1) = -1272.410737 au

G (M06-SMD/BS1) = -1272.49522 au

E (M06-SMD/BS2//M06-SMD/BS1) = -1273.255069 au

| | | | |
|---|---|---|---|
| C | 2.50651100 | 0.24680700 | 1.98384200 |
| C | 1.49527600 | 0.28057800 | 1.05266500 |
| C | 1.13974500 | 1.51292700 | 0.46101000 |
| C | 1.84895300 | 2.69627800 | 0.77206900 |
| C | 2.89411800 | 2.62551500 | 1.72281200 |
| C | 3.20271400 | 1.42772800 | 2.32115300 |
| H | 2.77631100 | -0.70251200 | 2.44557400 |
| C | 1.45305500 | 3.88544100 | 0.11819600 |
| H | 3.43596000 | 3.53601800 | 1.97447600 |
| H | 3.99928800 | 1.37706400 | 3.06055000 |
| C | 0.41096700 | 3.86209300 | -0.77574900 |
| C | -0.26844300 | 2.65443700 | -1.01164400 |
| H | 1.98289800 | 4.81183800 | 0.33662500 |
| H | 0.08563900 | 4.75939800 | -1.29476700 |
| H | -1.11971900 | 2.60372600 | -1.68880500 |
| N | 0.08363900 | 1.52819900 | -0.41577700 |

| | | | |
|---|---|---|---|
| N | 0.78266700 | -0.88578800 | 0.62090400 |
| H | 0.04891500 | -1.54482200 | 1.41455500 |
| C | 1.43384300 | -1.79424600 | -0.25364800 |
| C | 2.73941400 | -1.37915000 | -0.87193900 |
| H | 3.48704000 | -1.26192700 | -0.07139100 |
| H | 2.61461300 | -0.36949100 | -1.29925800 |
| C | 3.24207200 | -2.35290300 | -1.92327000 |
| H | 3.35873800 | -3.34826300 | -1.47106400 |
| H | 2.48403100 | -2.46097200 | -2.71315900 |
| C | 4.55908100 | -1.89054400 | -2.51997800 |
| H | 4.45544200 | -0.90639600 | -2.99901900 |
| H | 4.92456100 | -2.59334400 | -3.27949400 |
| H | 5.33599000 | -1.80092100 | -1.74718400 |
| O | 0.90537000 | -2.86997100 | -0.48742000 |
| Pd | -0.95164300 | -0.21283300 | -0.32919100 |
| O | -2.59378200 | 0.52404000 | -1.29158700 |
| C | -3.41743300 | 1.17452800 | -0.52784100 |
| O | -3.26144000 | 1.34140300 | 0.68710000 |
| O | -2.02594300 | -1.92858800 | 0.11507300 |
| C | -1.77102800 | -2.53987000 | 1.19651400 |
| O | -0.78442800 | -2.28122600 | 1.95839900 |
| C | -4.59478700 | 1.75631200 | -1.26344600 |
| H | -4.24847900 | 2.58465600 | -1.89597300 |
| H | -5.34267100 | 2.13478100 | -0.56027200 |
| H | -5.04828800 | 1.00866500 | -1.92419000 |
| C | -2.67326100 | -3.65967200 | 1.59634000 |
| H | -2.15389000 | -4.60853300 | 1.40839600 |
| H | -3.60563000 | -3.64325700 | 1.02605900 |
| H | -2.88231400 | -3.60508200 | 2.67022900 |

**14**

E (M06-SMD/BS1) = -1272.790432 au
H (M06-SMD/BS1) = -1272.412662 au
G (M06-SMD/BS1) = -1272.496878 au
E (M06-SMD/BS2//M06-SMD/BS1) = -1273.262157 au

| | | | |
|---|---|---|---|
| C | -2.49010400 | 1.25001500 | -1.51615500 |
| C | -1.43152200 | 0.91162500 | -0.69617800 |
| C | -0.59698500 | 1.95533400 | -0.22447100 |
| C | -0.88569900 | 3.31448700 | -0.48785300 |
| C | -2.00723000 | 3.62162000 | -1.29241700 |
| C | -2.77402800 | 2.60263700 | -1.80324000 |

| | | | |
|---|---|---|---|
| H | -3.11490200 | 0.47106800 | -1.94742000 |
| C | -0.01397500 | 4.28375700 | 0.05854400 |
| H | -2.23135200 | 4.66462100 | -1.51031900 |
| H | -3.62265400 | 2.83135300 | -2.44531200 |
| C | 1.07209300 | 3.89131500 | 0.80179400 |
| C | 1.32137500 | 2.52122000 | 0.98674200 |
| H | -0.21529200 | 5.33885000 | -0.12361900 |
| H | 1.75763200 | 4.61465600 | 1.23461500 |
| H | 2.19173700 | 2.16849900 | 1.53731200 |
| N | 0.51706200 | 1.59637100 | 0.48966800 |
| N | -1.05886500 | -0.39070100 | -0.29704400 |
| H | -0.26447400 | -1.63963900 | -1.54606300 |
| C | -1.92406100 | -1.27431100 | 0.30474000 |
| C | -3.39855400 | -0.94954000 | 0.40918200 |
| H | -3.86012000 | -1.10776800 | -0.57852700 |
| H | -3.53421100 | 0.11932500 | 0.63176000 |
| C | -4.12286300 | -1.79749500 | 1.44353100 |
| H | -4.01613100 | -2.86061800 | 1.18682400 |
| H | -3.64234500 | -1.66865900 | 2.42511500 |
| C | -5.59259300 | -1.42597400 | 1.52839600 |
| H | -5.72263000 | -0.37202200 | 1.81336700 |
| H | -6.12030700 | -2.03851600 | 2.27085600 |
| H | -6.09498200 | -1.56937600 | 0.56078900 |
| O | -1.49560600 | -2.34263100 | 0.75550900 |
| Pd | 0.89273700 | -0.38149300 | 0.35438000 |
| O | 2.79348700 | -0.20672900 | 1.10791500 |
| C | 3.70369400 | 0.07491600 | 0.22955600 |
| O | 3.48871900 | 0.22079500 | -0.98037300 |
| O | 1.39759600 | -2.38770700 | -0.05493900 |
| C | 0.97564300 | -2.99835700 | -1.04921600 |
| O | 0.08700500 | -2.51061800 | -1.87797600 |
| C | 5.08074500 | 0.24906800 | 0.81327200 |
| H | 5.10197200 | 1.16637400 | 1.41683300 |
| H | 5.83266900 | 0.32590100 | 0.02215500 |
| H | 5.32649300 | -0.58509200 | 1.48065300 |
| C | 1.44400800 | -4.36447700 | -1.38344900 |
| H | 0.62350200 | -5.07053800 | -1.20153300 |
| H | 2.30375000 | -4.63716100 | -0.76766500 |
| H | 1.69908400 | -4.42220500 | -2.44738800 |

**15**

E (M06-SMD/BS1) = -1043.797608 au
H (M06-SMD/BS1) = -1043.488065 au
G (M06-SMD/BS1) = -1043.559153 au
E (M06-SMD/BS2//M06-SMD/BS1) = -1044.179531 au

| | | | |
|---|---|---|---|
| C | -3.07704800 | 1.66597900 | 0.22314200 |
| C | -1.90648900 | 0.93814400 | 0.06276100 |
| C | -2.01879500 | -0.47723800 | 0.00100700 |
| C | -3.26673600 | -1.13709400 | 0.09773700 |
| C | -4.43361100 | -0.35733700 | 0.26106900 |
| C | -4.32191100 | 1.01017800 | 0.32031700 |
| H | -3.03403700 | 2.74669000 | 0.27499100 |
| C | -3.26982900 | -2.54853200 | 0.02734100 |
| H | -5.39824900 | -0.85618000 | 0.33859100 |
| H | -5.21461000 | 1.62052500 | 0.44735200 |
| C | -2.09208000 | -3.23735500 | -0.12916900 |
| C | -0.88730500 | -2.52245300 | -0.21711800 |
| H | -4.21981700 | -3.07686100 | 0.10023500 |
| H | -2.06776500 | -4.32210100 | -0.18534700 |
| H | 0.07203600 | -3.02117800 | -0.34125800 |
| N | -0.86351900 | -1.20192100 | -0.15383200 |
| N | -0.59389700 | 1.41886600 | -0.04138800 |
| C | -0.24969500 | 2.73669500 | -0.05450800 |
| C | 1.22528100 | 3.04772700 | -0.19343300 |
| H | 1.42926400 | 3.91216500 | 0.45418400 |
| H | 1.38073500 | 3.40319100 | -1.22484500 |
| C | 2.21260000 | 1.93966800 | 0.12272500 |
| H | 2.03360500 | 1.53218700 | 1.12885900 |
| H | 2.16744800 | 1.13692700 | -0.68639400 |
| C | 3.66639000 | 2.37916900 | 0.02875700 |
| H | 3.90039500 | 2.76494700 | -0.97191900 |
| H | 4.34477100 | 1.54547000 | 0.24706800 |
| H | 3.85837000 | 3.17736800 | 0.75827600 |
| O | -1.05992900 | 3.66816700 | 0.01093800 |
| Pd | 0.77184700 | -0.03761200 | -0.23698800 |
| O | 2.04011600 | -1.62256100 | -0.55304700 |
| C | 2.96516200 | -1.80407400 | 0.33718000 |
| O | 3.11352200 | -1.10275100 | 1.34494600 |
| C | 3.86362800 | -2.97694800 | 0.04735900 |
| H | 3.27437400 | -3.90269900 | 0.05690600 |
| H | 4.66243000 | -3.04947400 | 0.79145200 |
| H | 4.30097600 | -2.88144500 | -0.95387100 |

**$TS_{15\text{-}16}$**

E (M06-SMD/BS1) = -1043.767238 au

H (M06-SMD/BS1) = -1043.463434 au

G (M06-SMD/BS1) = -1043.531714 au

E (M06-SMD/BS2//M06-SMD/BS1) = -1044.149361 au

| | | | |
|---|---|---|---|
| C | -3.00387500 | 1.80766400 | 0.17628700 |
| C | -1.89143700 | 0.98615800 | 0.06675700 |
| C | -2.10885300 | -0.42061300 | -0.01143600 |
| C | -3.41290900 | -0.97063800 | 0.02858800 |
| C | -4.51904900 | -0.09815200 | 0.14391700 |
| C | -4.30114500 | 1.25630600 | 0.21389700 |
| H | -2.87170700 | 2.88186600 | 0.23458100 |
| C | -3.53193100 | -2.37699000 | -0.05296900 |
| H | -5.52400100 | -0.51656600 | 0.17316000 |
| H | -5.14725700 | 1.93644200 | 0.30012000 |
| C | -2.40997700 | -3.15975500 | -0.16659300 |
| C | -1.14819200 | -2.54223000 | -0.20053400 |
| H | -4.52545700 | -2.82360700 | -0.02463800 |
| H | -2.47419900 | -4.24260800 | -0.23137300 |
| H | -0.23432600 | -3.12775800 | -0.28930900 |
| N | -1.00760500 | -1.23044500 | -0.12668600 |
| N | -0.55053100 | 1.37225900 | 0.01491100 |
| C | -0.06899700 | 2.62428700 | 0.23223200 |
| C | 1.43898900 | 2.70302200 | 0.19093900 |
| C | 2.12089100 | 1.56916100 | -0.56203100 |
| H | 1.71642600 | 1.50404200 | -1.58548200 |
| O | -0.76061100 | 3.62192900 | 0.47199400 |
| Pd | 0.74451800 | -0.13176000 | -0.21048400 |
| O | 2.05511300 | -1.77394100 | -0.29166800 |
| C | 3.09578300 | -1.55027800 | 0.39475500 |
| O | 3.35482900 | -0.40338000 | 0.88056700 |
| C | 4.05509900 | -2.66235200 | 0.65528700 |
| H | 5.06185200 | -2.35774400 | 0.34694000 |
| H | 4.08946700 | -2.85925900 | 1.73399900 |
| H | 3.76172000 | -3.57385900 | 0.12822500 |
| H | 2.53094000 | 0.43054000 | 0.28506200 |
| C | 3.61648500 | 1.84457800 | -0.73191900 |
| H | 4.12690800 | 1.98665400 | 0.23022500 |
| H | 4.12530400 | 1.03620700 | -1.27200800 |
| H | 3.73926300 | 2.76972100 | -1.31579600 |

| | | | |
|---|---|---|---|
| H | 1.78910100 | 2.72576000 | 1.23723000 |
| H | 1.71518600 | 3.68055700 | -0.23648000 |

**16**

E (M06-SMD/BS1) = -1043.800633 au

H (M06-SMD/BS1) = -1043.491717 au

G (M06-SMD/BS1) = -1043.562273 au

E (M06-SMD/BS2//M06-SMD/BS1) = -1044.18242 au

| | | | |
|---|---|---|---|
| C | -3.22079500 | 1.40189200 | 0.17861800 |
| C | -1.98198500 | 0.78656400 | 0.05055900 |
| C | -1.95503100 | -0.64699600 | -0.02312700 |
| C | -3.15662300 | -1.39903900 | 0.04379400 |
| C | -4.39344300 | -0.72786900 | 0.17640600 |
| C | -4.40695500 | 0.64357500 | 0.24031600 |
| H | -3.27066400 | 2.48329000 | 0.23469700 |
| C | -3.05418900 | -2.80800300 | -0.02719700 |
| H | -5.31180000 | -1.31172900 | 0.22384600 |
| H | -5.35368400 | 1.17282000 | 0.34124500 |
| C | -1.82617400 | -3.40572000 | -0.15621800 |
| C | -0.68351100 | -2.58796900 | -0.21811700 |
| H | -3.96573000 | -3.40358300 | 0.02262400 |
| H | -1.71977200 | -4.48613600 | -0.21235800 |
| H | 0.31122900 | -3.02409900 | -0.32400000 |
| N | -0.74301100 | -1.27123800 | -0.15610100 |
| N | -0.74005600 | 1.41885000 | 0.00315500 |
| C | -0.50693600 | 2.75412800 | 0.13740300 |
| C | 0.97247500 | 3.05430500 | 0.21450100 |
| H | 1.16054200 | 4.07195500 | -0.16505300 |
| H | 1.21381500 | 3.08551900 | 1.29237100 |
| C | 1.84656600 | 2.00501000 | -0.46506200 |
| H | 1.84160100 | 2.16558100 | -1.55724300 |
| C | 3.26295700 | 1.99156100 | 0.06064200 |
| H | 3.29664100 | 1.73175800 | 1.13015900 |
| H | 3.90250300 | 1.28240300 | -0.48156300 |
| H | 3.72682500 | 2.98949900 | -0.03926100 |
| O | -1.36976300 | 3.63563000 | 0.24206300 |
| Pd | 0.84154800 | 0.23337300 | -0.30104600 |
| O | 2.70525100 | -1.44229800 | 1.53367800 |
| C | 3.08751100 | -1.61672100 | 0.28342000 |
| O | 2.54139800 | -1.04468500 | -0.66460900 |
| C | 4.23207100 | -2.54474000 | 0.12259400 |

| | | | |
|---|---|---|---|
| H | 4.01867400 | -3.49179600 | 0.63147200 |
| H | 4.43535900 | -2.71962700 | -0.93606600 |
| H | 5.11698100 | -2.10717000 | 0.60186000 |
| H | 1.95701400 | -0.79614800 | 1.55574300 |

**17**

E (M06-SMD/BS1) = -1360.549985 au

H (M06-SMD/BS1) = -1360.160991 au

G (M06-SMD/BS1) = -1360.250657 au

E (M06-SMD/BS2//M06-SMD/BS1) = -1647.452167 au

| | | | |
|---|---|---|---|
| C | 5.29265000 | 0.32695600 | 0.61249300 |
| C | 3.94359600 | 0.22216500 | 0.29508700 |
| C | 3.44997500 | -1.06620100 | -0.10402800 |
| C | 4.32071100 | -2.18735900 | -0.15255300 |
| C | 5.68525500 | -2.03073300 | 0.17992200 |
| C | 6.14560800 | -0.79197100 | 0.55130100 |
| H | 5.68892700 | 1.28887200 | 0.91624600 |
| C | 3.77104200 | -3.43263900 | -0.53582300 |
| H | 6.34479200 | -2.89652100 | 0.13498600 |
| H | 7.19523300 | -0.65704300 | 0.80958800 |
| C | 2.43818500 | -3.53108900 | -0.84227500 |
| C | 1.64623100 | -2.37104200 | -0.77451400 |
| H | 4.42405000 | -4.30438500 | -0.57791900 |
| H | 1.98624700 | -4.47558600 | -1.13471800 |
| H | 0.58162600 | -2.41674800 | -1.01090400 |
| N | 2.12339900 | -1.18964400 | -0.42845300 |
| N | 2.99954900 | 1.24544000 | 0.34854700 |
| C | 3.23141800 | 2.53356500 | 0.72822800 |
| C | 1.94938400 | 3.32579600 | 0.82881100 |
| H | 2.16306800 | 4.39078900 | 0.64373100 |
| H | 1.64967400 | 3.26051800 | 1.89040700 |
| C | 0.81470800 | 2.79548400 | -0.04347000 |
| H | 0.90559900 | 3.19510900 | -1.06846000 |
| C | -0.54721800 | 3.11067400 | 0.53259200 |
| H | -0.68883300 | 2.63045600 | 1.51369600 |
| H | -1.36707800 | 2.78855600 | -0.12326400 |
| H | -0.66409300 | 4.19824800 | 0.69028900 |
| O | 4.33148900 | 3.01934300 | 1.02272100 |
| Pd | 1.15962300 | 0.80080700 | -0.30328400 |
| C | -6.00513800 | 0.16022300 | 0.18855200 |
| C | -4.74262300 | -0.38423100 | 0.42135500 |

| | | | |
|---|---|---|---|
| C | -3.66287700 | 0.15323100 | -0.25922600 |
| C | -3.76224900 | 1.20395700 | -1.14903100 |
| C | -5.03562700 | 1.73248900 | -1.36588200 |
| C | -6.14769300 | 1.21436900 | -0.70448100 |
| H | -6.86073800 | -0.25441700 | 0.71906100 |
| H | -2.88906200 | 1.60755800 | -1.65696600 |
| H | -5.15204300 | 2.56121200 | -2.06184300 |
| H | -7.13203400 | 1.64075000 | -0.88633400 |
| I | -1.81778000 | -0.80339300 | 0.24394800 |
| C | -4.52563500 | -1.50230000 | 1.37899400 |
| O | -5.44592400 | -2.02542200 | 1.99730800 |
| O | -3.27908400 | -1.87386300 | 1.50748600 |
| O | -0.82285400 | 0.45457200 | -1.12030200 |
| C | -0.76923600 | -0.04014300 | -2.45802000 |
| H | -0.34595900 | 0.76216100 | -3.07629600 |
| H | -1.77910000 | -0.27953400 | -2.82212600 |
| H | -0.13302600 | -0.93211900 | -2.54387700 |

**7**

E (M06-SMD/BS1) = -814.7975368 au
H (M06-SMD/BS1) = -814.558288 au
G (M06-SMD/BS1) = -814.616601 au
E (M06-SMD/BS2//M06-SMD/BS1) = -815.0853943 au

| | | | |
|---|---|---|---|
| C | -1.46560300 | 2.37471600 | -0.06201600 |
| C | -0.82135600 | 1.14448600 | -0.02562500 |
| C | -1.63567600 | -0.03835200 | 0.01939900 |
| C | -3.05153400 | 0.06769000 | 0.03638000 |
| C | -3.66193800 | 1.34209100 | 0.00319700 |
| C | -2.87111900 | 2.46277800 | -0.04597000 |
| H | -0.87089200 | 3.27995000 | -0.10017900 |
| C | -3.80199900 | -1.13009300 | 0.08421600 |
| H | -4.74913100 | 1.40902900 | 0.01477400 |
| H | -3.32823400 | 3.45101100 | -0.07523800 |
| C | -3.16258500 | -2.34237300 | 0.11254900 |
| C | -1.75638800 | -2.36070000 | 0.09144100 |
| H | -4.89008700 | -1.06713300 | 0.09795100 |
| H | -3.71326900 | -3.27889100 | 0.14974000 |
| H | -1.21508700 | -3.30652800 | 0.11262500 |
| N | -1.02159000 | -1.26475600 | 0.04646300 |
| N | 0.55810100 | 0.94197100 | -0.01955400 |
| C | 1.52468700 | 1.90240200 | 0.06235900 |

| | | | |
|---|---|---|---|
| C | 2.90398200 | 1.29842300 | 0.20488500 |
| H | 3.64730600 | 1.97380800 | -0.24726500 |
| H | 3.11841600 | 1.29177100 | 1.28815100 |
| C | 2.98634900 | -0.12082400 | -0.33664200 |
| H | 3.11308200 | -0.11154200 | -1.43270800 |
| C | 4.00814600 | -1.00307700 | 0.33085700 |
| H | 3.86113400 | -1.03590700 | 1.42082500 |
| H | 3.96974200 | -2.03394400 | -0.05234100 |
| H | 5.03520000 | -0.63615100 | 0.15306300 |
| O | 1.32803800 | 3.12272600 | 0.07931700 |
| Pd | 1.16243800 | -0.96175600 | -0.10875000 |

**8**

E (M06-SMD/BS1) = -1360.558684 au

H (M06-SMD/BS1) = -1360.169958 au

G (M06-SMD/BS1) = -1360.260445 au

E (M06-SMD/BS2//M06-SMD/BS1) = -1647.42891 au

| | | | |
|---|---|---|---|
| C | 3.75898000 | 0.16628600 | 1.99510400 |
| C | 2.74913500 | 0.05052200 | 1.05412000 |
| C | 2.27230300 | -1.25517600 | 0.71462000 |
| C | 2.80599500 | -2.39439200 | 1.37178200 |
| C | 3.80676000 | -2.23166300 | 2.35591800 |
| C | 4.26883400 | -0.97278200 | 2.64805000 |
| H | 4.14766100 | 1.14805400 | 2.24341800 |
| C | 2.31080800 | -3.66168300 | 0.98528900 |
| H | 4.20383800 | -3.11348900 | 2.85684600 |
| H | 5.04941000 | -0.83614500 | 3.39446200 |
| C | 1.36001200 | -3.75896500 | 0.00166500 |
| C | 0.89967000 | -2.57707800 | -0.60878300 |
| H | 2.70413200 | -4.55108900 | 1.47712700 |
| H | 0.96597000 | -4.71995100 | -0.31875900 |
| H | 0.15939100 | -2.61767900 | -1.40927000 |
| N | 1.33236900 | -1.38098400 | -0.26699500 |
| N | 2.18060500 | 1.14804400 | 0.39950500 |
| C | 2.03671800 | 2.42261700 | 0.88218700 |
| C | 1.23334500 | 3.31079600 | -0.03437000 |
| H | 1.66616500 | 4.32278900 | -0.02339200 |
| H | 0.22079800 | 3.39569800 | 0.39141400 |
| C | 1.18656600 | 2.77352800 | -1.44158600 |
| H | 2.16171700 | 2.82865500 | -1.93982300 |
| C | 0.05407200 | 3.19715600 | -2.31430100 |

| | | | |
|---|---|---|---|
| H | -0.91783000 | 3.10448700 | -1.81529400 |
| H | 0.03494100 | 2.63578500 | -3.25875700 |
| H | 0.19697300 | 4.25955800 | -2.57766200 |
| O | 2.49048200 | 2.78775500 | 1.96215400 |
| Pd | 1.06075400 | 0.71285000 | -1.16575500 |
| C | -3.08494400 | 1.76067700 | 2.06942800 |
| C | -2.62290300 | 0.81423700 | 1.14841800 |
| C | -3.55629000 | -0.01217200 | 0.52097600 |
| C | -4.91617300 | 0.10689000 | 0.79271900 |
| C | -5.35737700 | 1.07625200 | 1.68873900 |
| C | -4.44088100 | 1.90383700 | 2.33024300 |
| H | -2.35456700 | 2.39294700 | 2.57400100 |
| H | -5.63188200 | -0.55597700 | 0.30991200 |
| H | -6.42301700 | 1.17326300 | 1.88975000 |
| H | -4.78142400 | 2.65580200 | 3.03955900 |
| I | -2.96269400 | -1.59275600 | -0.80066300 |
| C | -1.13605800 | 0.76457900 | 0.92176400 |
| O | -0.37997200 | 0.62730300 | 1.88269500 |
| O | -0.80251900 | 0.93885900 | -0.31284000 |
| O | 2.65014300 | 0.62251700 | -2.34280400 |
| C | 3.14499800 | -0.65930400 | -2.60164500 |
| H | 3.65268000 | -1.11034700 | -1.73081500 |
| H | 3.90127300 | -0.55390800 | -3.39816700 |
| H | 2.37458100 | -1.35932800 | -2.96793900 |

**$TS_1$**

E (M06-SMD/BS1) = -1360.520576 au

H (M06-SMD/BS1) = -1360.134626 au

G (M06-SMD/BS1) = -1360.222711 au

E (M06-SMD/BS2//M06-SMD/BS1) = -1647.391502 au

| | | | |
|---|---|---|---|
| C | 3.86588600 | 0.73956700 | -1.65876000 |
| C | 2.69300300 | 0.59685000 | -0.92710000 |
| C | 2.05357100 | 1.78790400 | -0.43714100 |
| C | 2.59761400 | 3.06004700 | -0.78341400 |
| C | 3.75913000 | 3.15272600 | -1.58030500 |
| C | 4.38218000 | 2.00264200 | -1.99449500 |
| H | 4.38680400 | -0.14839400 | -2.00141900 |
| C | 1.94484100 | 4.21307300 | -0.28779000 |
| H | 4.14728900 | 4.13750000 | -1.83869300 |
| H | 5.29208900 | 2.05306200 | -2.59048900 |
| C | 0.84269000 | 4.08377900 | 0.51456500 |

| | | | |
|---|---|---|---|
| C | 0.39752000 | 2.78304100 | 0.82462200 |
| H | 2.34289000 | 5.19348900 | -0.55025700 |
| H | 0.32088600 | 4.94909200 | 0.91661200 |
| H | -0.46887000 | 2.64833200 | 1.47676000 |
| N | 0.96573200 | 1.68530000 | 0.37299800 |
| N | 2.18084500 | -0.66436800 | -0.60324900 |
| C | 2.43797400 | -1.79870400 | -1.30575600 |
| C | 1.98342300 | -3.06425300 | -0.59278400 |
| H | 2.50801000 | -3.91557700 | -1.06512300 |
| H | 0.90388400 | -3.21696500 | -0.73720900 |
| C | 2.36226300 | -3.14484200 | 0.84145800 |
| H | 3.42939100 | -3.05275700 | 1.04177300 |
| C | 1.57043500 | -3.94907000 | 1.77211400 |
| H | 0.49046200 | -3.83173900 | 1.62214700 |
| H | 1.85018900 | -3.78607900 | 2.81659300 |
| H | 1.82224600 | -4.99936700 | 1.52638500 |
| O | 2.95581800 | -1.85256100 | -2.42161400 |
| Pd | 0.95886600 | -0.91739300 | 0.97505200 |
| C | -3.18022300 | -1.36015000 | -2.48575400 |
| C | -2.74848200 | -0.69076300 | -1.33464000 |
| C | -3.71748500 | -0.14268200 | -0.49079000 |
| C | -5.07409100 | -0.26784800 | -0.77816400 |
| C | -5.47934400 | -0.96657700 | -1.91137000 |
| C | -4.53014700 | -1.51291000 | -2.76937000 |
| H | -2.42580600 | -1.77030100 | -3.15621000 |
| H | -5.81682200 | 0.18037100 | -0.12063700 |
| H | -6.54204600 | -1.07185100 | -2.12372200 |
| H | -4.84050900 | -2.05148200 | -3.66288800 |
| I | -3.21417400 | 1.03402700 | 1.23131600 |
| C | -1.25922600 | -0.62437600 | -1.11575000 |
| O | -0.52912200 | -0.30760900 | -2.05842900 |
| O | -0.88934600 | -0.96705100 | 0.06620100 |
| O | 2.54065500 | -1.30438600 | 2.11486100 |
| C | 3.74961500 | -0.61771100 | 1.93691100 |
| H | 4.30879300 | -0.95610300 | 1.04725000 |
| H | 4.37762800 | -0.80804400 | 2.82150500 |
| H | 3.59994800 | 0.47133200 | 1.85137300 |

**$TS_2$**

E (M06-SMD/BS1) = -1360.522974 au

H (M06-SMD/BS1) = -1360.138011 au

G (M06-SMD/BS1) = -1360.228243 au
E (M06-SMD/BS2//M06-SMD/BS1) = -1647.395626 au

| | | | |
|---|---|---|---|
| C | 4.33070800 | 1.29631000 | -1.06890500 |
| C | 3.30140500 | 0.78378700 | -0.30073500 |
| C | 3.62111300 | -0.03419200 | 0.82851600 |
| C | 4.99202700 | -0.27084400 | 1.13894800 |
| C | 6.01267900 | 0.29751700 | 0.34322700 |
| C | 5.68072400 | 1.06686100 | -0.74423800 |
| H | 4.08253000 | 1.90184000 | -1.93954400 |
| C | 5.28075300 | -1.08549700 | 2.25853100 |
| H | 7.05306300 | 0.10753500 | 0.60662300 |
| H | 6.45860100 | 1.50127200 | -1.36996400 |
| C | 4.25463200 | -1.61682600 | 2.99427600 |
| C | 2.93124000 | -1.32952200 | 2.59879600 |
| H | 6.32264000 | -1.27770900 | 2.51588900 |
| H | 4.43871000 | -2.24721700 | 3.86126300 |
| H | 2.09890200 | -1.74721100 | 3.16947400 |
| N | 2.61414100 | -0.57560800 | 1.56731700 |
| N | 1.95783700 | 1.02251200 | -0.65591400 |
| C | 1.53682200 | 2.29312700 | -0.79398200 |
| C | 0.14573200 | 2.51999900 | -1.37169800 |
| H | 0.25618900 | 3.33859200 | -2.11746700 |
| H | -0.53231400 | 2.93596900 | -0.61193100 |
| C | -0.48603400 | 1.48538400 | -2.17091400 |
| H | 0.16148700 | 0.84289600 | -2.77746100 |
| C | -1.89437100 | 1.49278800 | -2.51016000 |
| H | -2.52310800 | 2.02400800 | -1.78909900 |
| H | -2.26537900 | 0.48422500 | -2.72811800 |
| H | -1.92758500 | 2.02974200 | -3.48058700 |
| O | 2.17982300 | 3.30074500 | -0.47180700 |
| Pd | 0.79396600 | -0.65537400 | -0.84651200 |
| C | -2.93984200 | 2.33213300 | 1.66458000 |
| C | -2.70345300 | 1.08165000 | 1.07935200 |
| C | -3.80860700 | 0.30954600 | 0.70420800 |
| C | -5.10486700 | 0.77512300 | 0.90624100 |
| C | -5.31313300 | 2.03310100 | 1.46454200 |
| C | -4.22811300 | 2.81493900 | 1.84577200 |
| H | -2.07990400 | 2.92837200 | 1.96713300 |
| H | -5.95723200 | 0.15775300 | 0.62877800 |
| H | -6.33087600 | 2.39317500 | 1.60562300 |
| H | -4.38377000 | 3.79665600 | 2.28896500 |

| | | | |
|---|---|---|---|
| I | -3.64200200 | -1.67429800 | -0.09858000 |
| C | -1.26101400 | 0.71066500 | 0.87985100 |
| O | -0.42768300 | 0.99752800 | 1.73996800 |
| O | -1.01736300 | 0.14838800 | -0.25774900 |
| O | 2.19320800 | -1.83005300 | -1.63632600 |
| C | 1.24500000 | -2.80948100 | -1.72764800 |
| H | 1.45546700 | -3.70108800 | -1.11395200 |
| H | 0.96680700 | -3.08340400 | -2.75877700 |
| H | 0.22127900 | -2.44103800 | -1.27640700 |

**9a**

E (M06-SMD/BS1) = -1044.858926 au
H (M06-SMD/BS1) = -1044.52923 au
G (M06-SMD/BS1) = -1044.601712 au
E (M06-SMD/BS2//M06-SMD/BS1) = -1045.237777 au

| | | | |
|---|---|---|---|
| C | -1.65432200 | 2.41914100 | -0.57714700 |
| C | -1.00355400 | 1.24886200 | -0.21921100 |
| C | -1.79322400 | 0.09887600 | 0.10631800 |
| C | -3.21008600 | 0.18388600 | 0.08368800 |
| C | -3.83484500 | 1.40302500 | -0.26204500 |
| C | -3.06092600 | 2.48890600 | -0.58647200 |
| H | -1.07090400 | 3.29425100 | -0.84093400 |
| C | -3.93914100 | -0.98517100 | 0.40478200 |
| H | -4.92268500 | 1.45614300 | -0.27246700 |
| H | -3.53035300 | 3.43130700 | -0.86370100 |
| C | -3.27821400 | -2.14345500 | 0.72401900 |
| C | -1.87048400 | -2.13954800 | 0.71239700 |
| H | -5.02815500 | -0.94441100 | 0.39073100 |
| H | -3.81341400 | -3.05541300 | 0.97614400 |
| H | -1.31113500 | -3.04610800 | 0.94725400 |
| N | -1.16154400 | -1.07123500 | 0.41336800 |
| N | 0.38846200 | 1.11419500 | -0.16996500 |
| C | 1.32820300 | 2.10496300 | -0.05462300 |
| C | 2.73557300 | 1.56625300 | 0.02333500 |
| H | 3.41557500 | 2.26631200 | -0.48503400 |
| H | 3.02770200 | 1.55491100 | 1.08516800 |
| C | 2.84011100 | 0.18784200 | -0.57795300 |
| H | 2.71498600 | 0.20509300 | -1.66763900 |
| C | 3.96649900 | -0.68466200 | -0.13342000 |
| H | 4.05166100 | -0.73379800 | 0.95888600 |
| H | 3.87516300 | -1.70305400 | -0.53665900 |

| | | | |
|---|---|---|---|
| H | 4.91152800 | -0.27000600 | -0.52508100 |
| O | 1.05060900 | 3.29946100 | 0.02192300 |
| Pd | 1.06463500 | -0.73447800 | -0.04707700 |
| O | 1.54667900 | -0.61398200 | 1.89832800 |
| O | 0.83360900 | -1.08502700 | -2.01591900 |
| C | -0.22439200 | -1.93063900 | -2.35645900 |
| H | -1.21505000 | -1.44512700 | -2.27446400 |
| H | -0.09887100 | -2.22776800 | -3.41247100 |
| H | -0.24935200 | -2.86207800 | -1.76097300 |
| C | 0.76245000 | 0.13549300 | 2.77284800 |
| H | 0.82538200 | 1.22549600 | 2.59138800 |
| H | -0.30513100 | -0.15013600 | 2.75823100 |
| H | 1.13235600 | -0.03659500 | 3.79834800 |

**$TS_3$**

E (M06-SMD/BS1) = -1044.824066 au
H (M06-SMD/BS1) = -1044.496194 au
G (M06-SMD/BS1) = -1044.568681 au
E (M06-SMD/BS2//M06-SMD/BS1) = -1045.204914 au

| | | | |
|---|---|---|---|
| C | -1.51857100 | 2.21461900 | 0.16493900 |
| C | -1.05318700 | 0.98325800 | -0.25891600 |
| C | -1.98506700 | -0.07651200 | -0.48911700 |
| C | -3.37349800 | 0.18033600 | -0.29017100 |
| C | -3.80841400 | 1.45955800 | 0.12484500 |
| C | -2.89161100 | 2.45825800 | 0.34797700 |
| H | -0.79646200 | 3.00788700 | 0.35491100 |
| C | -4.27733300 | -0.88432600 | -0.51325000 |
| H | -4.87522700 | 1.63078700 | 0.26839300 |
| H | -3.21930100 | 3.44366800 | 0.67446500 |
| C | -3.79958500 | -2.10781200 | -0.90274300 |
| C | -2.40662600 | -2.25926200 | -1.06797200 |
| H | -5.34376900 | -0.71076900 | -0.36749900 |
| H | -4.46334100 | -2.95037000 | -1.08243400 |
| H | -2.00704800 | -3.22741400 | -1.37841200 |
| N | -1.52717800 | -1.29861100 | -0.87458100 |
| N | 0.32538500 | 0.75575300 | -0.41783600 |
| C | 1.05210000 | 1.51887800 | -1.26308100 |
| C | 2.55342800 | 1.29363000 | -1.27481800 |
| H | 3.02623900 | 2.29131700 | -1.34930000 |
| H | 2.83057700 | 0.77041300 | -2.20113100 |
| C | 3.17372400 | 0.67853700 | -0.09487600 |

| | | | |
|---|---|---|---|
| H | 2.89992500 | 1.11087800 | 0.87394000 |
| C | 4.50869200 | 0.08226900 | -0.15348300 |
| H | 4.72537800 | -0.40333700 | -1.10996200 |
| H | 4.68825500 | -0.60064200 | 0.68443300 |
| H | 5.20947800 | 0.92776800 | -0.01577200 |
| O | 0.56654400 | 2.32683000 | -2.06336700 |
| Pd | 1.18337000 | -0.53692900 | 0.86664200 |
| O | 2.34138300 | -1.47141800 | -0.49311900 |
| O | 0.33133900 | 0.30765600 | 2.46357700 |
| C | -0.85435100 | -0.32225500 | 2.83886800 |
| H | -1.70669400 | -0.06713700 | 2.17885700 |
| H | -1.13386500 | 0.00578500 | 3.85600400 |
| H | -0.76843200 | -1.42638500 | 2.87083900 |
| C | 1.93448500 | -1.73975100 | -1.79982400 |
| H | 1.30836400 | -0.94657900 | -2.24170000 |
| H | 1.34541900 | -2.67345800 | -1.85118300 |
| H | 2.81881300 | -1.86474100 | -2.45002300 |

**$TS_{17\text{-}18}$**

E (M06-SMD/BS1) = -1360.532226 au
H (M06-SMD/BS1) = -1360.144803 au
G (M06-SMD/BS1) = -1360.232178 au
E (M06-SMD/BS2//M06-SMD/BS1) = -1647.424154 au

| | | | |
|---|---|---|---|
| C | 4.66813400 | 0.64764000 | 1.18624300 |
| C | 3.47754900 | 0.40483300 | 0.51260700 |
| C | 3.30612800 | -0.87719300 | -0.10527300 |
| C | 4.32233700 | -1.86463100 | -0.00086200 |
| C | 5.51670800 | -1.57255500 | 0.69530200 |
| C | 5.67265000 | -0.33575300 | 1.26900800 |
| H | 4.82077400 | 1.61173600 | 1.65678400 |
| C | 4.08638400 | -3.12046000 | -0.60494200 |
| H | 6.28986800 | -2.33685400 | 0.76347300 |
| H | 6.58906100 | -0.09631200 | 1.80669900 |
| C | 2.90585200 | -3.35452100 | -1.26143200 |
| C | 1.95695000 | -2.31928400 | -1.32773900 |
| H | 4.85412900 | -3.89090700 | -0.53342200 |
| H | 2.69060200 | -4.31134100 | -1.73035600 |
| H | 1.01156500 | -2.47812700 | -1.84534100 |
| N | 2.14421900 | -1.12960500 | -0.78611600 |
| N | 2.41387000 | 1.29571400 | 0.37797500 |
| C | 2.29996400 | 2.50065400 | 1.00279200 |

| | | | |
|---|---|---|---|
| C | 0.95316200 | 3.13417200 | 0.74956800 |
| H | 1.04502500 | 4.23099000 | 0.81383100 |
| H | 0.29504600 | 2.83503300 | 1.58511600 |
| C | 0.33251700 | 2.70977100 | -0.56502500 |
| H | 0.93527200 | 3.07081300 | -1.41696100 |
| C | -1.10673400 | 3.11916600 | -0.70978100 |
| H | -1.73944400 | 2.69767600 | 0.08500700 |
| H | -1.52948100 | 2.83583900 | -1.68049800 |
| H | -1.17875000 | 4.21771500 | -0.62767500 |
| O | 3.14936700 | 3.03230100 | 1.72770800 |
| Pd | 0.85049200 | 0.72315300 | -0.77029900 |
| C | -5.59917500 | -0.54951500 | 0.39852900 |
| C | -4.25067900 | -0.67257000 | 0.73397700 |
| C | -3.30778900 | -0.15209400 | -0.14449300 |
| C | -3.64597700 | 0.48207100 | -1.32398700 |
| C | -5.00262200 | 0.59322000 | -1.63267000 |
| C | -5.97540200 | 0.08200800 | -0.77849300 |
| H | -6.33805800 | -0.96173600 | 1.08428300 |
| H | -2.88125300 | 0.87502400 | -1.98636200 |
| H | -5.29171400 | 1.08961200 | -2.55750200 |
| H | -7.02912400 | 0.17654400 | -1.03274700 |
| I | -1.27245700 | -0.40988000 | 0.57125700 |
| C | -3.84606100 | -1.35322100 | 1.99591500 |
| O | -4.67457300 | -1.82227100 | 2.77405000 |
| O | -2.56562100 | -1.41526400 | 2.20806000 |
| O | -0.82798800 | 0.31271100 | -1.89326500 |
| C | -0.81272400 | -0.80135500 | -2.74199900 |
| H | -1.59737300 | -0.66331900 | -3.50591100 |
| H | -1.04306200 | -1.74638400 | -2.21696900 |
| H | 0.15134100 | -0.92224000 | -3.26321700 |

**18**

E (M06-SMD/BS1) = -1360.547193 au

H (M06-SMD/BS1) = -1360.159447 au

G (M06-SMD/BS1) = -1360.248961 au

E (M06-SMD/BS2//M06-SMD/BS1) = -1647.433157 au

| | | | |
|---|---|---|---|
| C | 3.65786800 | -1.63387700 | 1.24921200 |
| C | 2.84587200 | -0.67281500 | 0.66330700 |
| C | 2.99362300 | -0.43279900 | -0.74044900 |
| C | 3.96719400 | -1.13716500 | -1.49383400 |
| C | 4.78469800 | -2.09540300 | -0.85211900 |

| | | | |
|---|---|---|---|
| C | 4.61804400 | -2.33183300 | 0.48990400 |
| H | 3.55880700 | -1.83879600 | 2.30918100 |
| C | 4.06453600 | -0.84582800 | -2.87371500 |
| H | 5.52825800 | -2.63429400 | -1.43775700 |
| H | 5.23664200 | -3.07319100 | 0.99296600 |
| C | 3.23228100 | 0.08499600 | -3.44437700 |
| C | 2.28738000 | 0.73167800 | -2.62912000 |
| H | 4.80748200 | -1.37426500 | -3.47078300 |
| H | 3.28578500 | 0.32566600 | -4.50293100 |
| H | 1.60052100 | 1.47128000 | -3.04342800 |
| N | 2.17410200 | 0.48343800 | -1.33840000 |
| N | 1.87611300 | 0.09095000 | 1.32221100 |
| C | 1.66825200 | 0.14264900 | 2.67008900 |
| C | 0.42438800 | 0.92291400 | 3.03484900 |
| H | 0.60433000 | 1.44673700 | 3.98765900 |
| H | -0.37267400 | 0.18955900 | 3.24065800 |
| C | -0.02692200 | 1.91296700 | 1.99246100 |
| H | 0.67596800 | 2.76351400 | 1.89906700 |
| C | -1.45188900 | 2.35279800 | 2.08363000 |
| H | -2.14954600 | 1.50530300 | 2.05222600 |
| H | -1.72077700 | 3.07037200 | 1.29950200 |
| H | -1.59685400 | 2.85722900 | 3.05534100 |
| O | 2.37872700 | -0.38597800 | 3.52761700 |
| Pd | 0.67938800 | 1.22375900 | 0.14920100 |
| C | -5.17591100 | -0.65349700 | -0.90278300 |
| C | -3.91693200 | -1.08529100 | -0.48285600 |
| C | -2.89017600 | -0.14771300 | -0.46258900 |
| C | -3.05720300 | 1.17201300 | -0.84490700 |
| C | -4.32833400 | 1.57002700 | -1.26083600 |
| C | -5.38460100 | 0.66421500 | -1.28661100 |
| H | -5.98420400 | -1.38306500 | -0.92254200 |
| H | -2.22347000 | 1.87314400 | -0.84059700 |
| H | -4.48342200 | 2.60286400 | -1.56878000 |
| H | -6.37200300 | 0.98581600 | -1.61233100 |
| I | -0.98081300 | -0.87979500 | 0.22093600 |
| C | -3.70303700 | -2.51748200 | -0.09018100 |
| O | -4.64988500 | -3.31405700 | -0.11018100 |
| O | -2.50390400 | -2.82592300 | 0.24519200 |
| O | -0.28260000 | 2.47571600 | -1.09988100 |
| C | 0.45835600 | 3.65410900 | -1.03551800 |
| H | 0.18780500 | 4.28909400 | -0.17001000 |

| | | | |
|---|---|---|---|
| H | 0.30024800 | 4.24782600 | -1.95337600 |
| H | 1.55321000 | 3.46469600 | -0.97688400 |

**$TS_{18-19}$**

E (M06-SMD/BS1) = -1360.544627 au

H (M06-SMD/BS1) = -1360.157707 au

G (M06-SMD/BS1) = -1360.244617 au

E (M06-SMD/BS2//M06-SMD/BS1) = -1647.430003 au

| | | | |
|---|---|---|---|
| C | -3.68269800 | -2.29724900 | -0.05417200 |
| C | -2.95512600 | -1.12357600 | -0.16275600 |
| C | -3.41737700 | 0.02440600 | 0.54382100 |
| C | -4.61290300 | -0.02290700 | 1.30353800 |
| C | -5.33710000 | -1.23532200 | 1.37348900 |
| C | -4.86766800 | -2.34150700 | 0.70922400 |
| H | -3.33987800 | -3.18977200 | -0.56633600 |
| C | -5.00783200 | 1.15685000 | 1.97309800 |
| H | -6.24929400 | -1.27247000 | 1.96726400 |
| H | -5.41163300 | -3.28265100 | 0.76839300 |
| C | -4.24005100 | 2.29221800 | 1.88279600 |
| C | -3.06424100 | 2.25513600 | 1.11484800 |
| H | -5.92444100 | 1.14657500 | 2.56224100 |
| H | -4.51760500 | 3.21082200 | 2.39294500 |
| H | -2.42333200 | 3.13277600 | 1.02834300 |
| N | -2.67378700 | 1.16947500 | 0.47295300 |
| N | -1.78958500 | -0.94064200 | -0.91823200 |
| C | -1.50242000 | -1.64374800 | -2.04689200 |
| C | -0.27339500 | -1.11809400 | -2.75142600 |
| H | -0.42281300 | -1.22940100 | -3.83877900 |
| H | 0.59063800 | -1.75026300 | -2.49659400 |
| C | 0.00516700 | 0.32307700 | -2.47224100 |
| H | -0.80743900 | 0.99878900 | -2.78624600 |
| O | -2.14912500 | -2.59570900 | -2.48849600 |
| Pd | -0.72034100 | 0.73499800 | -0.45332100 |
| C | 5.45882100 | 0.31439700 | 0.70825600 |
| C | 4.25458300 | -0.39098400 | 0.77036700 |
| C | 3.09696400 | 0.29554300 | 0.41608700 |
| C | 3.09212800 | 1.62874200 | 0.03884300 |
| C | 4.31146400 | 2.30349000 | -0.00628200 |
| C | 5.49380300 | 1.64662200 | 0.31987000 |
| H | 6.36935200 | -0.21593900 | 0.98254700 |
| H | 2.15767400 | 2.14073400 | -0.18783600 |

| | | | |
|---|---|---|---|
| H | 4.32610300 | 3.35282300 | -0.29666600 |
| H | 6.44323800 | 2.17748300 | 0.28140900 |
| I | 1.24855900 | -0.81896600 | 0.46300600 |
| C | 4.25561600 | -1.82388700 | 1.24143600 |
| O | 5.34237400 | -2.37936100 | 1.47133000 |
| O | 3.10962800 | -2.36202600 | 1.38080800 |
| O | 0.08323600 | 2.48444900 | 0.09610400 |
| C | -0.29819300 | 3.48266900 | -0.80103700 |
| H | 0.26936200 | 3.44763400 | -1.75156600 |
| H | -0.11409900 | 4.47002900 | -0.34313400 |
| H | -1.37617500 | 3.44674200 | -1.05942600 |
| C | 1.36155700 | 0.83129200 | -2.79033700 |
| H | 1.43034400 | 0.85050500 | -3.89426400 |
| H | 2.16197600 | 0.17785000 | -2.42389200 |
| H | 1.52820600 | 1.85937100 | -2.44916000 |

**19**

E (M06-SMD/BS1) = -1360.55137 au

H (M06-SMD/BS1) = -1360.163366 au

G (M06-SMD/BS1) = -1360.251601 au

E (M06-SMD/BS2//M06-SMD/BS1) = -1647.433692 au

| | | | |
|---|---|---|---|
| C | -4.25458000 | -1.17578700 | 1.25862600 |
| C | -3.19585300 | -0.62299200 | 0.56810800 |
| C | -3.32439200 | 0.69864500 | 0.06658900 |
| C | -4.52448600 | 1.43022300 | 0.22435500 |
| C | -5.59156200 | 0.83070600 | 0.93477800 |
| C | -5.44550300 | -0.43801900 | 1.44026300 |
| H | -4.17220200 | -2.18214700 | 1.65878500 |
| C | -4.57694500 | 2.72206800 | -0.34541500 |
| H | -6.51372700 | 1.39288800 | 1.07329500 |
| H | -6.26279200 | -0.89808000 | 1.99269800 |
| C | -3.48989300 | 3.22324100 | -1.01987700 |
| C | -2.32137100 | 2.44884500 | -1.11308100 |
| H | -5.48889000 | 3.30909300 | -0.24184300 |
| H | -3.50454600 | 4.21174300 | -1.47057500 |
| H | -1.43207900 | 2.82925600 | -1.61208400 |
| N | -2.24955700 | 1.23751100 | -0.59121000 |
| N | -1.98757300 | -1.25361000 | 0.22547800 |
| C | -2.01320100 | -2.46452700 | -0.40267800 |
| C | -0.82852100 | -2.62316300 | -1.32682500 |
| H | -1.09878700 | -3.31880500 | -2.13972600 |

| | | | |
|---|---|---|---|
| H | 0.03785900 | -3.06272400 | -0.81262000 |
| C | -0.49248900 | -1.31923300 | -1.96035300 |
| H | -1.33694700 | -0.79683200 | -2.42255700 |
| O | -2.90590200 | -3.30273900 | -0.29929400 |
| Pd | -0.52464000 | 0.14097100 | -0.17820300 |
| C | 4.77596000 | 1.46621800 | -0.11517300 |
| C | 3.87959800 | 0.39192800 | -0.07223200 |
| C | 3.02835400 | 0.33441500 | 1.02984000 |
| C | 3.05335800 | 1.27730400 | 2.05364300 |
| C | 3.98242600 | 2.31136800 | 1.99627100 |
| C | 4.84289600 | 2.40509300 | 0.90613900 |
| H | 5.43260300 | 1.54732600 | -0.98048600 |
| H | 2.34788400 | 1.21530800 | 2.88042600 |
| H | 4.01850900 | 3.04723000 | 2.79765500 |
| H | 5.56321100 | 3.21936600 | 0.84673000 |
| I | 1.47347600 | -1.13437100 | 1.17422600 |
| C | 3.89826200 | -0.59761000 | -1.22930000 |
| O | 4.03100800 | -0.09845600 | -2.37198600 |
| O | 3.79322400 | -1.81487700 | -0.94323200 |
| O | 0.65424100 | 1.74600300 | -0.29661700 |
| C | 1.15833200 | 2.12091900 | -1.53740300 |
| H | 1.99793700 | 1.48277600 | -1.87384500 |
| H | 1.54827100 | 3.15184900 | -1.46030500 |
| H | 0.39477900 | 2.12809300 | -2.34207200 |
| C | 0.80903600 | -1.14635300 | -2.61509500 |
| H | 1.64039700 | -1.60208500 | -2.06532200 |
| H | 1.02745800 | -0.10902300 | -2.88897300 |
| H | 0.70304400 | -1.69473000 | -3.57378200 |

**20**

E (M06-SMD/BS1) = -1360.560484 au
H (M06-SMD/BS1) = -1360.171973 au
G (M06-SMD/BS1) = -1360.25975 au
E (M06-SMD/BS2//M06-SMD/BS1) = -1647.436952 au

| | | | |
|---|---|---|---|
| C | -3.28335600 | 0.86911600 | 2.17368300 |
| C | -2.51834000 | 0.36922400 | 1.14245300 |
| C | -2.83124500 | 0.75843700 | -0.18424600 |
| C | -3.92617300 | 1.60468000 | -0.47092300 |
| C | -4.69430000 | 2.09456500 | 0.61240200 |
| C | -4.36663800 | 1.73490000 | 1.89687400 |
| H | -3.05600500 | 0.59178500 | 3.19950100 |

| | | | |
|---|---|---|---|
| C | -4.17788800 | 1.90105400 | -1.82947900 |
| H | -5.53354300 | 2.75698000 | 0.40614900 |
| H | -4.95261800 | 2.11680900 | 2.73092200 |
| C | -3.37245700 | 1.37900900 | -2.81333300 |
| C | -2.28171000 | 0.56958800 | -2.45307900 |
| H | -5.01696000 | 2.54804200 | -2.08294900 |
| H | -3.54635000 | 1.59060400 | -3.86479500 |
| H | -1.59243800 | 0.17099100 | -3.19544400 |
| N | -2.03804800 | 0.27801000 | -1.18935300 |
| N | -1.48235500 | -0.58173200 | 1.23990800 |
| C | -1.79666000 | -1.81241600 | 1.75400400 |
| C | -1.08237900 | -2.91264700 | 1.01449900 |
| H | -1.65075800 | -3.84968500 | 1.12742700 |
| H | -0.08824600 | -3.10151400 | 1.44872100 |
| C | -0.99530800 | -2.57340200 | -0.45241300 |
| H | -1.97896900 | -2.37301600 | -0.89498500 |
| O | -2.61432600 | -2.01113900 | 2.64958600 |
| Pd | -0.29118500 | -0.51753700 | -0.44401500 |
| C | 3.28406700 | 3.06096800 | 0.30441300 |
| C | 2.51300200 | 1.90778400 | 0.48419300 |
| C | 3.12314600 | 0.68627000 | 0.21334000 |
| C | 4.43365000 | 0.58546600 | -0.24048800 |
| C | 5.16764300 | 1.75236400 | -0.43501200 |
| C | 4.59174600 | 2.98881600 | -0.15931500 |
| H | 2.83021100 | 4.02636200 | 0.52848400 |
| H | 4.88165300 | -0.38731700 | -0.43521200 |
| H | 6.19202500 | 1.68659300 | -0.79687300 |
| H | 5.16567900 | 3.90271400 | -0.30192200 |
| I | 2.12545700 | -1.16577500 | 0.63730800 |
| C | 1.07716700 | 2.08863000 | 0.92412400 |
| O | 0.87680200 | 2.60337100 | 2.03624500 |
| O | 0.18742100 | 1.75524700 | 0.07829000 |
| O | 0.71064600 | -0.09157200 | -2.15201600 |
| C | 0.87742600 | -1.03609300 | -3.15334900 |
| H | 1.54346200 | -1.87182600 | -2.86595500 |
| H | 1.34569800 | -0.53631700 | -4.02045300 |
| H | -0.07716200 | -1.46749700 | -3.51777900 |
| C | -0.15395200 | -3.48821000 | -1.27099400 |
| H | 0.87576800 | -3.57109900 | -0.90120700 |
| H | -0.14934200 | -3.24052100 | -2.33504000 |
| H | -0.61300200 | -4.48913800 | -1.17609800 |

**$TS_4$**

E (M06-SMD/BS1) = -1360.515903 au
H (M06-SMD/BS1) = -1360.129131 au
G (M06-SMD/BS1) = -1360.217926 au
E (M06-SMD/BS2//M06-SMD/BS1) = -1647.398345 au

| | | | |
|---|---|---|---|
| C | -3.98047400 | -1.03590900 | 1.90568900 |
| C | -3.03337000 | -0.57797000 | 1.02086700 |
| C | -3.22304400 | 0.67827700 | 0.39598300 |
| C | -4.38003600 | 1.45178900 | 0.64100900 |
| C | -5.33364600 | 0.95509700 | 1.56264200 |
| C | -5.12815000 | -0.25627600 | 2.17839400 |
| H | -3.84550300 | -2.00424300 | 2.38456000 |
| C | -4.49615700 | 2.67952700 | -0.05019200 |
| H | -6.21950900 | 1.55154800 | 1.77603000 |
| H | -5.86225800 | -0.63433900 | 2.88741600 |
| C | -3.51088600 | 3.07232800 | -0.92423800 |
| C | -2.38220800 | 2.25254100 | -1.10657200 |
| H | -5.37357100 | 3.30291000 | 0.11888700 |
| H | -3.57779000 | 4.00790800 | -1.47283300 |
| H | -1.57593800 | 2.53581300 | -1.78219300 |
| N | -2.24300600 | 1.10937900 | -0.46021100 |
| N | -1.87970100 | -1.28703400 | 0.59802000 |
| C | -2.11163000 | -2.24583200 | -0.36351400 |
| C | -0.94454500 | -2.53731900 | -1.31213100 |
| H | -1.22454300 | -3.42695500 | -1.91367300 |
| H | -0.03115900 | -2.81869700 | -0.77651300 |
| C | -0.66813100 | -1.52878700 | -2.38671400 |
| O | -3.18244800 | -2.83160800 | -0.51042500 |
| Pd | -0.50969100 | -0.02450500 | -0.24554500 |
| C | 4.72853500 | 1.97396300 | 0.08210200 |
| C | 4.11681700 | 0.74162400 | 0.33920500 |
| C | 2.77977900 | 0.77353600 | 0.72483500 |
| C | 2.06725300 | 1.95852600 | 0.87779000 |
| C | 2.71276600 | 3.16858800 | 0.63720100 |
| C | 4.04577700 | 3.17379300 | 0.23576000 |
| H | 5.76847700 | 1.96717900 | -0.24088600 |
| H | 1.01615100 | 1.93867600 | 1.16708100 |
| H | 2.16710800 | 4.10239700 | 0.76238900 |
| H | 4.55417400 | 4.11643300 | 0.04000700 |
| I | 1.67092400 | -1.04906700 | 0.98115100 |

| | | | |
|---|---|---|---|
| C | 4.93910100 | -0.52973500 | 0.18944700 |
| O | 5.83435500 | -0.51564500 | -0.68472300 |
| O | 4.65588300 | -1.47279900 | 0.97155200 |
| O | 0.33146400 | 0.47072100 | -2.06767900 |
| C | -1.78137500 | -0.97841600 | -3.16940600 |
| H | -2.64156700 | -0.67648600 | -2.56031500 |
| H | -2.11831700 | -1.81739000 | -3.80719300 |
| H | -1.46803400 | -0.16714000 | -3.83090100 |
| H | 0.27781900 | -1.68446600 | -2.90600000 |
| C | 1.68152500 | 0.37616700 | -2.41321500 |
| H | 2.18528600 | -0.51142400 | -1.98902700 |
| H | 2.23906900 | 1.26694800 | -2.07459200 |
| H | 1.77443600 | 0.32425200 | -3.51226800 |

**$TS_5$**

E (M06-SMD/BS1) = -1360.523083 au

H (M06-SMD/BS1) = -1360.136401 au

G (M06-SMD/BS1) = -1360.223392 au

E (M06-SMD/BS2//M06-SMD/BS1) = -1647.39785 au

| | | | |
|---|---|---|---|
| C | -3.75023400 | -0.25514200 | 2.06764100 |
| C | -2.80501700 | -0.20599000 | 1.07182500 |
| C | -2.91693800 | 0.77922600 | 0.06126500 |
| C | -3.99596300 | 1.68976500 | 0.03947600 |
| C | -4.95008800 | 1.61795300 | 1.08376700 |
| C | -4.81987300 | 0.67066100 | 2.07050800 |
| H | -3.67662600 | -1.01624500 | 2.84281200 |
| C | -4.03710600 | 2.61978200 | -1.02410100 |
| H | -5.77556400 | 2.32835400 | 1.09168400 |
| H | -5.55468700 | 0.61971500 | 2.87184800 |
| C | -3.05643300 | 2.60685500 | -1.98672800 |
| C | -2.00517900 | 1.67714700 | -1.89058900 |
| H | -4.85319800 | 3.34032500 | -1.06807400 |
| H | -3.06613600 | 3.30734800 | -2.81744700 |
| H | -1.20136800 | 1.64829900 | -2.62484300 |
| N | -1.93900500 | 0.80729600 | -0.89900400 |
| N | -1.72734800 | -1.11403100 | 0.89624300 |
| C | -2.05739600 | -2.26913700 | 0.22375000 |
| C | -0.94422500 | -2.94952100 | -0.58236100 |
| H | -1.33926300 | -3.94151800 | -0.87934200 |
| H | -0.05559500 | -3.16764300 | 0.02584700 |
| C | -0.56687500 | -2.35162500 | -1.90949000 |

| | | | |
|---|---|---|---|
| H | -1.33421400 | -1.77472800 | -2.42912100 |
| O | -3.18380200 | -2.76440300 | 0.22222800 |
| Pd | -0.28850000 | -0.29785000 | -0.29562900 |
| C | 3.63929100 | 2.65970400 | -0.16576000 |
| C | 2.67784700 | 1.76394100 | 0.32091200 |
| C | 3.12339900 | 0.48364500 | 0.64693600 |
| C | 4.45030100 | 0.09099500 | 0.49745800 |
| C | 5.38436500 | 1.01548000 | 0.04173500 |
| C | 4.97547200 | 2.30447300 | -0.28802200 |
| H | 3.30900400 | 3.65806100 | -0.45031800 |
| H | 4.75512000 | -0.92859100 | 0.72803600 |
| H | 6.42621700 | 0.71871300 | -0.06546800 |
| H | 5.69764900 | 3.03098900 | -0.65649100 |
| I | 1.78208100 | -1.06745900 | 1.28889200 |
| C | 1.24077900 | 2.25835100 | 0.40316100 |
| O | 0.61590800 | 2.04951100 | 1.47327100 |
| O | 0.81850300 | 2.86009100 | -0.61429800 |
| O | 0.58240300 | -0.46979300 | -2.16588900 |
| C | 0.38788800 | -3.11706900 | -2.71947000 |
| H | 1.31322400 | -3.33239700 | -2.16722500 |
| H | 0.60644000 | -2.64519700 | -3.67967600 |
| H | -0.08972200 | -4.09356800 | -2.91299200 |
| C | 1.95909700 | -0.57916000 | -2.37661000 |
| H | 2.43968500 | -1.35590800 | -1.75435100 |
| H | 2.45977300 | 0.37876800 | -2.14802000 |
| H | 2.16169400 | -0.81646800 | -3.43417400 |

**$TS_6$**

E (M06-SMD/BS1) = -1360.551752 au
H (M06-SMD/BS1) = -1360.164386 au
G (M06-SMD/BS1) = -1360.251364 au
E (M06-SMD/BS2//M06-SMD/BS1) = -1647.432426 au

| | | | |
|---|---|---|---|
| C | -4.00154500 | -1.86588700 | 0.68556200 |
| C | -3.01422000 | -0.98598600 | 0.28527700 |
| C | -3.39221200 | 0.34155500 | -0.05144300 |
| C | -4.74818500 | 0.74700100 | -0.03845800 |
| C | -5.73155200 | -0.18733700 | 0.36223500 |
| C | -5.35243900 | -1.45650900 | 0.72409500 |
| H | -3.73821200 | -2.87842400 | 0.97428900 |
| C | -5.03469600 | 2.07803100 | -0.41625600 |
| H | -6.77481600 | 0.12337000 | 0.38893900 |

| | | | |
|---|---|---|---|
| H | -6.10334000 | -2.17450900 | 1.04905800 |
| C | -4.01587100 | 2.92937900 | -0.76789000 |
| C | -2.69101400 | 2.46482200 | -0.72820600 |
| H | -6.07072500 | 2.41499000 | -0.41824300 |
| H | -4.20698600 | 3.95863200 | -1.05920600 |
| H | -1.85308300 | 3.12120800 | -0.95674300 |
| N | -2.39904400 | 1.22137000 | -0.39015200 |
| N | -1.64237700 | -1.25727500 | 0.13065100 |
| C | -1.25407500 | -2.41953200 | -0.44978600 |
| C | 0.19085700 | -2.39131000 | -0.97417800 |
| H | 0.27232400 | -3.21619300 | -1.69590900 |
| H | 0.90027400 | -2.55583700 | -0.15596900 |
| C | 0.43842000 | -1.12866000 | -1.68965900 |
| H | 1.17423500 | -0.40352800 | -1.34765600 |
| O | -1.95565100 | -3.41375200 | -0.61887600 |
| Pd | -0.53790000 | 0.48766700 | 0.15383000 |
| C | 4.58505200 | 0.38202600 | -1.37023900 |
| C | 3.65813500 | -0.26424500 | -0.54381600 |
| C | 3.15476800 | 0.46328300 | 0.53562600 |
| C | 3.56122600 | 1.76494300 | 0.81384500 |
| C | 4.51464900 | 2.36512000 | -0.00389300 |
| C | 5.02257800 | 1.67263600 | -1.09914000 |
| H | 4.97022100 | -0.15890800 | -2.23455600 |
| H | 3.13011300 | 2.31240700 | 1.64980500 |
| H | 4.84612600 | 3.37904000 | 0.21291700 |
| H | 5.75772700 | 2.14248000 | -1.75033200 |
| I | 1.56865300 | -0.31282600 | 1.76566600 |
| C | 3.28329000 | -1.69602800 | -0.88776700 |
| O | 2.82666100 | -1.88515800 | -2.05152000 |
| O | 3.46260800 | -2.56018000 | -0.00557700 |
| O | 0.30354700 | 2.30111600 | 0.11686600 |
| C | 0.79729400 | 2.58491900 | -1.15555200 |
| H | 1.58253900 | 1.87643600 | -1.48823400 |
| H | 1.26101800 | 3.58752100 | -1.14459600 |
| H | 0.00955700 | 2.60479000 | -1.93614000 |
| C | -0.20451900 | -0.86803500 | -2.97131500 |
| H | -0.36586900 | 0.20117400 | -3.15142700 |
| H | -1.11902000 | -1.45340700 | -3.12596100 |
| H | 0.52968800 | -1.20012500 | -3.73137900 |

**21**

E (M06-SMD/BS1) = -1476.223768 au
H (M06-SMD/BS1) = -1475.777946 au
G (M06-SMD/BS1) = -1475.877997 au
E (M06-SMD/BS2//M06-SMD/BS1) = -1763.157064 au

| | | | |
|---|---|---|---|
| C | 4.27737900 | 0.27197100 | 1.79321500 |
| C | 3.20748400 | 0.10913300 | 0.93626400 |
| C | 3.43098800 | -0.52261600 | -0.31642600 |
| C | 4.72482400 | -0.93758600 | -0.70973900 |
| C | 5.79845800 | -0.74822200 | 0.19188900 |
| C | 5.56629700 | -0.16334800 | 1.41268900 |
| H | 4.12661800 | 0.73841100 | 2.76215200 |
| C | 4.86046300 | -1.51719000 | -1.99128300 |
| H | 6.79490400 | -1.07746900 | -0.09866100 |
| H | 6.38896100 | -0.02408500 | 2.11177000 |
| C | 3.76185600 | -1.66454500 | -2.80322700 |
| C | 2.50266300 | -1.25135200 | -2.33643900 |
| H | 5.84628000 | -1.84246500 | -2.32203300 |
| H | 3.83968100 | -2.10506500 | -3.79339200 |
| H | 1.60288400 | -1.38309000 | -2.93546600 |
| N | 2.35455200 | -0.70282100 | -1.14449300 |
| N | 1.89149600 | 0.56607700 | 1.12728200 |
| C | 1.67645500 | 1.86394500 | 1.49341500 |
| C | 0.36781200 | 2.40175700 | 0.93575400 |
| H | 0.46498900 | 3.49487700 | 0.84788800 |
| H | -0.47288800 | 2.19269200 | 1.60896300 |
| C | 0.13003200 | 1.85100700 | -0.42981600 |
| O | 2.45988700 | 2.56001100 | 2.13250400 |
| Pd | 0.57612700 | -0.40625500 | -0.11538900 |
| C | -4.75422600 | -0.70240900 | -0.98987500 |
| C | -3.74419200 | -0.27332200 | -0.12145300 |
| C | -3.02485900 | -1.26041200 | 0.54815400 |
| C | -3.27565200 | -2.61850900 | 0.37768100 |
| C | -4.30840800 | -3.01183900 | -0.46775300 |
| C | -5.04822900 | -2.05009000 | -1.15046900 |
| H | -5.31083900 | 0.05220600 | -1.54418500 |
| H | -2.66741900 | -3.36233000 | 0.88919000 |
| H | -4.52085700 | -4.07129200 | -0.59942500 |
| H | -5.84957200 | -2.35274600 | -1.82225500 |
| I | -1.35078700 | -0.76465900 | 1.78853300 |
| C | -3.46221100 | 1.21171000 | -0.01630000 |
| O | -3.40694300 | 1.82502200 | -1.12005000 |

| | | | |
|---|---|---|---|
| O | -3.30214500 | 1.71167600 | 1.11639300 |
| O | -0.49740000 | -1.52506500 | -1.38057600 |
| C | -1.07782200 | -0.82147100 | -2.43527800 |
| H | -1.80358600 | -0.05352200 | -2.10398100 |
| H | -1.63506200 | -1.53491500 | -3.06858900 |
| H | -0.33453500 | -0.32309100 | -3.09057600 |
| H | -0.89483200 | 1.58111400 | -0.70423000 |
| C | -1.73581700 | 4.11798100 | -2.33317100 |
| H | -1.48634500 | 3.18738000 | -2.87435900 |
| H | -0.98770900 | 4.87705000 | -2.59358100 |
| H | -2.71526300 | 4.46655400 | -2.69952500 |
| O | -1.72740900 | 3.94314500 | -0.93521500 |
| H | -2.42453900 | 3.26509900 | -0.76552600 |
| C | 1.03571700 | 2.24035400 | -1.52625600 |
| H | 0.74537600 | 3.27418400 | -1.79071800 |
| H | 0.90924500 | 1.62716200 | -2.42519700 |
| H | 2.09006100 | 2.27720800 | -1.22604600 |

**$TS_{21\text{-}22}$**

E (M06-SMD/BS1) = -1476.224708 au

H (M06-SMD/BS1) = -1475.779829 au

G (M06-SMD/BS1) = -1475.875779 au

E (M06-SMD/BS2//M06-SMD/BS1) = -1763.157196 au

| | | | |
|---|---|---|---|
| C | 4.30457100 | 0.95283300 | 1.51775800 |
| C | 3.24015000 | 0.46990000 | 0.78224500 |
| C | 3.48499900 | -0.55766600 | -0.16768100 |
| C | 4.79104800 | -1.04966300 | -0.39993900 |
| C | 5.85746900 | -0.52549700 | 0.36764700 |
| C | 5.60571800 | 0.44581600 | 1.30534100 |
| H | 4.14018500 | 1.72729700 | 2.26044900 |
| C | 4.94513200 | -2.04194500 | -1.39416900 |
| H | 6.86351300 | -0.90874000 | 0.20417500 |
| H | 6.42266500 | 0.84513100 | 1.90380500 |
| C | 3.85185900 | -2.49717900 | -2.09057800 |
| C | 2.58101600 | -1.98090500 | -1.78681400 |
| H | 5.94023700 | -2.43649400 | -1.59714500 |
| H | 3.94286100 | -3.25788500 | -2.86123100 |
| H | 1.68606800 | -2.34375300 | -2.28960700 |
| N | 2.41440800 | -1.04931500 | -0.86564000 |
| N | 1.91108800 | 0.92665900 | 0.81030700 |
| C | 1.65942300 | 2.26445500 | 0.76036800 |

| | | | |
|---|---|---|---|
| C | 0.31876400 | 2.57578200 | 0.11049200 |
| H | 0.39472500 | 3.58547100 | -0.32048200 |
| C | 0.04247800 | 1.62486000 | -0.99903700 |
| O | 2.42895600 | 3.14982600 | 1.12466400 |
| Pd | 0.62146100 | -0.46515700 | 0.00489900 |
| C | -4.69305300 | -1.15247000 | -0.94465700 |
| C | -3.71749500 | -0.44336100 | -0.23510800 |
| C | -3.01372700 | -1.14062800 | 0.74427700 |
| C | -3.24978100 | -2.48335200 | 1.02407200 |
| C | -4.24887200 | -3.15229000 | 0.32398600 |
| C | -4.97103400 | -2.48307600 | -0.66053500 |
| H | -5.23603400 | -0.63299900 | -1.73350400 |
| H | -2.65562200 | -3.00493500 | 1.77236300 |
| H | -4.44938900 | -4.19943800 | 0.54326800 |
| H | -5.74655600 | -3.00466800 | -1.21874600 |
| I | -1.39954700 | -0.22760300 | 1.81875000 |
| C | -3.45678600 | 1.00027200 | -0.61195900 |
| O | -3.27908700 | 1.20601400 | -1.84718200 |
| O | -3.43495000 | 1.85568200 | 0.29615600 |
| O | -0.37874100 | -2.00517100 | -0.78422400 |
| C | -0.98689800 | -1.72270300 | -2.00694100 |
| H | -1.67626200 | -0.85827200 | -1.96339900 |
| H | -1.59323300 | -2.59647800 | -2.30769000 |
| H | -0.26061000 | -1.53612100 | -2.82421000 |
| H | -0.96163200 | 1.20580200 | -1.09802000 |
| C | -2.19252800 | 4.53428700 | -1.26373900 |
| H | -3.14063700 | 4.93153900 | -1.66168100 |
| H | -1.44314000 | 5.33365400 | -1.31957600 |
| H | -2.35091000 | 4.28262400 | -0.20295200 |
| O | -1.72404300 | 3.43894500 | -2.01509900 |
| H | -2.40400100 | 2.72091000 | -1.97735100 |
| H | -0.49022600 | 2.58159800 | 0.85193000 |
| C | 0.88482800 | 1.63962000 | -2.20357800 |
| H | 0.48101400 | 2.46705200 | -2.81641100 |
| H | 0.78851500 | 0.72711000 | -2.80241200 |
| H | 1.93803700 | 1.86543700 | -1.99654400 |

**22**

E (M06-SMD/BS1) = -1476.287567 au

H (M06-SMD/BS1) = -1475.837983 au

G (M06-SMD/BS1) = -1475.933348 au

E (M06-SMD/BS2//M06-SMD/BS1) = -1763.214848 au

| | | | |
|---|---|---|---|
| C | 4.17404900 | 0.74701800 | 1.46234000 |
| C | 3.10400200 | 0.28695500 | 0.70997400 |
| C | 3.35485100 | -0.78261200 | -0.19740300 |
| C | 4.65776500 | -1.30212900 | -0.39531300 |
| C | 5.72367300 | -0.78027800 | 0.37261200 |
| C | 5.46726100 | 0.20913600 | 1.28896000 |
| H | 4.02155500 | 1.53624500 | 2.18851000 |
| C | 4.81363000 | -2.33588800 | -1.34599900 |
| H | 6.72518800 | -1.18487800 | 0.23379100 |
| H | 6.27675500 | 0.60300000 | 1.90179200 |
| C | 3.72335000 | -2.81538500 | -2.02887900 |
| C | 2.45475300 | -2.28122100 | -1.74922400 |
| H | 5.80805600 | -2.74628000 | -1.51972400 |
| H | 3.81422600 | -3.61113800 | -2.76350300 |
| H | 1.55739800 | -2.67108000 | -2.22634100 |
| N | 2.28625800 | -1.30406700 | -0.87643700 |
| N | 1.78329500 | 0.77152600 | 0.70506300 |
| C | 1.55617700 | 2.10063200 | 0.88075800 |
| C | 0.23568300 | 2.64382300 | 0.38239800 |
| H | 0.08384700 | 3.60626000 | 0.88893400 |
| C | 0.28068100 | 2.86320200 | -1.13369300 |
| O | 2.39158800 | 2.90261300 | 1.32697000 |
| Pd | 0.50802400 | -0.69881600 | -0.02448000 |
| C | -4.67683000 | -0.57976400 | -1.24277400 |
| C | -3.67175200 | -0.13321100 | -0.38118200 |
| C | -3.18227600 | -1.01059200 | 0.58625200 |
| C | -3.67710200 | -2.30322600 | 0.70770300 |
| C | -4.69971400 | -2.72082200 | -0.14079900 |
| C | -5.19774200 | -1.86108600 | -1.11476900 |
| H | -5.04800500 | 0.09454500 | -2.01341700 |
| H | -3.26111200 | -2.98468000 | 1.44722000 |
| H | -5.09507900 | -3.73020500 | -0.04362800 |
| H | -5.98790500 | -2.19255700 | -1.78534600 |
| I | -1.51288500 | -0.47666500 | 1.81384900 |
| C | -3.12644100 | 1.24290200 | -0.55134100 |
| O | -2.63800100 | 1.43764000 | -1.76545000 |
| O | -3.12251800 | 2.08682100 | 0.32695300 |
| O | -0.47885200 | -2.25218000 | -0.83005400 |
| C | -1.02927600 | -1.88565900 | -2.05594700 |
| H | -1.74498600 | -1.04422300 | -1.98080100 |

| | | | |
|---|---|---|---|
| H | -1.58842900 | -2.74181600 | -2.47638400 |
| H | -0.26569900 | -1.59998100 | -2.81007400 |
| H | 0.29039600 | 1.87689900 | -1.62781400 |
| C | -1.32234100 | 4.69876700 | -1.05933500 |
| H | -2.13253800 | 5.08189200 | -1.68841300 |
| H | -0.49589200 | 5.42229100 | -1.07306500 |
| H | -1.69671000 | 4.59370900 | -0.03146600 |
| O | -0.93613600 | 3.45491600 | -1.61732000 |
| H | -2.14618000 | 2.31484400 | -1.77229400 |
| H | -0.60638600 | 1.98522600 | 0.61989200 |
| C | 1.48581900 | 3.65594900 | -1.59953600 |
| H | 1.37868100 | 3.90714600 | -2.66229300 |
| H | 2.40087200 | 3.06018000 | -1.48395300 |
| H | 1.62089800 | 4.58361500 | -1.02750200 |

**$TS_{22-23}$**
E (M06-SMD/BS1) = -1476.278564 au
H (M06-SMD/BS1) = -1475.829362 au
G (M06-SMD/BS1) = -1475.921847 au
E (M06-SMD/BS2//M06-SMD/BS1) = -1763.203942 au

| | | | |
|---|---|---|---|
| C | 4.39793300 | 0.89066200 | 1.32871200 |
| C | 3.30976300 | 0.42087300 | 0.60258900 |
| C | 3.45679600 | -0.84541000 | -0.03108900 |
| C | 4.67362800 | -1.57234500 | 0.01618600 |
| C | 5.75899600 | -1.04033300 | 0.74624800 |
| C | 5.60143100 | 0.16035500 | 1.39361600 |
| H | 4.32571100 | 1.83548100 | 1.85270500 |
| C | 4.72747900 | -2.81127800 | -0.66201700 |
| H | 6.69349100 | -1.59765600 | 0.79106700 |
| H | 6.42375900 | 0.57432800 | 1.97548700 |
| C | 3.62380600 | -3.28222800 | -1.32766800 |
| C | 2.44314400 | -2.52131100 | -1.31212800 |
| H | 5.65533700 | -3.38224000 | -0.64096800 |
| H | 3.63698400 | -4.23124600 | -1.85707600 |
| H | 1.52821600 | -2.85769900 | -1.79744100 |
| N | 2.37314000 | -1.35880000 | -0.69019500 |
| N | 2.05584100 | 1.03495200 | 0.45206900 |
| C | 1.90192100 | 2.37005800 | 0.64066900 |
| C | 0.51085700 | 2.93682600 | 0.45496600 |
| H | 0.51511400 | 3.91425600 | 0.95393300 |
| C | 0.10109300 | 3.16570200 | -0.99344700 |

| | | | |
|---|---|---|---|
| O | 2.82060700 | 3.15558200 | 0.92643600 |
| Pd | 0.67300700 | -0.23257300 | -0.43413600 |
| C | -5.15464600 | -0.99411600 | -0.52207600 |
| C | -4.11155200 | -0.30105300 | 0.10628900 |
| C | -3.07789400 | -1.04615700 | 0.69026500 |
| C | -3.08953100 | -2.43735800 | 0.63594200 |
| C | -4.12072100 | -3.10114200 | -0.02198000 |
| C | -5.15626200 | -2.37839800 | -0.60381000 |
| H | -5.96224900 | -0.41539400 | -0.96666200 |
| H | -2.28875400 | -3.00688600 | 1.10340900 |
| H | -4.11098900 | -4.18859700 | -0.06971500 |
| H | -5.96876700 | -2.89082100 | -1.11454400 |
| I | -1.43982800 | -0.19220700 | 1.76186400 |
| C | -4.23491500 | 1.18087500 | 0.06149900 |
| O | -3.08178900 | 1.82292300 | -0.12034100 |
| O | -5.30458300 | 1.75561400 | 0.12564800 |
| O | -0.39265500 | -1.70374500 | -1.28290900 |
| C | -1.33678800 | -1.27013500 | -2.20161900 |
| H | -2.04735300 | -0.52979700 | -1.78025300 |
| H | -1.94799900 | -2.13187900 | -2.53003100 |
| H | -0.88353200 | -0.83151500 | -3.11276600 |
| O | -0.36809100 | 1.92594100 | -1.53006100 |
| H | -0.25097400 | 2.31484400 | 0.94053500 |
| C | -0.98723200 | 2.07719500 | -2.79319600 |
| H | -1.64943500 | 1.22181400 | -2.95292600 |
| H | -0.25128000 | 2.11629500 | -3.61014300 |
| H | -1.59648000 | 2.99509900 | -2.81832100 |
| H | -0.76203800 | 3.85760100 | -0.98266800 |
| C | 1.20934700 | 3.76425300 | -1.83730600 |
| H | 1.62505700 | 4.65153700 | -1.34256900 |
| H | 0.84712300 | 4.07105100 | -2.82587700 |
| H | 2.02295700 | 3.03748000 | -1.97995200 |
| H | -3.27204900 | 2.78247700 | -0.15092300 |

**23**

E (M06-SMD/BS1) = -1044.945254 au

H (M06-SMD/BS1) = -1044.61157 au

G (M06-SMD/BS1) = -1044.680585 au

E (M06-SMD/BS2//M06-SMD/BS1) = -1045.324937 au

| | | | |
|---|---|---|---|
| C | 2.02621900 | 2.54274100 | -0.52564600 |
| C | 1.32436500 | 1.39558000 | -0.18361600 |

| | | | |
|---|---|---|---|
| C | 2.07268300 | 0.19755600 | -0.01581300 |
| C | 3.48405400 | 0.17437400 | -0.12765700 |
| C | 4.15832400 | 1.36881400 | -0.46756500 |
| C | 3.42886300 | 2.51488500 | -0.66995800 |
| H | 1.49311800 | 3.47368900 | -0.67686100 |
| C | 4.14075300 | -1.05325100 | 0.11506500 |
| H | 5.24290200 | 1.35699500 | -0.56420300 |
| H | 3.93748600 | 3.43875500 | -0.94153600 |
| C | 3.41535000 | -2.16998100 | 0.44944900 |
| C | 2.01566700 | -2.08191800 | 0.51458000 |
| H | 5.22688500 | -1.09424500 | 0.03832000 |
| H | 3.89631000 | -3.12278900 | 0.65373000 |
| H | 1.39365600 | -2.94084500 | 0.76046100 |
| N | 1.37817400 | -0.94749600 | 0.27812100 |
| N | -0.05575600 | 1.26032000 | 0.01720200 |
| C | -0.84036000 | 2.30773400 | 0.36224800 |
| C | -2.28041300 | 1.97640800 | 0.69584700 |
| H | -2.79903900 | 2.93867100 | 0.77714400 |
| C | -3.04392900 | 1.09354100 | -0.27626700 |
| O | -0.45947300 | 3.48325100 | 0.45802600 |
| Pd | -0.62188000 | -0.70073300 | 0.18515200 |
| O | -0.98331800 | -2.67400000 | 0.37487800 |
| C | -0.76667800 | -3.37314900 | -0.81110900 |
| H | -1.39624100 | -3.02027700 | -1.65256700 |
| H | -1.00873800 | -4.44033200 | -0.65921700 |
| H | 0.28539500 | -3.33317600 | -1.15918400 |
| H | -2.70422400 | 1.26231800 | -1.31343900 |
| O | -2.73511300 | -0.29160000 | 0.04635600 |
| H | -2.32384900 | 1.50987000 | 1.69357000 |
| C | -4.53236800 | 1.34956600 | -0.16329800 |
| H | -5.11804100 | 0.79743900 | -0.90560800 |
| H | -4.72307900 | 2.41652300 | -0.32804000 |
| H | -4.89102700 | 1.08646900 | 0.84201200 |
| C | -3.42795100 | -1.23812400 | -0.77542000 |
| H | -3.23201200 | -1.03593000 | -1.83900200 |
| H | -4.50343800 | -1.19684600 | -0.57488800 |
| H | -3.05072300 | -2.22220600 | -0.49408200 |

**24**

E (M06-SMD/BS1) = -1273.931733 au

H (M06-SMD/BS1) = -1273.529979 au

G (M06-SMD/BS1) = -1273.615439 au
E (M06-SMD/BS2//M06-SMD/BS1) = -1274.407126 au

| | | | |
|---|---|---|---|
| C | 2.38548300 | -2.81994000 | -0.35694400 |
| C | 1.56687100 | -1.71877400 | -0.16324600 |
| C | 2.18437800 | -0.48401500 | 0.17309700 |
| C | 3.59168200 | -0.35263700 | 0.24544300 |
| C | 4.39185100 | -1.49859000 | 0.03389400 |
| C | 3.78682800 | -2.69904100 | -0.24851600 |
| H | 1.94730100 | -3.78051700 | -0.60308200 |
| C | 4.11609400 | 0.93075900 | 0.51811000 |
| H | 5.47491500 | -1.40700600 | 0.09898500 |
| H | 4.39562900 | -3.58814600 | -0.40502800 |
| C | 3.26890400 | 1.99646000 | 0.69516400 |
| C | 1.88141300 | 1.78668500 | 0.63885200 |
| H | 5.19649200 | 1.05901000 | 0.57617700 |
| H | 3.64346500 | 2.99770000 | 0.88999400 |
| H | 1.18222300 | 2.60660000 | 0.78784700 |
| N | 1.36355800 | 0.59208400 | 0.40440500 |
| N | 0.17174400 | -1.65851300 | -0.27109400 |
| C | -0.53466200 | -2.46756300 | -1.09655100 |
| C | -1.98735600 | -2.08370200 | -1.29232300 |
| H | -2.42392400 | -2.84342900 | -1.95192400 |
| C | -2.84452200 | -1.99468700 | -0.04358200 |
| O | -0.06897200 | -3.44010000 | -1.70488000 |
| Pd | -0.59086200 | 0.06110100 | 0.47823500 |
| O | -1.31224100 | 1.81975600 | 1.20759600 |
| C | -0.93718700 | 2.13494700 | 2.51784600 |
| H | -1.30564300 | 1.39207500 | 3.24838900 |
| H | -1.36504100 | 3.11180200 | 2.79996100 |
| H | 0.15837900 | 2.20734600 | 2.65315100 |
| H | -2.57263000 | -2.79347200 | 0.66777100 |
| O | -2.58196200 | -0.72637500 | 0.62286000 |
| H | -2.04791800 | -1.11945200 | -1.82457000 |
| C | -4.31230300 | -2.06340200 | -0.40648300 |
| H | -4.96855900 | -1.93436500 | 0.46149100 |
| H | -4.53659200 | -3.04204800 | -0.84793300 |
| H | -4.55278100 | -1.28652300 | -1.14523100 |
| C | -2.98721800 | -0.73596900 | 1.99773300 |
| H | -2.30467200 | -1.36370400 | 2.58855300 |
| H | -4.01092300 | -1.11560600 | 2.08778400 |
| H | -2.95864000 | 0.29785200 | 2.34771600 |

| | | | |
|---|---|---|---|
| O | -0.75107800 | 3.66320700 | -0.49518400 |
| C | -0.73358200 | 3.19262400 | -1.72867300 |
| O | -1.00614200 | 2.03169800 | -2.01341800 |
| C | -0.34358400 | 4.22348700 | -2.73874200 |
| H | -1.00335900 | 5.09571500 | -2.65782600 |
| H | -0.39506200 | 3.81073800 | -3.74956000 |
| H | 0.67766000 | 4.56914400 | -2.53426400 |
| H | -0.99317500 | 2.91533200 | 0.17336700 |

**$TS_{24-25}$**

E (M06-SMD/BS1) = -1273.931062 au

H (M06-SMD/BS1) = -1273.532867 au

G (M06-SMD/BS1) = -1273.616896 au

E (M06-SMD/BS2//M06-SMD/BS1) = -1274.407334 au

| | | | |
|---|---|---|---|
| C | -2.24621400 | 2.94780000 | -0.25020300 |
| C | -1.47918700 | 1.80279000 | -0.11060700 |
| C | -2.14829300 | 0.58558400 | 0.18304200 |
| C | -3.55887500 | 0.51518600 | 0.26776200 |
| C | -4.30710300 | 1.70475800 | 0.11405500 |
| C | -3.65056000 | 2.88664400 | -0.12817200 |
| H | -1.76675500 | 3.89628100 | -0.46413300 |
| C | -4.13856800 | -0.75401800 | 0.48880400 |
| H | -5.39247800 | 1.66038000 | 0.18889000 |
| H | -4.21936000 | 3.80811100 | -0.24163400 |
| C | -3.33927700 | -1.86455200 | 0.60044300 |
| C | -1.94428100 | -1.71661400 | 0.53392400 |
| H | -5.22290400 | -0.83547900 | 0.55670300 |
| H | -3.75704100 | -2.85637200 | 0.75027000 |
| H | -1.28321400 | -2.57685100 | 0.62106500 |
| N | -1.37425300 | -0.53563400 | 0.35363000 |
| N | -0.08917600 | 1.67999000 | -0.24320500 |
| C | 0.64306100 | 2.47558900 | -1.06135800 |
| C | 2.06801500 | 2.02157200 | -1.30068500 |
| H | 2.52793600 | 2.76873300 | -1.95862900 |
| C | 2.94341300 | 1.87210200 | -0.07131800 |
| O | 0.21469200 | 3.49034900 | -1.62453000 |
| Pd | 0.60206300 | -0.08732800 | 0.43554100 |
| O | 1.29177100 | -1.89418900 | 1.13140100 |
| C | 0.88297900 | -2.26438700 | 2.42591800 |
| H | 1.24689200 | -1.54616200 | 3.17764000 |
| H | 1.30014800 | -3.25353100 | 2.67309800 |

| | | | |
|---|---|---|---|
| H | -0.21458100 | -2.32765600 | 2.52743700 |
| H | 2.73239600 | 2.67845100 | 0.65167100 |
| O | 2.61874100 | 0.61485700 | 0.58956700 |
| H | 2.06783500 | 1.06322900 | -1.84687100 |
| C | 4.40566500 | 1.85647100 | -0.45990600 |
| H | 5.06571500 | 1.67394100 | 0.39566200 |
| H | 4.68228200 | 2.82610800 | -0.89136400 |
| H | 4.58578600 | 1.07708400 | -1.21295400 |
| C | 3.02932300 | 0.59711300 | 1.96347300 |
| H | 2.37354100 | 1.24572800 | 2.56168900 |
| H | 4.06697900 | 0.93774400 | 2.05038700 |
| H | 2.96450800 | -0.43764800 | 2.30617300 |
| O | 0.59361400 | -3.64365500 | -0.39413800 |
| C | 0.52494900 | -3.24161500 | -1.63434100 |
| O | 0.89724100 | -2.13013300 | -2.01944800 |
| C | -0.07743800 | -4.25019700 | -2.56678100 |
| H | 0.37131600 | -5.23757400 | -2.40796800 |
| H | 0.04854700 | -3.94359000 | -3.60904900 |
| H | -1.14997600 | -4.34129200 | -2.34756100 |
| H | 0.94743300 | -2.81624300 | 0.28451200 |

**25**

E (M06-SMD/BS1) = -1273.932506 au

H (M06-SMD/BS1) = -1273.530241 au

G (M06-SMD/BS1) = -1273.616406 au

E (M06-SMD/BS2//M06-SMD/BS1) = -1274.409261 au

| | | | |
|---|---|---|---|
| C | 1.86874400 | -3.17606000 | -0.14311200 |
| C | 1.23980800 | -1.94695000 | -0.04580400 |
| C | 2.03878900 | -0.80746200 | 0.22287500 |
| C | 3.44650700 | -0.89229200 | 0.32412100 |
| C | 4.05475900 | -2.16407800 | 0.21510600 |
| C | 3.27010400 | -3.27034300 | -0.00229600 |
| H | 1.28690100 | -4.06975100 | -0.33784200 |
| C | 4.16059000 | 0.31199600 | 0.51417600 |
| H | 5.13710400 | -2.24244600 | 0.30411100 |
| H | 3.73128300 | -4.25336900 | -0.08132000 |
| C | 3.48765900 | 1.50726100 | 0.58015300 |
| C | 2.08518000 | 1.51432400 | 0.49982100 |
| H | 5.24654500 | 0.27602700 | 0.59389900 |
| H | 4.01173000 | 2.45099900 | 0.70407500 |
| H | 1.51870400 | 2.44449000 | 0.54060700 |

| | | | |
|---|---|---|---|
| N | 1.39215000 | 0.39626600 | 0.35034600 |
| N | -0.12626200 | -1.66575400 | -0.20736700 |
| C | -0.92884100 | -2.37281200 | -1.04546200 |
| C | -2.27296800 | -1.74448800 | -1.34479800 |
| H | -2.79394800 | -2.42721300 | -2.02665900 |
| C | -3.16938400 | -1.49780000 | -0.14822500 |
| O | -0.60967400 | -3.44434900 | -1.57094000 |
| Pd | -0.61862900 | 0.16600900 | 0.42398600 |
| O | -1.15372400 | 2.07887900 | 1.13231600 |
| C | -0.70344000 | 2.45753800 | 2.42936500 |
| H | -1.08775900 | 1.73422600 | 3.15692800 |
| H | -1.09398700 | 3.45395400 | 2.67233800 |
| H | 0.39422300 | 2.47563700 | 2.49270700 |
| H | -3.11116000 | -2.34644200 | 0.55400000 |
| O | -2.68988900 | -0.32244400 | 0.56857000 |
| H | -2.13082700 | -0.79129100 | -1.88087700 |
| C | -4.59508100 | -1.24720200 | -0.58659500 |
| H | -5.24764100 | -0.98515600 | 0.25423100 |
| H | -5.00211500 | -2.15112200 | -1.05578600 |
| H | -4.62455100 | -0.43009900 | -1.32024200 |
| C | -3.10713900 | -0.31409800 | 1.94156000 |
| H | -2.53197400 | -1.05465800 | 2.51484100 |
| H | -4.17715000 | -0.54104300 | 2.00825800 |
| H | -2.93423600 | 0.69274100 | 2.32856700 |
| O | -0.09276100 | 3.74124900 | -0.49078900 |
| C | 0.00809000 | 3.20131100 | -1.64344500 |
| O | -0.60484000 | 2.17217900 | -2.00734400 |
| C | 0.98898600 | 3.84782700 | -2.59507000 |
| H | 0.97836900 | 4.93924200 | -2.49529600 |
| H | 0.78976200 | 3.56416300 | -3.63381800 |
| H | 2.00117200 | 3.50491800 | -2.33393700 |
| H | -0.79176400 | 2.76206600 | 0.44829400 |

**$TS_{25\text{-}26}$**

E (M06-SMD/BS1) = -1273.92026 au

H (M06-SMD/BS1) = -1273.518798 au

G (M06-SMD/BS1) = -1273.602731 au

E (M06-SMD/BS2//M06-SMD/BS1) = -1274.394729 au

| | | | |
|---|---|---|---|
| C | -3.08357300 | -2.13383700 | -0.03778200 |
| C | -2.00623400 | -1.26847900 | 0.03929400 |
| C | -2.26810200 | 0.12260000 | 0.07319300 |

| | | | |
|---|---|---|---|
| C | -3.58509100 | 0.63616400 | 0.07691700 |
| C | -4.66146900 | -0.27788800 | 0.00454300 |
| C | -4.40017100 | -1.62568300 | -0.05831300 |
| H | -2.91803300 | -3.20468200 | -0.07762400 |
| C | -3.73581700 | 2.03936700 | 0.15748800 |
| H | -5.68101000 | 0.10421400 | -0.00211200 |
| H | -5.22529200 | -2.33315700 | -0.12204400 |
| C | -2.62864900 | 2.84840400 | 0.23324400 |
| C | -1.34678900 | 2.27227900 | 0.20726100 |
| H | -4.73916000 | 2.46388600 | 0.16231200 |
| H | -2.71729300 | 3.92906600 | 0.30361000 |
| H | -0.44002800 | 2.87882000 | 0.24450900 |
| N | -1.18315900 | 0.96187700 | 0.12389800 |
| N | -0.64294900 | -1.59977600 | 0.10637200 |
| C | -0.19432000 | -2.70019600 | 0.76749700 |
| C | 1.28873200 | -2.72355600 | 1.07084600 |
| H | 1.49295300 | -3.67784800 | 1.57144300 |
| C | 2.21400400 | -2.61114800 | -0.12325500 |
| O | -0.92247600 | -3.62715400 | 1.13791900 |
| Pd | 0.55348500 | -0.00077500 | -0.14769500 |
| O | 1.38570300 | 1.71266600 | -1.71781900 |
| C | 0.51294400 | 1.99714200 | -2.79146600 |
| H | 0.40864900 | 1.08236300 | -3.38966600 |
| H | 0.91589100 | 2.79015100 | -3.43855400 |
| H | -0.48860400 | 2.30243700 | -2.44850500 |
| H | 1.83246400 | -3.22773700 | -0.95402800 |
| O | 2.22899300 | -1.23128100 | -0.59606900 |
| H | 1.53568300 | -1.92000400 | 1.78458900 |
| C | 3.62735700 | -3.00427400 | 0.24261100 |
| H | 4.32387200 | -2.85463900 | -0.59069900 |
| H | 3.65919000 | -4.06513200 | 0.51929600 |
| H | 3.97341700 | -2.40986000 | 1.09922500 |
| C | 2.54377400 | -1.15421100 | -1.99496200 |
| H | 1.70910700 | -1.55598400 | -2.58781300 |
| H | 3.45580700 | -1.72710100 | -2.19922700 |
| H | 2.70820400 | -0.10148000 | -2.23212800 |
| O | 1.69037000 | 3.46111300 | 0.28166600 |
| C | 2.09729500 | 2.65871700 | 1.16438400 |
| O | 2.04968000 | 1.39396500 | 1.07344900 |
| C | 2.66534300 | 3.22520700 | 2.44455500 |
| H | 2.90979300 | 4.28731900 | 2.33997300 |

| | | | |
|---|---|---|---|
| H | 3.55663000 | 2.66626600 | 2.75242300 |
| H | 1.91980200 | 3.11555900 | 3.24399600 |
| H | 1.43683600 | 2.48035400 | -1.08640200 |

**26**

E (M06-SMD/BS1) = -1158.278747 au
H (M06-SMD/BS1) = -1157.933293 au
G (M06-SMD/BS1) = -1158.008275 au
E (M06-SMD/BS2//M06-SMD/BS1) = -1158.702582 au

| | | | |
|---|---|---|---|
| C | -2.50051800 | -2.38732600 | -0.66932400 |
| C | -1.62984800 | -1.39871000 | -0.24124100 |
| C | -2.16791500 | -0.11220700 | 0.02599200 |
| C | -3.55221400 | 0.15592300 | -0.08565200 |
| C | -4.40883700 | -0.88174000 | -0.51922500 |
| C | -3.87825500 | -2.11526800 | -0.80834100 |
| H | -2.12453800 | -3.37851900 | -0.89501500 |
| C | -3.99557600 | 1.45447200 | 0.25204200 |
| H | -5.47471700 | -0.68214600 | -0.61753200 |
| H | -4.53000600 | -2.91735900 | -1.15093800 |
| C | -3.09632600 | 2.40190900 | 0.67604000 |
| C | -1.73302200 | 2.07320000 | 0.74298300 |
| H | -5.05736900 | 1.68649400 | 0.17572900 |
| H | -3.41117100 | 3.40448600 | 0.95219100 |
| H | -0.98843300 | 2.79835100 | 1.06537000 |
| N | -1.29263000 | 0.86812400 | 0.42064100 |
| N | -0.24883800 | -1.50570700 | -0.01931000 |
| C | 0.35249400 | -2.66586900 | 0.34325100 |
| C | 1.78318000 | -2.53358700 | 0.82427500 |
| H | 2.12770900 | -3.54796700 | 1.05900900 |
| C | 2.77028000 | -1.91659200 | -0.14662300 |
| O | -0.19614800 | -3.77448000 | 0.33632300 |
| Pd | 0.62849800 | 0.28690100 | 0.24199200 |
| H | 2.57299200 | -2.27513400 | -1.17099800 |
| O | 2.58705400 | -0.46965500 | -0.16397400 |
| H | 1.80887000 | -1.95649500 | 1.76292600 |
| C | 4.19166700 | -2.22049500 | 0.27342300 |
| H | 4.93152300 | -1.72441800 | -0.36508400 |
| H | 4.37156200 | -3.30069000 | 0.21249100 |
| H | 4.35425900 | -1.89942400 | 1.31144600 |
| C | 3.08323800 | 0.11903500 | -1.37582200 |
| H | 2.41943400 | -0.13119900 | -2.21508100 |

| | | | |
|---|---|---|---|
| H | 4.09675000 | -0.24349600 | -1.58064800 |
| H | 3.11547700 | 1.20002800 | -1.22581000 |
| O | 0.84301100 | 2.79899800 | -1.45936700 |
| C | 1.33195400 | 3.02739800 | -0.34567900 |
| O | 1.41969000 | 2.15747200 | 0.61071600 |
| C | 1.86198200 | 4.38985200 | 0.01704700 |
| H | 1.90445800 | 5.03957500 | -0.86236500 |
| H | 2.85803700 | 4.30871300 | 0.46788800 |
| H | 1.20195300 | 4.84616000 | 0.76701300 |

**$TS_{26\text{-}27}$**

E (M06-SMD/BS1) = -1387.247459 au

H (M06-SMD/BS1) = -1386.834303 au

G (M06-SMD/BS1) = -1386.921115 au

E (M06-SMD/BS2//M06-SMD/BS1) = -1387.765388 au

| | | | |
|---|---|---|---|
| C | -3.14947000 | -2.33195000 | 0.29395700 |
| C | -2.20659200 | -1.31880500 | 0.20041600 |
| C | -2.68106100 | 0.01415700 | 0.14797600 |
| C | -4.06476200 | 0.31770000 | 0.14821100 |
| C | -4.99175400 | -0.74516200 | 0.21884700 |
| C | -4.52695200 | -2.03570700 | 0.29903400 |
| H | -2.82865600 | -3.36492200 | 0.36409100 |
| C | -4.43744900 | 1.67937000 | 0.10246700 |
| H | -6.05704300 | -0.51993100 | 0.22294000 |
| H | -5.23220600 | -2.86214800 | 0.37122900 |
| C | -3.47224300 | 2.65444100 | 0.07394400 |
| C | -2.11818600 | 2.28230700 | 0.08684800 |
| H | -5.49585100 | 1.93763300 | 0.09925500 |
| H | -3.72645100 | 3.71052600 | 0.04661800 |
| H | -1.33223100 | 3.03168000 | 0.07755800 |
| N | -1.74405300 | 1.01359700 | 0.11518600 |
| N | -0.80837200 | -1.46241500 | 0.21513000 |
| C | -0.21625100 | -2.60357100 | -0.24333600 |
| C | 1.28224400 | -2.72764400 | -0.10240800 |
| H | 1.48909700 | -3.80464600 | -0.05574200 |
| C | 2.05939800 | -2.15415700 | -1.27798800 |
| O | -0.84081400 | -3.53735700 | -0.76434800 |
| Pd | 0.15490800 | 0.32291500 | 0.30328300 |
| H | 1.55822700 | -2.44137800 | -2.22107700 |
| O | 2.03815100 | -0.72531200 | -1.17415500 |
| H | 1.64993600 | -2.26759300 | 0.82243500 |

| | | | |
|---|---|---|---|
| C | 3.49198100 | -2.64684400 | -1.27054900 |
| H | 4.08602400 | -2.17796300 | -2.06508800 |
| H | 3.52908700 | -3.73343800 | -1.41946700 |
| H | 3.96291500 | -2.41394600 | -0.30375200 |
| C | 2.17241300 | -0.07542900 | -2.42362600 |
| H | 1.24020400 | -0.14689300 | -3.00717900 |
| H | 2.99698600 | -0.50212500 | -3.01360400 |
| H | 2.39793700 | 0.97880400 | -2.23052000 |
| O | 0.42951800 | 2.65873200 | -1.78199300 |
| C | 1.11755900 | 2.88207000 | -0.78724600 |
| O | 1.07550300 | 2.17467800 | 0.31292400 |
| C | 2.12342200 | 3.99865400 | -0.74112400 |
| H | 1.98966700 | 4.67772800 | -1.58835400 |
| H | 3.13264200 | 3.56449200 | -0.78973800 |
| H | 2.05187000 | 4.55357400 | 0.20129000 |
| O | 3.41078200 | 1.19112900 | 1.17904200 |
| C | 3.04365000 | 0.06584000 | 1.75459500 |
| O | 1.87448800 | -0.32480800 | 1.77792600 |
| C | 4.15897000 | -0.67846000 | 2.39857200 |
| H | 5.05443100 | -0.64767200 | 1.76821500 |
| H | 3.86425100 | -1.71196000 | 2.60013300 |
| H | 4.40858100 | -0.18777600 | 3.34889400 |
| H | 2.59124000 | 1.65820900 | 0.84191000 |

**27**

E (M06-SMD/BS1) = -1387.254178 au

H (M06-SMD/BS1) = -1386.839304 au

G (M06-SMD/BS1) = -1386.927731 au

E (M06-SMD/BS2//M06-SMD/BS1) = -1387.773875 au

| | | | |
|---|---|---|---|
| C | 2.74251100 | 2.58252800 | 0.80753300 |
| C | 1.97965000 | 1.47958500 | 0.45646900 |
| C | 2.67044600 | 0.32173400 | 0.02333700 |
| C | 4.07561600 | 0.30665300 | -0.15243500 |
| C | 4.81166700 | 1.46399200 | 0.18530200 |
| C | 4.14601000 | 2.56194000 | 0.67384200 |
| H | 2.25938900 | 3.47817800 | 1.18091700 |
| C | 4.66019800 | -0.88074800 | -0.64534400 |
| H | 5.89375400 | 1.45845900 | 0.06457900 |
| H | 4.70511000 | 3.45152600 | 0.95957400 |
| C | 3.87352400 | -1.97142800 | -0.92224200 |
| C | 2.49304300 | -1.90115900 | -0.67858100 |

| | | | |
|---|---|---|---|
| H | 5.73840500 | -0.91529000 | -0.79782800 |
| H | 4.29406800 | -2.89676100 | -1.30600500 |
| H | 1.85082700 | -2.76266200 | -0.84226000 |
| N | 1.92127900 | -0.79933400 | -0.22008300 |
| N | 0.57513600 | 1.37132300 | 0.49396900 |
| C | -0.17788900 | 2.47168800 | 0.19502700 |
| C | -1.67696700 | 2.31153600 | 0.15732900 |
| H | -2.08899500 | 3.32713400 | 0.22176200 |
| C | -2.15975700 | 1.64478700 | -1.13363300 |
| O | 0.30678600 | 3.58055600 | -0.07060900 |
| Pd | 0.03177100 | -0.62211100 | 0.46737200 |
| H | -1.66912800 | 0.66127400 | -1.22568000 |
| C | -4.43611000 | 2.39922900 | -0.94008500 |
| H | -5.44626700 | 1.97465400 | -0.93263500 |
| H | -4.36090000 | 3.09431000 | -1.79047600 |
| H | -4.29486700 | 2.96695200 | -0.00682900 |
| O | -3.54265400 | 1.31383900 | -1.05179500 |
| H | -2.03283900 | 1.73850400 | 1.02007000 |
| C | -1.82321500 | 2.46290500 | -2.36604300 |
| H | -2.29916000 | 2.02724800 | -3.25430500 |
| H | -0.73778200 | 2.46580200 | -2.53177400 |
| H | -2.14491600 | 3.50915300 | -2.27105200 |
| O | -0.17444200 | -2.68430700 | 0.50759200 |
| C | -0.74448000 | -3.23690100 | -0.50306000 |
| O | -1.38854900 | -2.60415000 | -1.36670100 |
| C | -0.56754500 | -4.72241200 | -0.61855600 |
| H | 0.32189400 | -4.91906600 | -1.23366100 |
| H | -1.42993400 | -5.17407600 | -1.11893500 |
| H | -0.40952300 | -5.18550800 | 0.36049700 |
| O | -3.23074600 | -1.44502100 | 0.12683100 |
| C | -2.93120600 | -0.76723300 | 1.19496800 |
| O | -1.78258400 | -0.41417800 | 1.51380200 |
| C | -4.09669600 | -0.38511100 | 2.03175500 |
| H | -4.60042400 | 0.45797600 | 1.53705400 |
| H | -3.77288000 | -0.07191900 | 3.02717500 |
| H | -4.81406800 | -1.20960200 | 2.09655300 |
| H | -2.43338900 | -1.68651900 | -0.43951700 |

**$TS_{27-28}$**

E (M06-SMD/BS1) = -1387.240301 au

H (M06-SMD/BS1) = -1386.831576 au

G (M06-SMD/BS1) = -1386.920376 au

E (M06-SMD/BS2//M06-SMD/BS1) = -1387.758245 au

| | | | |
|---|---|---|---|
| C | -2.67638400 | -2.42215800 | 0.78886900 |
| C | -1.91592200 | -1.33641200 | 0.42948800 |
| C | -2.55449700 | -0.17046500 | -0.04204000 |
| C | -3.95861800 | -0.12288200 | -0.19963000 |
| C | -4.71527300 | -1.25579800 | 0.18261800 |
| C | -4.08375100 | -2.37053400 | 0.67942400 |
| H | -2.18375200 | -3.32895800 | 1.13767800 |
| C | -4.51412900 | 1.06639500 | -0.72460600 |
| H | -5.79881700 | -1.22183800 | 0.07955400 |
| H | -4.66767200 | -3.23898800 | 0.97731100 |
| C | -3.69609400 | 2.12065000 | -1.05012500 |
| C | -2.31266600 | 2.01751400 | -0.82280200 |
| H | -5.59244200 | 1.13151200 | -0.86483900 |
| H | -4.09466600 | 3.04411400 | -1.46081900 |
| H | -1.63852000 | 2.84751700 | -1.02528600 |
| N | -1.76932800 | 0.91647600 | -0.33405400 |
| N | -0.48024800 | -1.34936100 | 0.41810800 |
| C | 0.05440000 | -1.99781900 | -0.71913700 |
| C | 1.54611300 | -2.17321200 | -0.75530900 |
| H | 1.73957900 | -3.18098500 | -1.14865000 |
| C | 2.20844700 | -1.10682900 | -1.65478600 |
| O | -0.65478400 | -2.38473400 | -1.63686000 |
| Pd | 0.09525800 | 0.67081400 | 0.41770400 |
| H | 1.65079400 | -0.16230300 | -1.54262500 |
| C | 4.46674200 | -1.80015300 | -1.26050000 |
| H | 5.35987300 | -1.43965600 | -0.73888800 |
| H | 4.74116300 | -2.02787300 | -2.30188100 |
| H | 4.13311900 | -2.73005400 | -0.77243600 |
| O | 3.50185300 | -0.77440000 | -1.17243900 |
| H | 1.97829500 | -2.11505600 | 0.24862700 |
| C | 2.19634100 | -1.50648200 | -3.11582600 |
| H | 2.69767800 | -0.74600800 | -3.72856500 |
| H | 1.16022400 | -1.60239900 | -3.46461000 |
| H | 2.68960000 | -2.47412100 | -3.28015300 |
| O | 0.43512400 | 2.68198400 | 0.51222400 |
| C | 1.39884400 | 3.11488600 | -0.24389700 |
| O | 2.07454300 | 2.40373300 | -0.99382800 |
| C | 1.62427000 | 4.59977600 | -0.13724900 |
| H | 0.72350500 | 5.13442800 | -0.46438500 |

| | | | |
|---|---|---|---|
| H | 2.47308400 | 4.90768300 | -0.75504800 |
| H | 1.80819900 | 4.88076000 | 0.90674500 |
| O | 1.00872100 | -1.52635900 | 2.41496400 |
| C | 1.88683700 | -0.59902600 | 2.33411400 |
| O | 1.82995200 | 0.36741600 | 1.52361200 |
| C | 3.08214200 | -0.70677300 | 3.21700800 |
| H | 3.49788500 | 0.28114100 | 3.43429900 |
| H | 3.84376100 | -1.28915300 | 2.67897700 |
| H | 2.83715200 | -1.23727600 | 4.14184500 |
| H | 0.22302800 | -1.50491400 | 1.52535500 |

**28**

E (M06-SMD/BS1) = -1387.254974 au

H (M06-SMD/BS1) = -1386.840332 au

G (M06-SMD/BS1) = -1386.930035 au

E (M06-SMD/BS2//M06-SMD/BS1) = -1387.771562 au

| | | | |
|---|---|---|---|
| C | 0.04651000 | 3.24063300 | 1.34467900 |
| C | -0.36841100 | 2.01278200 | 0.89123400 |
| C | -1.53779100 | 1.89578600 | 0.11632600 |
| C | -2.29050400 | 3.05169400 | -0.20131700 |
| C | -1.84071100 | 4.30607200 | 0.27142600 |
| C | -0.69697500 | 4.39625100 | 1.02728100 |
| H | 0.95013700 | 3.31437500 | 1.94937300 |
| C | -3.45954800 | 2.87907200 | -0.97691900 |
| H | -2.42037200 | 5.19448000 | 0.02516700 |
| H | -0.35514000 | 5.36200500 | 1.39197400 |
| C | -3.82364000 | 1.62299700 | -1.39340700 |
| C | -3.02419800 | 0.52271200 | -1.04032600 |
| H | -4.05756000 | 3.75220900 | -1.23443100 |
| H | -4.71533400 | 1.45631000 | -1.99108700 |
| H | -3.27505300 | -0.48746700 | -1.36045200 |
| N | -1.92740800 | 0.65495400 | -0.31398000 |
| N | 0.35079000 | 0.79061200 | 1.21526200 |
| H | 0.30461100 | 0.58276700 | 2.22096000 |
| C | 1.73489600 | 0.66390900 | 0.80481300 |
| C | 2.06699500 | 1.17939800 | -0.56196000 |
| H | 2.12466400 | 2.27934500 | -0.52465100 |
| H | 1.24270400 | 0.93675100 | -1.24933500 |
| C | 3.39088800 | 0.63586500 | -1.07130500 |
| H | 3.44393300 | -0.44649300 | -0.85460000 |
| C | 3.54037900 | 0.86151400 | -2.56099700 |

| | | | |
|---|---|---|---|
| H | 2.78446300 | 0.29011400 | -3.11676900 |
| H | 4.53036300 | 0.53726700 | -2.90685200 |
| H | 3.42115300 | 1.92688200 | -2.80374300 |
| O | 2.49988600 | 0.09873100 | 1.55182400 |
| Pd | -0.67048300 | -0.83185000 | 0.27325400 |
| O | -1.70664200 | -2.27602300 | -0.71551300 |
| C | -2.58684200 | -2.88967700 | 0.01685800 |
| O | -2.80227200 | -2.63929800 | 1.20712000 |
| O | 0.63456400 | -2.23765800 | 0.96506700 |
| C | 1.55057500 | -2.57687400 | 0.10967700 |
| O | 1.59380600 | -2.17958800 | -1.06119600 |
| C | -3.36625900 | -3.93684200 | -0.73208500 |
| H | -4.02869000 | -3.44386100 | -1.45599100 |
| H | -3.97156000 | -4.53443100 | -0.04409700 |
| H | -2.69190500 | -4.58910600 | -1.29870100 |
| C | 2.62655400 | -3.46195800 | 0.67442000 |
| H | 3.11031700 | -4.03691000 | -0.12154400 |
| H | 2.23808300 | -4.13497300 | 1.44585300 |
| H | 3.38385500 | -2.81603400 | 1.14257600 |
| O | 4.41646300 | 1.30667900 | -0.34993800 |
| C | 5.56118000 | 0.51268500 | -0.15258300 |
| H | 5.33224900 | -0.36943100 | 0.46990200 |
| H | 6.30403200 | 1.12837700 | 0.36682300 |
| H | 5.99854500 | 0.16507800 | -1.10285600 |

**HOAc**

E (M06-SMD/BS1) = -228.9646732 au
H (M06-SMD/BS1) = -228.897569 au
G (M06-SMD/BS1) = -228.930026 au
E (M06-SMD/BS2//M06-SMD/BS1) = -229.0665165 au

| | | | |
|---|---|---|---|
| O | 0.76847600 | -1.03775700 | 0.00002200 |
| C | 0.08985300 | 0.11776600 | 0.00001000 |
| O | 0.64331200 | 1.19948900 | 0.00000100 |
| C | -1.38381000 | -0.10686600 | -0.00001000 |
| H | -1.67121300 | -0.69052900 | 0.88301500 |
| H | -1.91115100 | 0.84997700 | 0.00013400 |
| H | -1.67107300 | -0.69032000 | -0.88322100 |
| H | 1.72287000 | -0.82837900 | -0.00010600 |

**MeOH**

E (M06-SMD/BS1) = -115.6494595 au

H (M06-SMD/BS1) = -115.593808 au
G (M06-SMD/BS1) = -115.620851 au
E (M06-SMD/BS2//M06-SMD/BS1) = -115.7071077 au

| | | | |
|---|---|---|---|
| O | -0.74575100 | 0.12375200 | -0.00005100 |
| H | -1.13116900 | -0.76443900 | -0.00017800 |
| C | 0.65877100 | -0.01928100 | 0.00000700 |
| H | 1.09045600 | 0.98821200 | 0.00093000 |
| H | 1.02639900 | -0.54973100 | 0.89237600 |
| H | 1.02769400 | -0.54837100 | -0.89275900 |

**PhICOOH**
E (M06-SMD/BS1) = -431.3364793 au
H (M06-SMD/BS1) = -431.221983 au
G (M06-SMD/BS1) = -431.267989 au
E (M06-SMD/BS2//M06-SMD/BS1) = -717.8839585 au

| | | | |
|---|---|---|---|
| C | 2.65757500 | -0.16515700 | 0.02901900 |
| C | 1.33759400 | 0.30898000 | 0.03787600 |
| C | 0.29526000 | -0.62847400 | 0.04153700 |
| C | 0.57234500 | -1.99196100 | 0.03685500 |
| C | 1.89024000 | -2.43807900 | 0.04540100 |
| C | 2.93684100 | -1.52317900 | 0.04142100 |
| H | 3.47243200 | 0.55518100 | 0.03060700 |
| H | -0.24280800 | -2.71236800 | 0.02392300 |
| H | 2.09171900 | -3.50789100 | 0.05000200 |
| H | 3.96978100 | -1.86464800 | 0.04567100 |
| I | -1.78409700 | -0.11719800 | -0.05123700 |
| C | 1.12456600 | 1.77759200 | 0.09092200 |
| O | 2.12811000 | 2.45261200 | -0.48150300 |
| O | 0.17563900 | 2.33444600 | 0.60538500 |
| H | 1.94949400 | 3.40641000 | -0.36389700 |

**13_H**
E (M06-SMD/BS1) = -1233.516136 au
H (M06-SMD/BS1) = -1233.166631 au
G (M06-SMD/BS1) = -1233.250029 au
E (M06-SMD/BS2//M06-SMD/BS1) = -1233.971357 au

| | | | |
|---|---|---|---|
| C | 2.80357300 | -1.50207900 | 1.30039200 |
| C | 1.79865700 | -0.78776500 | 0.69604200 |
| C | 2.02953000 | 0.52846700 | 0.25063700 |
| C | 3.30957900 | 1.11200200 | 0.40486000 |
| C | 4.32999500 | 0.35470900 | 1.02545500 |

| | | | |
|---|---|---|---|
| C | 4.07807500 | -0.92117200 | 1.46844200 |
| H | 2.61102200 | -2.51944700 | 1.63989200 |
| C | 3.48800600 | 2.43214600 | -0.06792600 |
| H | 5.31341800 | 0.80611200 | 1.14789400 |
| H | 4.86337400 | -1.49888900 | 1.95036300 |
| C | 2.43683400 | 3.09908300 | -0.64601100 |
| C | 1.18977700 | 2.45955100 | -0.75060400 |
| H | 4.46372000 | 2.90465600 | 0.03685100 |
| H | 2.54146500 | 4.11426300 | -1.01830600 |
| H | 0.32857000 | 2.96124400 | -1.18968500 |
| N | 0.99603900 | 1.22426800 | -0.32110800 |
| N | 0.47582600 | -1.34423200 | 0.48311200 |
| H | -0.02753100 | -1.57109900 | 1.35681200 |
| C | 0.32441600 | -2.44128000 | -0.44830300 |
| C | 1.26293200 | -2.45898000 | -1.61517600 |
| H | 2.25588000 | -2.75072500 | -1.23825300 |
| H | 1.38025900 | -1.43092100 | -1.98969800 |
| C | 0.80070200 | -3.40433600 | -2.70540000 |
| H | 0.72434600 | -4.43308100 | -2.33396900 |
| H | -0.18325400 | -3.11090100 | -3.09282900 |
| O | -0.55966300 | -3.24475600 | -0.25152500 |
| Pd | -0.76696300 | 0.21410500 | -0.25295200 |
| O | -1.84680100 | 1.78454700 | -0.96410100 |
| C | -2.33369800 | 2.53865000 | -0.02457800 |
| O | -2.17799100 | 2.33436900 | 1.18407200 |
| O | -2.50484500 | -0.84342800 | -0.11661200 |
| C | -2.72723900 | -1.40530100 | 1.03058600 |
| O | -1.98573400 | -1.30577200 | 2.01829600 |
| C | -3.09242300 | 3.73148600 | -0.54025000 |
| H | -2.38248100 | 4.44454600 | -0.98025200 |
| H | -3.63431700 | 4.22529100 | 0.27176800 |
| H | -3.79158200 | 3.43607600 | -1.33074800 |
| C | -3.96589000 | -2.25666700 | 1.06509000 |
| H | -4.29132900 | -2.42352200 | 2.09650500 |
| H | -3.72631200 | -3.22947900 | 0.61324000 |
| H | -4.77642800 | -1.80849400 | 0.48063100 |
| H | 1.51173400 | -3.39546000 | -3.53902000 |

**$TS_{13\text{-}14\_H}$**

E (M06-SMD/BS1) = -1233.503916 au

H (M06-SMD/BS1) = -1233.160993 au

G (M06-SMD/BS1) = -1233.239564 au
E (M06-SMD/BS2//M06-SMD/BS1) = -1233.959952 au

| | | | |
|---|---|---|---|
| C | 2.73440400 | -1.04181400 | 1.49887300 |
| C | 1.72043400 | -0.52790500 | 0.72520900 |
| C | 1.79416000 | 0.81452000 | 0.29191300 |
| C | 2.92102000 | 1.61331300 | 0.59488800 |
| C | 3.95369800 | 1.05049900 | 1.38120100 |
| C | 3.85078600 | -0.24388500 | 1.83069600 |
| H | 2.67633900 | -2.07554000 | 1.83824500 |
| C | 2.93370900 | 2.93787000 | 0.10065200 |
| H | 4.81849600 | 1.66451800 | 1.62810200 |
| H | 4.64044900 | -0.67184900 | 2.44466000 |
| C | 1.87345600 | 3.40538100 | -0.63618700 |
| C | 0.77174200 | 2.56436500 | -0.86988700 |
| H | 3.79040200 | 3.57524500 | 0.31616900 |
| H | 1.85584700 | 4.41803900 | -1.02924200 |
| H | -0.10215100 | 2.90984100 | -1.42017100 |
| N | 0.73744700 | 1.32078400 | -0.42318700 |
| N | 0.58422800 | -1.29422100 | 0.30365900 |
| H | -0.26048200 | -1.74277200 | 1.13513800 |
| C | 0.75954000 | -2.25219900 | -0.72912100 |
| C | 2.05130900 | -2.24271000 | -1.49971800 |
| H | 2.87234800 | -2.48104900 | -0.80749000 |
| H | 2.24163200 | -1.20848900 | -1.82939600 |
| C | 2.03862600 | -3.20248300 | -2.67143900 |
| H | 1.88243300 | -4.23564500 | -2.33818100 |
| H | 1.23995800 | -2.95482100 | -3.38184000 |
| O | -0.14653600 | -3.03410800 | -0.97030200 |
| Pd | -0.85677900 | 0.07436600 | -0.33616700 |
| O | -2.18720600 | 1.47394000 | -0.99902700 |
| C | -2.65822700 | 2.24740600 | -0.06921500 |
| O | -2.36450200 | 2.16038800 | 1.12848100 |
| O | -2.44733800 | -1.17561500 | 0.11792500 |
| C | -2.33284700 | -1.96868500 | 1.10043400 |
| O | -1.24767500 | -2.19050800 | 1.72868700 |
| C | -3.59423700 | 3.30934900 | -0.58107300 |
| H | -3.01990500 | 4.04692200 | -1.15744000 |
| H | -4.09465600 | 3.81758200 | 0.24857100 |
| H | -4.34011800 | 2.87606800 | -1.25727300 |
| C | -3.54689100 | -2.71380600 | 1.54637700 |
| H | -3.43319100 | -3.76897500 | 1.26603600 |

| | | | |
|---|---|---|---|
| H | -4.45305400 | -2.31327500 | 1.08479300 |
| H | -3.62675000 | -2.67081700 | 2.63832300 |
| H | 2.99451600 | -3.15982100 | -3.20635800 |

**15_H**

E (M06-SMD/BS1) = -1004.516779 au
H (M06-SMD/BS1) = -1004.23754 au
G (M06-SMD/BS1) = -1004.307616 au
E (M06-SMD/BS2//M06-SMD/BS1) = -1004.884014 au

| | | | |
|---|---|---|---|
| C | 3.26330000 | 0.85708800 | -0.25297600 |
| C | 1.92986100 | 0.52626600 | -0.06019300 |
| C | 1.60419700 | -0.85400100 | 0.03663900 |
| C | 2.58740100 | -1.86671500 | -0.06254500 |
| C | 3.93354100 | -1.48550700 | -0.26100900 |
| C | 4.24501900 | -0.15107000 | -0.35087200 |
| H | 3.55276600 | 1.89773900 | -0.33099000 |
| C | 2.15859100 | -3.20921000 | 0.03993600 |
| H | 4.69712600 | -2.25761100 | -0.34027500 |
| H | 5.27917100 | 0.15336300 | -0.50382600 |
| C | 0.83008900 | -3.49994300 | 0.23161700 |
| C | -0.09459700 | -2.44827300 | 0.32416000 |
| H | 2.89856500 | -4.00533400 | -0.03576600 |
| H | 0.47545600 | -4.52357800 | 0.31393100 |
| H | -1.15584700 | -2.63021000 | 0.48461300 |
| N | 0.28609000 | -1.18512600 | 0.22887400 |
| N | 0.82945500 | 1.38738000 | 0.04984900 |
| C | 0.88821500 | 2.74458800 | -0.04231500 |
| C | -0.42588300 | 3.48396300 | 0.09456300 |
| H | -0.40892100 | 4.29330800 | -0.64691800 |
| H | -0.40375900 | 3.97892100 | 1.07710300 |
| C | -1.70309300 | 2.68019500 | -0.05224700 |
| H | -1.79938100 | 2.19376000 | -1.03499300 |
| H | -1.89286400 | 1.99626200 | 0.83163400 |
| O | 1.93174500 | 3.38759600 | -0.20022600 |
| Pd | -0.90926000 | 0.42838800 | 0.30342300 |
| O | -2.62656400 | -0.65411800 | 0.63007400 |
| C | -3.27585200 | -0.96796000 | -0.44775700 |
| O | -2.94082500 | -0.62480400 | -1.58823700 |
| C | -4.47944400 | -1.83920300 | -0.20899400 |
| H | -4.14057700 | -2.86706500 | -0.02001100 |
| H | -5.13481300 | -1.84326600 | -1.08540600 |

| | | | |
|---|---|---|---|
| H | -5.03680300 | -1.50988600 | 0.67496700 |
| H | -2.59588000 | 3.31128300 | 0.04646800 |

**$TS_{15\text{-}16\_H}$**

E (M06-SMD/BS1) = -1004.490347 au

H (M06-SMD/BS1) = -1004.216366 au

G (M06-SMD/BS1) = -1004.282263 au

E (M06-SMD/BS2//M06-SMD/BS1) = -1004.858066 au

| | | | |
|---|---|---|---|
| C | -3.08878300 | 1.48021600 | -0.12175400 |
| C | -1.86950600 | 0.82413800 | -0.03575500 |
| C | -1.88242400 | -0.60064400 | -0.01054000 |
| C | -3.09293500 | -1.33164200 | -0.07556800 |
| C | -4.31356000 | -0.62428500 | -0.16448200 |
| C | -4.29291900 | 0.74896400 | -0.18577100 |
| H | -3.11288300 | 2.56360300 | -0.14206500 |
| C | -3.00719200 | -2.74232100 | -0.04451700 |
| H | -5.24755000 | -1.18208400 | -0.21241300 |
| H | -5.22812400 | 1.30290300 | -0.25246800 |
| C | -1.78355900 | -3.35826000 | 0.04528900 |
| C | -0.62410200 | -2.56699500 | 0.10420900 |
| H | -3.92547500 | -3.32697700 | -0.09230600 |
| H | -1.69025200 | -4.44059500 | 0.07130400 |
| H | 0.36522000 | -3.01627900 | 0.17408900 |
| N | -0.67538600 | -1.24660700 | 0.07756000 |
| N | -0.59581700 | 1.39471500 | 0.02784700 |
| C | -0.30558600 | 2.71790900 | -0.09739400 |
| C | 1.17617300 | 3.00366900 | -0.09795000 |
| C | 2.02791100 | 1.93067300 | 0.56315000 |
| H | 1.81611600 | 1.83428900 | 1.63864900 |
| O | -1.14111700 | 3.61866300 | -0.23624100 |
| Pd | 0.89751300 | 0.08358900 | 0.19763400 |
| O | 2.38550400 | -1.39129500 | 0.27709600 |
| C | 3.48090500 | -1.02031500 | -0.24328400 |
| O | 3.67820200 | 0.16849400 | -0.63950500 |
| C | 4.58749500 | -2.01169800 | -0.38760700 |
| H | 5.44880400 | -1.67601800 | 0.20276500 |
| H | 4.90671900 | -2.05155100 | -1.43566400 |
| H | 4.27991500 | -3.00664900 | -0.05565100 |
| H | 2.66240300 | 0.89888600 | -0.17101300 |
| H | 1.46996300 | 3.12232800 | -1.15341600 |
| H | 1.33273900 | 3.98650800 | 0.36979400 |

| | | | |
|---|---|---|---|
| H | 3.08301400 | 2.24644000 | 0.53040300 |

**7_H**

E (M06-SMD/BS1) = -775.513721 au

H (M06-SMD/BS1) = -775.303612 au

G (M06-SMD/BS1) = -775.357804 au

E (M06-SMD/BS2//M06-SMD/BS1) = -775.7869354 au

| | | | |
|---|---|---|---|
| C | -0.94661400 | 2.44939900 | -0.07484300 |
| C | -0.45855600 | 1.15023500 | -0.01928400 |
| C | -1.41178300 | 0.07889900 | 0.01361300 |
| C | -2.80422800 | 0.35305100 | 0.00116200 |
| C | -3.25436800 | 1.69170000 | -0.04960100 |
| C | -2.33151800 | 2.70688000 | -0.08816000 |
| H | -0.24756900 | 3.27632300 | -0.10541000 |
| C | -3.69202900 | -0.74724600 | 0.03680100 |
| H | -4.32520800 | 1.89087600 | -0.06073400 |
| H | -2.66378500 | 3.74320500 | -0.13123500 |
| C | -3.20151800 | -2.02683900 | 0.08084000 |
| C | -1.80758200 | -2.21318800 | 0.08844500 |
| H | -4.76501600 | -0.55590900 | 0.02785700 |
| H | -3.86009100 | -2.89124300 | 0.10874800 |
| H | -1.38197300 | -3.21599700 | 0.12228500 |
| N | -0.94858500 | -1.21111700 | 0.05599300 |
| N | 0.88660700 | 0.77520800 | 0.01744700 |
| C | 1.96510800 | 1.61571500 | 0.08105300 |
| C | 3.26464300 | 0.86177900 | 0.24259600 |
| H | 4.06317700 | 1.42224500 | -0.26637200 |
| H | 3.50091600 | 0.90783000 | 1.31807300 |
| C | 3.15896200 | -0.58587300 | -0.21179300 |
| H | 3.39037500 | -0.70015200 | -1.28230800 |
| O | 1.90698600 | 2.84877400 | 0.06405900 |
| Pd | 1.23466800 | -1.18157500 | -0.03775200 |
| H | 3.78929400 | -1.27062500 | 0.37414800 |

**17_H**

E (M06-SMD/BS1) = -1321.266785 au

H (M06-SMD/BS1) = -1320.907204 au

G (M06-SMD/BS1) = -1320.992877 au

E (M06-SMD/BS2//M06-SMD/BS1) = -1608.1543903 au

| | | | |
|---|---|---|---|
| C | 5.15669400 | 0.54958500 | 0.85407900 |
| C | 3.84258200 | 0.39528000 | 0.43041000 |

| | | | |
|---|---|---|---|
| C | 3.43279200 | -0.90792100 | -0.01107700 |
| C | 4.34683700 | -1.99404100 | -0.00353300 |
| C | 5.67403900 | -1.78792200 | 0.43619500 |
| C | 6.05420900 | -0.53620800 | 0.85302400 |
| H | 5.49132400 | 1.52315700 | 1.19302500 |
| C | 3.87637900 | -3.25284900 | -0.44440400 |
| H | 6.36937200 | -2.62642600 | 0.43539300 |
| H | 7.07431300 | -0.36401600 | 1.19403200 |
| C | 2.57654400 | -3.39701900 | -0.85760800 |
| C | 1.73541900 | -2.27017300 | -0.83552400 |
| H | 4.56374900 | -4.09885900 | -0.44696600 |
| H | 2.18763700 | -4.35299000 | -1.19900000 |
| H | 0.69432800 | -2.34905100 | -1.15534700 |
| N | 2.14010300 | -1.07901600 | -0.43596600 |
| N | 2.85734100 | 1.38157900 | 0.40013300 |
| C | 2.99453800 | 2.67509500 | 0.81425000 |
| C | 1.68456400 | 3.43199500 | 0.76895900 |
| H | 1.89928100 | 4.48794300 | 0.54367000 |
| H | 1.30659900 | 3.41858300 | 1.80464200 |
| C | 0.65556800 | 2.81131000 | -0.16991100 |
| H | 0.72167000 | 3.22779500 | -1.18784600 |
| O | 4.03740600 | 3.19008900 | 1.23604400 |
| Pd | 1.10162300 | 0.84340600 | -0.37034400 |
| C | -5.85454400 | 0.51783900 | 0.53722800 |
| C | -4.65605500 | -0.19415800 | 0.57565700 |
| C | -3.58187400 | 0.29490700 | -0.14872300 |
| C | -3.62611100 | 1.44859000 | -0.90521200 |
| C | -4.83624700 | 2.14377900 | -0.92817500 |
| C | -5.94070500 | 1.68314800 | -0.21363000 |
| H | -6.70558000 | 0.14214500 | 1.10299800 |
| H | -2.75949000 | 1.80336500 | -1.45816300 |
| H | -4.90878300 | 3.05799100 | -1.51442800 |
| H | -6.87462400 | 2.24044900 | -0.24337300 |
| I | -1.83454600 | -0.91700900 | 0.05539500 |
| C | -4.50064300 | -1.44499000 | 1.36647900 |
| O | -5.41893200 | -1.92877500 | 2.01859000 |
| O | -3.30962800 | -1.98139600 | 1.30683600 |
| O | -0.82901600 | 0.42170800 | -1.21676400 |
| C | -0.79802600 | 0.03119100 | -2.58826200 |
| H | -0.37444900 | 0.87369800 | -3.15038600 |
| H | -1.81758400 | -0.16771500 | -2.95036300 |

| | | | |
|---|---|---|---|
| H | -0.17138500 | -0.85900800 | -2.74723900 |
| H | -0.37661600 | 2.93215700 | 0.19108900 |

**$TS_{17\text{-}18\_H}$**

E (M06-SMD/BS1) = -1321.249538 au

H (M06-SMD/BS1) = -1320.891296 au

G (M06-SMD/BS1) = -1320.974572 au

E (M06-SMD/BS2//M06-SMD/BS1) = -1608.127506 au

| | | | |
|---|---|---|---|
| C | -4.56963600 | 1.18699600 | -1.02916700 |
| C | -3.42735000 | 0.70180400 | -0.40548100 |
| C | -3.38819600 | -0.68993500 | -0.06572700 |
| C | -4.48806700 | -1.53457700 | -0.37070600 |
| C | -5.63098900 | -0.99626900 | -1.00386500 |
| C | -5.65585300 | 0.33910500 | -1.31991500 |
| H | -4.62111600 | 2.23654300 | -1.29390800 |
| C | -4.38664800 | -2.90069500 | -0.02252400 |
| H | -6.47006300 | -1.65323100 | -1.22914400 |
| H | -6.53056100 | 0.76700600 | -1.80767500 |
| C | -3.25220000 | -3.37169200 | 0.58635000 |
| C | -2.20921500 | -2.46890300 | 0.85750000 |
| H | -5.22110300 | -3.56429400 | -0.24882900 |
| H | -3.14262200 | -4.41705200 | 0.86338400 |
| H | -1.29704800 | -2.81459600 | 1.34234200 |
| N | -2.26831100 | -1.18510600 | 0.55246000 |
| N | -2.28994600 | 1.43816500 | -0.07693500 |
| C | -2.06274300 | 2.74921600 | -0.38384200 |
| C | -0.65419500 | 3.17588500 | -0.03193400 |
| H | -0.65035000 | 4.25373900 | 0.19122700 |
| H | -0.05760900 | 3.05173300 | -0.95154300 |
| C | -0.05669700 | 2.35401300 | 1.09048000 |
| H | -0.44456600 | 2.63474400 | 2.08132500 |
| O | -2.86698500 | 3.51559200 | -0.92416300 |
| Pd | -0.82388500 | 0.47131600 | 0.90102800 |
| C | 5.66456200 | -0.16315100 | -0.31174300 |
| C | 4.33814400 | -0.30268100 | -0.72053700 |
| C | 3.33725800 | -0.04194000 | 0.20655100 |
| C | 3.59272500 | 0.35720800 | 1.50410200 |
| C | 4.92886200 | 0.49398600 | 1.88401700 |
| C | 5.95957500 | 0.23577600 | 0.98444200 |
| H | 6.45105700 | -0.37533100 | -1.03457000 |
| H | 2.78225500 | 0.54419800 | 2.20198700 |

| | | | |
|---|---|---|---|
| H | 5.15632200 | 0.80644300 | 2.90176200 |
| H | 6.99593500 | 0.34578400 | 1.29702000 |
| I | 1.35170500 | -0.27010800 | -0.63077900 |
| C | 4.01152200 | -0.73175400 | -2.10828900 |
| O | 4.88511800 | -0.97940500 | -2.93614500 |
| O | 2.74139600 | -0.82697000 | -2.37532000 |
| O | 0.81834000 | -0.26025600 | 1.89690600 |
| C | 0.80094400 | -1.57170200 | 2.38459000 |
| H | 1.69522700 | -1.72461400 | 3.01309800 |
| H | 0.83817100 | -2.33137900 | 1.58291100 |
| H | -0.09197500 | -1.77142800 | 3.00085200 |
| H | 1.04041000 | 2.35966800 | 1.10906000 |

**18_H**

E (M06-SMD/BS1) = -1321.261937 au

H (M06-SMD/BS1) = -1320.903361 au

G (M06-SMD/BS1) = -1320.990104 au

E (M06-SMD/BS2//M06-SMD/BS1) = -1608.13410033 au

| | | | |
|---|---|---|---|
| C | -3.68710500 | -1.86261400 | -1.01264700 |
| C | -2.85688900 | -0.80626200 | -0.66341100 |
| C | -3.11951900 | -0.12484000 | 0.56710900 |
| C | -4.21526700 | -0.50284300 | 1.38327200 |
| C | -5.04541600 | -1.57372900 | 0.98059300 |
| C | -4.77079500 | -2.23350600 | -0.19188500 |
| H | -3.50328500 | -2.40083400 | -1.93554800 |
| C | -4.41795700 | 0.21708800 | 2.58251400 |
| H | -5.88438700 | -1.86043300 | 1.61305700 |
| H | -5.39832500 | -3.06517600 | -0.50795100 |
| C | -3.56785400 | 1.23837100 | 2.92864800 |
| C | -2.49908300 | 1.54509000 | 2.06940800 |
| H | -5.25599500 | -0.05571800 | 3.22350000 |
| H | -3.70089600 | 1.80541200 | 3.84622700 |
| H | -1.79291600 | 2.34104400 | 2.31014500 |
| N | -2.28699000 | 0.89404400 | 0.94108400 |
| N | -1.76604900 | -0.33748700 | -1.40230700 |
| C | -1.39888900 | -0.75263900 | -2.65156800 |
| C | -0.05875700 | -0.19230500 | -3.07773100 |
| H | -0.09787200 | 0.01326200 | -4.15839400 |
| H | 0.67490200 | -1.00287300 | -2.94801500 |
| C | 0.36418900 | 1.03821000 | -2.31638900 |
| H | -0.10727900 | 1.96837000 | -2.67377700 |

| | | | |
|---|---|---|---|
| O | -2.04868300 | -1.51108200 | -3.37304900 |
| Pd | -0.63015200 | 1.06663500 | -0.50719200 |
| C | 5.33327100 | -0.18774700 | 0.47484500 |
| C | 4.04657400 | -0.72067900 | 0.56608000 |
| C | 2.98194600 | 0.10937000 | 0.23842400 |
| C | 3.13449900 | 1.43065100 | -0.14176600 |
| C | 4.43335600 | 1.93435000 | -0.22015200 |
| C | 5.52838200 | 1.12827900 | 0.07818800 |
| H | 6.17311800 | -0.83269500 | 0.72888600 |
| H | 2.26956200 | 2.06474700 | -0.33068900 |
| H | 4.58003400 | 2.97243100 | -0.51386100 |
| H | 6.53680800 | 1.53176200 | 0.01107400 |
| I | 1.03972200 | -0.82206500 | 0.36280900 |
| C | 3.83916800 | -2.12923700 | 1.03302700 |
| O | 4.80833800 | -2.84797800 | 1.29833700 |
| O | 2.61269600 | -2.50240100 | 1.14104100 |
| O | 0.31469300 | 2.58931300 | 0.41167100 |
| C | -0.30904000 | 3.75176800 | -0.03949700 |
| H | 0.01799900 | 4.05063000 | -1.05393500 |
| H | -0.07330800 | 4.58585700 | 0.64512900 |
| H | -1.41630700 | 3.66833300 | -0.05775800 |
| H | 1.44590400 | 1.16074300 | -2.21394400 |

**$TS_{18\text{-}19\_H}$**

E (M06-SMD/BS1) = -1321.259296 au

H (M06-SMD/BS1) = -1320.901324 au

G (M06-SMD/BS1) = -1320.98611 au

E (M06-SMD/BS2//M06-SMD/BS1) = -1608.130922 au

| | | | |
|---|---|---|---|
| C | -3.78078500 | -2.28120600 | -0.10208000 |
| C | -3.00259500 | -1.14131700 | -0.19669400 |
| C | -3.50270000 | 0.06860600 | 0.36072500 |
| C | -4.78420700 | 0.11736300 | 0.96296300 |
| C | -5.55707400 | -1.06532500 | 1.02631600 |
| C | -5.05267800 | -2.23213800 | 0.50686800 |
| H | -3.41022400 | -3.21987600 | -0.50079500 |
| C | -5.21269200 | 1.35909900 | 1.48308500 |
| H | -6.53689600 | -1.03113800 | 1.50033300 |
| H | -5.63697300 | -3.14887700 | 0.56365000 |
| C | -4.39505300 | 2.46038400 | 1.40244300 |
| C | -3.13441200 | 2.32753000 | 0.79787900 |
| H | -6.19582500 | 1.42529500 | 1.94822600 |

| | | | |
|---|---|---|---|
| H | -4.69800800 | 3.42589800 | 1.79849800 |
| H | -2.45424900 | 3.17642700 | 0.72901800 |
| N | -2.71046400 | 1.18168500 | 0.29584400 |
| N | -1.74807800 | -1.04283200 | -0.81357500 |
| C | -1.43100700 | -1.73200000 | -1.94768700 |
| C | -0.16051900 | -1.21695600 | -2.58697300 |
| H | -0.26739600 | -1.30620800 | -3.67956400 |
| H | 0.67766700 | -1.86585900 | -2.29801100 |
| C | 0.09399800 | 0.21280600 | -2.23497000 |
| H | -0.59218800 | 0.93470100 | -2.69739400 |
| O | -2.09170600 | -2.65096400 | -2.43157400 |
| Pd | -0.68569200 | 0.62508600 | -0.30302500 |
| C | 5.54419200 | 0.47722500 | 0.12497600 |
| C | 4.37959100 | -0.21703900 | 0.46021500 |
| C | 3.16788600 | 0.33839200 | 0.06307300 |
| C | 3.07701000 | 1.53940500 | -0.62010300 |
| C | 4.25762600 | 2.20873400 | -0.93837200 |
| C | 5.48974700 | 1.67652300 | -0.57181300 |
| H | 6.49615300 | 0.04754100 | 0.43254500 |
| H | 2.11041400 | 1.96495000 | -0.87522400 |
| H | 4.20225600 | 3.15477300 | -1.47419500 |
| H | 6.40887500 | 2.20231300 | -0.82335800 |
| I | 1.39416500 | -0.78233400 | 0.56264000 |
| C | 4.47349400 | -1.49852500 | 1.24701400 |
| O | 5.59189500 | -1.94612300 | 1.54229000 |
| O | 3.36025200 | -2.03446100 | 1.56688600 |
| O | 0.13277100 | 2.37372000 | 0.23801600 |
| C | -0.10551300 | 3.35399000 | -0.72815900 |
| H | 0.52681100 | 3.23492400 | -1.62999000 |
| H | 0.12266600 | 4.34396800 | -0.29741800 |
| H | -1.15856600 | 3.38672700 | -1.07112800 |
| H | 1.13485100 | 0.54150500 | -2.25900300 |

**19_H**

E (M06-SMD/BS1) = -1321.263357 au
H (M06-SMD/BS1) = -1320.905492 au
G (M06-SMD/BS1) = -1320.987716 au
E (M06-SMD/BS2//M06-SMD/BS1) = -1608.131926 au

| | | | |
|---|---|---|---|
| C | -4.11675400 | -1.79550600 | 0.60382100 |
| C | -3.08358900 | -0.94895000 | 0.26270600 |
| C | -3.38291500 | 0.40447700 | -0.03859100 |

| | | | |
|---|---|---|---|
| C | -4.71353100 | 0.88295200 | -0.03402900 |
| C | -5.74948000 | -0.01444000 | 0.31889000 |
| C | -5.44573100 | -1.31636500 | 0.63357400 |
| H | -3.90615700 | -2.83350900 | 0.84596800 |
| C | -4.91820500 | 2.23405700 | -0.39275500 |
| H | -6.77596900 | 0.34783000 | 0.33912900 |
| H | -6.24099300 | -2.00577500 | 0.91078000 |
| C | -3.84949300 | 3.03273100 | -0.72439000 |
| C | -2.55190300 | 2.49779100 | -0.68323700 |
| H | -5.93314900 | 2.62970400 | -0.40399300 |
| H | -3.98159000 | 4.07398500 | -1.00490500 |
| H | -1.67553100 | 3.10545600 | -0.90191200 |
| N | -2.34087400 | 1.23530300 | -0.35480200 |
| N | -1.72946000 | -1.29531500 | 0.09330000 |
| C | -1.41975200 | -2.28519600 | -0.81137100 |
| C | -0.13010800 | -1.98598200 | -1.54350800 |
| H | -0.18676000 | -2.43768700 | -2.54661000 |
| H | 0.73397800 | -2.43569200 | -1.03524200 |
| C | 0.01148500 | -0.50637400 | -1.69138200 |
| H | -0.72543300 | -0.02505700 | -2.34255100 |
| O | -2.13227600 | -3.25364500 | -1.04919300 |
| Pd | -0.53062400 | 0.37475700 | 0.15629500 |
| C | 4.72230800 | 0.71395900 | -1.16153100 |
| C | 3.73122900 | -0.11306800 | -0.62020000 |
| C | 3.16963200 | 0.29282900 | 0.58804500 |
| C | 3.55824400 | 1.45101200 | 1.25547800 |
| C | 4.56815000 | 2.23369100 | 0.70447100 |
| C | 5.14867100 | 1.86193900 | -0.50559400 |
| H | 5.15978300 | 0.43328100 | -2.11908900 |
| H | 3.07227700 | 1.74682600 | 2.18338000 |
| H | 4.88928000 | 3.13712700 | 1.21977300 |
| H | 5.93209000 | 2.47655200 | -0.94583600 |
| I | 1.53938800 | -0.79765100 | 1.45157400 |
| C | 3.33027300 | -1.35795800 | -1.39992200 |
| O | 3.07233300 | -1.17160200 | -2.61333600 |
| O | 3.30474500 | -2.44238000 | -0.77255300 |
| O | 0.41839600 | 2.13658600 | 0.23895300 |
| C | 0.98220800 | 2.56299900 | -0.96403300 |
| H | 1.84066600 | 1.94262200 | -1.28649300 |
| H | 1.36258600 | 3.58959100 | -0.82574200 |
| H | 0.25482100 | 2.59507600 | -1.79967400 |

| | | | |
|---|---|---|---|
| H | 1.02355700 | -0.10282900 | -1.78908100 |

**21_H**

E (M06-SMD/BS1) = -1436.931147 au
H (M06-SMD/BS1) = -1436.514214 au
G (M06-SMD/BS1) = -1436.61058 au
E (M06-SMD/BS2//M06-SMD/BS1) = -1723.850027 au

| | | | |
|---|---|---|---|
| C | 4.30652500 | 0.30888100 | 1.76622800 |
| C | 3.22681800 | 0.15192900 | 0.92282400 |
| C | 3.43469200 | -0.44589000 | -0.34666500 |
| C | 4.72396700 | -0.84498800 | -0.76900600 |
| C | 5.80959700 | -0.66608600 | 0.12070000 |
| C | 5.59321500 | -0.10726800 | 1.35649500 |
| H | 4.16547700 | 0.75512700 | 2.74659700 |
| C | 4.84121200 | -1.39954900 | -2.06299100 |
| H | 6.80337500 | -0.98352200 | -0.19121100 |
| H | 6.42612400 | 0.02454700 | 2.04463500 |
| C | 3.72924800 | -1.53918700 | -2.85843900 |
| C | 2.47575400 | -1.14314600 | -2.36453500 |
| H | 5.82259900 | -1.71346500 | -2.41683300 |
| H | 3.79174600 | -1.96294700 | -3.85687600 |
| H | 1.56875400 | -1.27488800 | -2.95123400 |
| N | 2.34476200 | -0.61481400 | -1.16040300 |
| N | 1.90815800 | 0.58983400 | 1.14540400 |
| C | 1.68963100 | 1.91312100 | 1.43222400 |
| C | 0.37526600 | 2.39632800 | 0.85825900 |
| H | 0.46329500 | 3.47316300 | 0.65246100 |
| H | -0.44500600 | 2.26411200 | 1.57572800 |
| C | 0.10843100 | 1.68378100 | -0.42504100 |
| H | 0.80921400 | 1.87689900 | -1.24299000 |
| O | 2.48243900 | 2.64128800 | 2.02045600 |
| Pd | 0.58854900 | -0.39814700 | -0.07835300 |
| C | -4.78630100 | -0.38615000 | -0.97803700 |
| C | -3.72808500 | -0.02464100 | -0.13585900 |
| C | -3.07427300 | -1.05972600 | 0.53092200 |
| C | -3.42663200 | -2.39721300 | 0.37183500 |
| C | -4.50037900 | -2.71885600 | -0.45202000 |
| C | -5.18309000 | -1.70855700 | -1.12335500 |
| H | -5.29573700 | 0.40385700 | -1.52798800 |
| H | -2.86740200 | -3.18120700 | 0.87890700 |
| H | -4.79098600 | -3.76074600 | -0.57335000 |

| | | | |
|---|---|---|---|
| H | -6.01946900 | -1.95392400 | -1.77545300 |
| I | -1.37149700 | -0.70647400 | 1.78761600 |
| C | -3.33409600 | 1.44004500 | -0.07449600 |
| O | -3.29585600 | 2.02235300 | -1.19576500 |
| O | -3.07235900 | 1.94520500 | 1.03594400 |
| O | -0.49656400 | -1.50422700 | -1.34427100 |
| C | -1.05670500 | -0.81025200 | -2.41711300 |
| H | -1.85586700 | -0.10777100 | -2.11402300 |
| H | -1.51671200 | -1.54311000 | -3.10298000 |
| H | -0.31212900 | -0.23834400 | -3.00761800 |
| H | -0.92168900 | 1.50615200 | -0.73610600 |
| C | -1.13188500 | 3.56161400 | -2.84617700 |
| H | -1.06057000 | 2.48435000 | -3.07647800 |
| H | -0.21000200 | 4.04549200 | -3.19114000 |
| H | -1.97325000 | 3.97573500 | -3.42518900 |
| O | -1.26871200 | 3.80726700 | -1.46480700 |
| H | -2.08516500 | 3.32301400 | -1.19026200 |

**$TS_{21\text{-}22\_H}$**

E (M06-SMD/BS1) = -1436.930447 au

H (M06-SMD/BS1) = -1436.514615 au

G (M06-SMD/BS1) = -1436.60802 au

E (M06-SMD/BS2//M06-SMD/BS1) = -1723.84781 au

| | | | |
|---|---|---|---|
| C | 4.34754400 | 0.97243900 | 1.41540500 |
| C | 3.26394600 | 0.49108900 | 0.70843000 |
| C | 3.47751900 | -0.54408600 | -0.23941800 |
| C | 4.77260700 | -1.05362900 | -0.49287400 |
| C | 5.86020600 | -0.53322300 | 0.24746700 |
| C | 5.63895100 | 0.45079500 | 1.17952400 |
| H | 4.20541500 | 1.75516800 | 2.15420500 |
| C | 4.89457400 | -2.05910200 | -1.47783600 |
| H | 6.85807300 | -0.93040700 | 0.06802600 |
| H | 6.47267500 | 0.84747300 | 1.75610800 |
| C | 3.78149600 | -2.50969800 | -2.14554800 |
| C | 2.52395200 | -1.97313100 | -1.82504000 |
| H | 5.88022000 | -2.46850800 | -1.69672300 |
| H | 3.84679100 | -3.28204900 | -2.90712200 |
| H | 1.61676300 | -2.33180800 | -2.30747700 |
| N | 2.38685500 | -1.02628100 | -0.91335000 |
| N | 1.93896000 | 0.95361800 | 0.76377800 |
| C | 1.68153400 | 2.29168000 | 0.69496800 |

| | | | |
|---|---|---|---|
| C | 0.33069300 | 2.57875600 | 0.06202100 |
| H | 0.40757700 | 3.56243900 | -0.42391000 |
| H | -0.46070000 | 2.64211800 | 0.82021100 |
| C | 0.02753400 | 1.55602300 | -0.96923800 |
| H | 0.70751500 | 1.45687800 | -1.81700200 |
| O | 2.45631700 | 3.18201400 | 1.03392700 |
| Pd | 0.62002200 | -0.43487000 | 0.00641400 |
| C | -4.73720000 | -0.91985200 | -0.94645900 |
| C | -3.71794000 | -0.25614700 | -0.25372700 |
| C | -3.05219400 | -0.97893600 | 0.73489600 |
| C | -3.35969600 | -2.30449400 | 1.02936200 |
| C | -4.39702500 | -2.92610600 | 0.34170000 |
| C | -5.08858400 | -2.22845400 | -0.64463600 |
| H | -5.25255800 | -0.38208200 | -1.74122600 |
| H | -2.79234400 | -2.84941100 | 1.78172300 |
| H | -4.65211400 | -3.95860300 | 0.57322400 |
| H | -5.89588400 | -2.71175500 | -1.19203100 |
| I | -1.40064700 | -0.15169700 | 1.82378500 |
| C | -3.36282000 | 1.15396800 | -0.68378000 |
| O | -3.23637000 | 1.30344400 | -1.93390600 |
| O | -3.21289500 | 2.02919600 | 0.19260800 |
| O | -0.42288200 | -1.95189300 | -0.77004000 |
| C | -0.97964800 | -1.67310700 | -2.01902500 |
| H | -1.74526800 | -0.87568200 | -1.98872200 |
| H | -1.47900100 | -2.58509500 | -2.39133900 |
| H | -0.22620600 | -1.38053400 | -2.77888100 |
| H | -0.97508700 | 1.15731800 | -1.09944800 |
| C | -1.63740800 | 4.33120700 | -1.93460300 |
| H | -2.33953600 | 4.86301400 | -2.59667400 |
| H | -0.73820600 | 4.95114000 | -1.83223400 |
| H | -2.11154000 | 4.22345800 | -0.94676600 |
| O | -1.26548900 | 3.08309100 | -2.47899700 |
| H | -2.05165900 | 2.48677700 | -2.38085800 |

**22_H**

E (M06-SMD/BS1) = -1437.004094 au

H (M06-SMD/BS1) = -1436.583534 au

G (M06-SMD/BS1) = -1436.675969 au

E (M06-SMD/BS2//M06-SMD/BS1) = -1723.916481 au

| | | | |
|---|---|---|---|
| C | 4.15968200 | 1.39150900 | 1.10265800 |
| C | 3.13091200 | 0.69886800 | 0.48559500 |

| | | | |
|---|---|---|---|
| C | 3.47028300 | -0.48413600 | -0.22785500 |
| C | 4.81531000 | -0.90275000 | -0.37758400 |
| C | 5.83576800 | -0.14887800 | 0.24566400 |
| C | 5.49778100 | 0.96092800 | 0.98066300 |
| H | 3.93845400 | 2.28027500 | 1.68312100 |
| C | 5.05626500 | -2.07350000 | -1.13176200 |
| H | 6.87062900 | -0.47262600 | 0.14420200 |
| H | 6.27487200 | 1.53559300 | 1.48232200 |
| C | 4.00661100 | -2.77194500 | -1.67526500 |
| C | 2.69583300 | -2.31904800 | -1.45057600 |
| H | 6.08324700 | -2.41211500 | -1.26612000 |
| H | 4.16321500 | -3.67658900 | -2.25677000 |
| H | 1.83140400 | -2.87161800 | -1.81525200 |
| N | 2.44645700 | -1.21993400 | -0.76189800 |
| N | 1.76589700 | 1.04069000 | 0.46709900 |
| C | 1.41313300 | 2.34776000 | 0.35845100 |
| C | -0.02707700 | 2.65334000 | 0.01947300 |
| H | -0.44699800 | 3.27848900 | 0.82221800 |
| C | -0.06778500 | 3.39710900 | -1.31094300 |
| O | 2.20175700 | 3.30264800 | 0.45349300 |
| Pd | 0.62670400 | -0.64459500 | 0.01876400 |
| C | -4.49093500 | -0.88070500 | -1.31409100 |
| C | -3.55900900 | -0.30865600 | -0.44436400 |
| C | -3.02463600 | -1.09587900 | 0.57616500 |
| C | -3.41002700 | -2.42010300 | 0.74375400 |
| C | -4.36179400 | -2.96496900 | -0.11488400 |
| C | -4.89897400 | -2.19774400 | -1.14365500 |
| H | -4.89639200 | -0.27556100 | -2.12401000 |
| H | -2.96130700 | -3.02863000 | 1.52643400 |
| H | -4.67053100 | -4.00013200 | 0.01843200 |
| H | -5.63295300 | -2.62787000 | -1.82205600 |
| I | -1.44061800 | -0.36805900 | 1.81859500 |
| C | -3.15831000 | 1.11223400 | -0.64613700 |
| O | -2.63284000 | 1.32277300 | -1.84376100 |
| O | -3.29666900 | 1.97846800 | 0.19792700 |
| O | -0.23013500 | -2.36616000 | -0.56292400 |
| C | -0.80116300 | -2.19257700 | -1.82201700 |
| H | -1.57023700 | -1.39465100 | -1.84919000 |
| H | -1.30591200 | -3.12780000 | -2.12646500 |
| H | -0.05548400 | -1.95824600 | -2.61027400 |
| H | 0.36568900 | 2.76603500 | -2.10073200 |

| | | | |
|---|---|---|---|
| C | -1.99660700 | 4.76410200 | -1.03535600 |
| H | -2.96810800 | 4.94765100 | -1.50546300 |
| H | -1.38139600 | 5.67350900 | -1.10696300 |
| H | -2.15635500 | 4.52033800 | 0.02432300 |
| O | -1.38528100 | 3.70304700 | -1.74670300 |
| H | -2.29875800 | 2.26907300 | -1.88070600 |
| H | -0.63066900 | 1.74517200 | -0.05899800 |
| H | 0.51925800 | 4.32694300 | -1.25498300 |

**1_OMe**

E (M06-SMD/BS1) = -763.2571087 au

H (M06-SMD/BS1) = -762.988392 au

G (M06-SMD/BS1) = -763.048036 au

E (M06-SMD/BS2//M06-SMD/BS1) = -763.5502595 au

| | | | |
|---|---|---|---|
| C | -0.30673300 | 1.47821200 | -0.52285700 |
| C | -0.65443000 | 0.15420000 | -0.35083500 |
| C | -2.01728200 | -0.17292700 | -0.04448100 |
| C | -2.96775600 | 0.87053400 | 0.10983100 |
| C | -2.56760700 | 2.21856000 | -0.04280100 |
| C | -1.26363400 | 2.50235300 | -0.35675000 |
| H | 0.70225400 | 1.75829300 | -0.80629900 |
| C | -4.29675900 | 0.49454700 | 0.41345200 |
| H | -3.30648800 | 3.00924200 | 0.08325300 |
| H | -0.94529000 | 3.53446500 | -0.49294400 |
| C | -4.61548600 | -0.83293400 | 0.53908600 |
| C | -3.59637200 | -1.79019900 | 0.35590600 |
| H | -5.04965400 | 1.27261600 | 0.54031600 |
| H | -5.62773600 | -1.15607900 | 0.77072300 |
| H | -3.83239600 | -2.85247500 | 0.44478400 |
| N | -2.34668700 | -1.48497800 | 0.07778400 |
| N | 0.19429300 | -0.94827100 | -0.49126300 |
| H | -0.31609400 | -1.83304300 | -0.50903300 |
| C | 1.54884400 | -1.12359000 | -0.41485700 |
| C | 2.44214500 | 0.06154200 | -0.19528900 |
| H | 2.46152800 | 0.67854400 | -1.10658200 |
| H | 2.03724800 | 0.69790400 | 0.60523500 |
| C | 3.85336500 | -0.35921000 | 0.14866200 |
| H | 4.29363800 | -0.95940000 | -0.66685000 |
| H | 3.86445400 | -0.98912000 | 1.05680900 |
| O | 2.00271600 | -2.26385800 | -0.51533500 |
| O | 4.60590500 | 0.81373500 | 0.35590500 |

| | | | |
|---|---|---|---|
| C | 5.94168000 | 0.51921400 | 0.68306000 |
| H | 6.46672500 | 1.46904000 | 0.83174700 |
| H | 6.00869500 | -0.07514500 | 1.61022000 |
| H | 6.44107100 | -0.04293100 | -0.12434700 |

**13_OMe**

E (M06-SMD/BS1) = -1347.967023 au

H (M06-SMD/BS1) = -1347.581493 au

G (M06-SMD/BS1) = -1347.671382 au

E (M06-SMD/BS2//M06-SMD/BS1) = -1348.469215 au

| | | | |
|---|---|---|---|
| C | 2.73830400 | 0.40750100 | 2.03487900 |
| C | 1.62439600 | 0.49171600 | 1.23720500 |
| C | 1.40252900 | 1.62875300 | 0.43627200 |
| C | 2.33319400 | 2.69459300 | 0.45320600 |
| C | 3.47226000 | 2.58722700 | 1.28470200 |
| C | 3.66801000 | 1.46840500 | 2.05772500 |
| H | 2.89758200 | -0.48083100 | 2.64560400 |
| C | 2.06222500 | 3.81225200 | -0.36873500 |
| H | 4.18647800 | 3.40924800 | 1.29864600 |
| H | 4.54474300 | 1.39039200 | 2.69636700 |
| C | 0.93008200 | 3.83085300 | -1.14487900 |
| C | 0.05276800 | 2.73353600 | -1.10983800 |
| H | 2.76074000 | 4.64798100 | -0.37420900 |
| H | 0.69310700 | 4.67440100 | -1.78707600 |
| H | -0.85408500 | 2.71118200 | -1.71247500 |
| N | 0.28178100 | 1.67535000 | -0.35082500 |
| N | 0.64011500 | -0.57299000 | 1.17735800 |
| H | 0.12744500 | -0.71321200 | 2.06145400 |
| C | 1.05739400 | -1.85642200 | 0.66933100 |
| C | 2.01061000 | -1.84606200 | -0.48173300 |
| H | 3.01416000 | -2.06712800 | -0.08561000 |
| H | 2.05640600 | -0.85724500 | -0.95659300 |
| C | 1.58576400 | -2.89486700 | -1.50850500 |
| H | 1.66116300 | -3.90592800 | -1.07357500 |
| H | 0.53648300 | -2.72449000 | -1.79024900 |
| O | 0.57578000 | -2.85975800 | 1.14768400 |
| Pd | -0.92090000 | 0.05094300 | -0.12216500 |
| O | -2.32394400 | 0.74065400 | -1.42148900 |
| C | -3.31298000 | 1.35757000 | -0.84721800 |
| O | -3.41461900 | 1.53149600 | 0.37141900 |
| O | -2.08126900 | -1.59749800 | 0.17115900 |

| | | | |
|---|---|---|---|
| C | -2.34264500 | -1.87315000 | 1.41219200 |
| O | -1.96693200 | -1.19357200 | 2.37643200 |
| C | -4.34107800 | 1.87487700 | -1.81710900 |
| H | -3.88535200 | 2.63924500 | -2.45974800 |
| H | -5.19117200 | 2.31106900 | -1.28448800 |
| H | -4.68929900 | 1.06605600 | -2.47038600 |
| C | -3.12039500 | -3.14518900 | 1.60655500 |
| H | -3.61285200 | -3.14945000 | 2.58392200 |
| H | -2.41717700 | -3.98891600 | 1.56818000 |
| H | -3.85882200 | -3.29008500 | 0.81092200 |
| O | 2.33539500 | -2.81092800 | -2.69484200 |
| C | 3.66044000 | -3.27386800 | -2.55396200 |
| H | 3.68414400 | -4.28227700 | -2.10871100 |
| H | 4.27547400 | -2.60155100 | -1.93479100 |
| H | 4.09844500 | -3.31783900 | -3.55677200 |

**$TS_{13\text{-}14\_OMe}$**

E (M06-SMD/BS1) = -1347.954692 au

H (M06-SMD/BS1) = -1347.574858 au

G (M06-SMD/BS1) = -1347.663598 au

E (M06-SMD/BS2//M06-SMD/BS1) = -1348.457651 au

| | | | |
|---|---|---|---|
| C | -2.09526400 | 0.75160900 | -2.19912500 |
| C | -1.15750200 | 0.59412400 | -1.20606800 |
| C | -0.66332700 | 1.73318500 | -0.53266700 |
| C | -1.15795000 | 3.02325100 | -0.83439000 |
| C | -2.12987500 | 3.15089000 | -1.85453400 |
| C | -2.57611400 | 2.03821000 | -2.52576200 |
| H | -2.47425500 | -0.12686000 | -2.72050000 |
| C | -0.63409300 | 4.11123800 | -0.09927200 |
| H | -2.50529300 | 4.14334200 | -2.09934400 |
| H | -3.31632800 | 2.13921300 | -3.31671700 |
| C | 0.32236900 | 3.89281600 | 0.86151600 |
| C | 0.78792300 | 2.58599500 | 1.08673100 |
| H | -0.99753200 | 5.11655900 | -0.30864500 |
| H | 0.74238300 | 4.70904800 | 1.44259000 |
| H | 1.56729700 | 2.37764500 | 1.81802900 |
| N | 0.31211400 | 1.55114600 | 0.41567900 |
| N | -0.66414000 | -0.68529000 | -0.78666900 |
| H | 0.02652100 | -1.41875900 | -1.54912800 |
| C | -1.51031100 | -1.50924600 | -0.00542500 |
| C | -2.75812700 | -0.90719800 | 0.57295600 |

| | | | |
|---|---|---|---|
| H | -3.45828100 | -0.69726700 | -0.24971600 |
| H | -2.52042300 | 0.06760000 | 1.02718600 |
| C | -3.39426800 | -1.81920700 | 1.60939100 |
| H | -3.71233300 | -2.76813900 | 1.14409600 |
| H | -2.66456200 | -2.06007400 | 2.39338300 |
| O | -1.19580600 | -2.67322500 | 0.19078000 |
| Pd | 1.06621000 | -0.32682700 | 0.32977600 |
| O | 2.71431000 | 0.11230600 | 1.45013300 |
| C | 3.69709800 | 0.62570700 | 0.77505100 |
| O | 3.67472900 | 0.83431200 | -0.44311700 |
| O | 1.89949800 | -2.17195200 | -0.10420500 |
| C | 1.65268500 | -2.69364400 | -1.23401800 |
| O | 0.78831000 | -2.25621000 | -2.05951200 |
| C | 4.88820200 | 0.98843900 | 1.62081500 |
| H | 4.63437900 | 1.85627700 | 2.24411700 |
| H | 5.74700300 | 1.24155200 | 0.99200900 |
| H | 5.14986600 | 0.16618700 | 2.29653600 |
| C | 2.40336900 | -3.92809000 | -1.60886500 |
| H | 1.76534000 | -4.79670000 | -1.39758800 |
| H | 3.32659900 | -4.02162700 | -1.03069800 |
| H | 2.62090500 | -3.92765300 | -2.68184900 |
| O | -4.48463000 | -1.20569100 | 2.25990400 |
| C | -5.64003200 | -1.11674400 | 1.45803500 |
| H | -5.53165800 | -0.38680300 | 0.63989000 |
| H | -6.46186300 | -0.78903500 | 2.10421600 |
| H | -5.89922100 | -2.09576600 | 1.02126400 |

**15_OMe**
E (M06-SMD/BS1) = -1118.968347 au
H (M06-SMD/BS1) = -1118.652874 au
G (M06-SMD/BS1) = -1118.727167 au
E (M06-SMD/BS2//M06-SMD/BS1) = -1119.381355 au

| | | | |
|---|---|---|---|
| C | 3.03222400 | 1.94152700 | -0.26437400 |
| C | 1.94543800 | 1.10090400 | -0.07012200 |
| C | 2.20373400 | -0.29519300 | -0.00233900 |
| C | 3.50939300 | -0.82601500 | -0.13084700 |
| C | 4.58673000 | 0.06681500 | -0.32676400 |
| C | 4.33477900 | 1.41514200 | -0.38898000 |
| H | 2.88016500 | 3.01176800 | -0.32153300 |
| C | 3.65748900 | -2.22936800 | -0.05651300 |
| H | 5.59502600 | -0.33190200 | -0.42545100 |

| | | | |
|---|---|---|---|
| H | 5.15702200 | 2.11273300 | -0.53982300 |
| C | 2.56090600 | -3.03402300 | 0.13347200 |
| C | 1.29307200 | -2.44457500 | 0.25529400 |
| H | 4.65416600 | -2.65847800 | -0.15362400 |
| H | 2.64883200 | -4.11528800 | 0.19235600 |
| H | 0.39327500 | -3.03758900 | 0.40920300 |
| N | 1.13522200 | -1.13334400 | 0.19089900 |
| N | 0.59293500 | 1.44910600 | 0.06673700 |
| C | 0.12516900 | 2.72561300 | 0.04829800 |
| C | -1.36590400 | 2.91092700 | 0.25055900 |
| H | -1.66735200 | 3.79193200 | -0.32961700 |
| H | -1.52582400 | 3.16187500 | 1.31041300 |
| C | -2.26108600 | 1.75656300 | -0.12568000 |
| H | -2.11671500 | 1.42979400 | -1.16864000 |
| H | -2.09754300 | 0.87118600 | 0.60088200 |
| O | 0.83172100 | 3.73093400 | -0.08439100 |
| Pd | -0.60776800 | -0.14629100 | 0.32811700 |
| O | -1.70525700 | -1.84624300 | 0.66590900 |
| C | -2.40171100 | -2.24062200 | -0.35491300 |
| O | -2.44873300 | -1.64378100 | -1.43740400 |
| C | -3.15600600 | -3.52146500 | -0.12148200 |
| H | -2.44219600 | -4.34635400 | 0.00287000 |
| H | -3.81690300 | -3.74332400 | -0.96469400 |
| H | -3.74300200 | -3.45795000 | 0.80254200 |
| O | -3.58888100 | 2.09657500 | 0.10973000 |
| C | -4.49103200 | 1.10384400 | -0.34213200 |
| H | -4.32975600 | 0.15421100 | 0.19424200 |
| H | -4.37725900 | 0.92930100 | -1.42319500 |
| H | -5.50168200 | 1.46683500 | -0.13440300 |

**$TS_{15\text{-}16\_OMe}$**

E (M06-SMD/BS1) = -1118.93653 au

H (M06-SMD/BS1) = -1118.62742 au

G (M06-SMD/BS1) = -1118.695884 au

E (M06-SMD/BS2//M06-SMD/BS1) = -1119.349105 au

| | | | |
|---|---|---|---|
| C | -2.96634700 | 2.04752200 | -0.19305100 |
| C | -1.95074600 | 1.10946700 | -0.07722400 |
| C | -2.32090100 | -0.26528600 | -0.00562900 |
| C | -3.67754800 | -0.66825500 | -0.05216800 |
| C | -4.68125400 | 0.31974200 | -0.17102000 |
| C | -4.31622700 | 1.64195800 | -0.23832300 |

| | | | |
|---|---|---|---|
| H | -2.71596300 | 3.10060500 | -0.25029400 |
| C | -3.95164800 | -2.05283800 | 0.02310400 |
| H | -5.72554700 | 0.01323200 | -0.20687600 |
| H | -5.08198500 | 2.41088100 | -0.32978600 |
| C | -2.92382800 | -2.95545600 | 0.13844400 |
| C | -1.60214900 | -2.48066300 | 0.17965100 |
| H | -4.98824200 | -2.38685300 | -0.01178600 |
| H | -3.10773500 | -4.02480100 | 0.19850600 |
| H | -0.75857600 | -3.16337100 | 0.27049100 |
| N | -1.31774600 | -1.19205600 | 0.11061000 |
| N | -0.57505600 | 1.34883200 | -0.01990900 |
| C | 0.01872900 | 2.56206900 | -0.12006700 |
| C | 1.53016700 | 2.50754100 | -0.08707700 |
| C | 2.08183700 | 1.28417100 | 0.61776200 |
| H | 1.66732700 | 1.19949100 | 1.63198900 |
| O | -0.56612600 | 3.64340300 | -0.26119100 |
| Pd | 0.55629400 | -0.30270700 | 0.16914600 |
| O | 1.64425500 | -2.08878200 | 0.27086200 |
| C | 2.75865500 | -1.97409600 | -0.32566500 |
| O | 3.16572200 | -0.86786900 | -0.79204100 |
| C | 3.62946000 | -3.17691500 | -0.47605400 |
| H | 4.56486400 | -3.01093200 | 0.07233100 |
| H | 3.88993500 | -3.31020400 | -1.53246100 |
| H | 3.13823200 | -4.07717500 | -0.09797000 |
| H | 2.30649100 | 0.07282300 | -0.27796000 |
| H | 1.87000400 | 2.55057900 | -1.13364700 |
| H | 1.89889300 | 3.42239300 | 0.40086500 |
| O | 3.46937100 | 1.31452000 | 0.83037400 |
| C | 4.28007400 | 1.80582100 | -0.22354000 |
| H | 4.03948600 | 1.33907100 | -1.18839800 |
| H | 5.31257400 | 1.55514200 | 0.03972200 |
| H | 4.19495300 | 2.89734800 | -0.31923300 |

**7_OMe**

E (M06-SMD/BS1) = -889.9783836 au

H (M06-SMD/BS1) = -889.732716 au

G (M06-SMD/BS1) = -889.793292 au

E (M06-SMD/BS2//M06-SMD/BS1) = -890.2963307 au

| | | | |
|---|---|---|---|
| C | 1.95750600 | 2.25209800 | 0.14518800 |
| C | 1.17420400 | 1.10892000 | 0.05417700 |
| C | 1.84261000 | -0.16217900 | -0.00942200 |

| | | | |
|---|---|---|---|
| C | 3.26155000 | -0.22267100 | 0.00971000 |
| C | 4.01670000 | 0.96891900 | 0.09899200 |
| C | 3.36345800 | 2.17401300 | 0.16588800 |
| H | 1.47192600 | 3.21991300 | 0.19783300 |
| C | 3.86863400 | -1.49802800 | -0.06107200 |
| H | 5.10411600 | 0.90643600 | 0.11519600 |
| H | 3.93326400 | 3.09947000 | 0.23819100 |
| C | 3.09324800 | -2.62534300 | -0.14441100 |
| C | 1.69447900 | -2.47830100 | -0.15515000 |
| H | 4.95666400 | -1.56250300 | -0.04766300 |
| H | 3.53139800 | -3.61862900 | -0.20086100 |
| H | 1.04802700 | -3.35375400 | -0.21901400 |
| N | 1.09041200 | -1.30624400 | -0.09055600 |
| N | -0.21765000 | 1.07530400 | 0.01194100 |
| C | -1.05664400 | 2.13787900 | -0.11464900 |
| C | -2.49366200 | 1.69914000 | -0.31214300 |
| H | -3.18026500 | 2.45860700 | 0.09071400 |
| H | -2.66238800 | 1.66812900 | -1.40140500 |
| C | -2.75361100 | 0.31936100 | 0.25333300 |
| H | -2.93358800 | 0.33012000 | 1.34588200 |
| O | -0.73134200 | 3.33113300 | -0.13045500 |
| Pd | -1.06589200 | -0.75021800 | 0.04214700 |
| O | -3.80936500 | -0.29781400 | -0.41252300 |
| C | -4.37302000 | -1.38575200 | 0.30229100 |
| H | -4.80110300 | -1.04279900 | 1.25686400 |
| H | -5.16881400 | -1.80658500 | -0.31930100 |
| H | -3.62459800 | -2.16666800 | 0.50252800 |

**17_OMe**

E (M06-SMD/BS1) = -1435.731193 au

H (M06-SMD/BS1) = -1435.335654 au

G (M06-SMD/BS1) = -1435.425373 au

E (M06-SMD/BS2//M06-SMD/BS1) = -1722.663362 au

| | | | |
|---|---|---|---|
| C | 5.22038500 | 0.42757700 | 0.80234800 |
| C | 3.90512000 | 0.26653500 | 0.38567100 |
| C | 3.48186900 | -1.05100600 | -0.00061600 |
| C | 4.39052900 | -2.14137700 | 0.05066600 |
| C | 5.71896100 | -1.92810000 | 0.48350300 |
| C | 6.10963900 | -0.66376600 | 0.84880300 |
| H | 5.56266800 | 1.41261600 | 1.09831600 |
| C | 3.91481700 | -3.41390300 | -0.34249500 |

| | | | |
|---|---|---|---|
| H | 6.40728300 | -2.77172000 | 0.51756000 |
| H | 7.13082000 | -0.48571900 | 1.18350800 |
| C | 2.61642000 | -3.56648800 | -0.75656100 |
| C | 1.78154500 | -2.43468800 | -0.77716800 |
| H | 4.59799200 | -4.26272500 | -0.31026800 |
| H | 2.22420700 | -4.53207500 | -1.06577600 |
| H | 0.74246400 | -2.52047200 | -1.10268100 |
| N | 2.18840400 | -1.23227600 | -0.41630400 |
| N | 2.93436400 | 1.26350400 | 0.31733600 |
| C | 3.08530200 | 2.56357700 | 0.68454600 |
| C | 1.77296200 | 3.31844300 | 0.64272400 |
| H | 1.96216400 | 4.38852700 | 0.46492300 |
| H | 1.36481400 | 3.24090300 | 1.66333300 |
| C | 0.79309600 | 2.72470200 | -0.36197700 |
| H | 1.04233800 | 3.04851100 | -1.38504400 |
| O | 4.13121800 | 3.10216700 | 1.07068100 |
| Pd | 1.14794800 | 0.72740600 | -0.41861300 |
| C | -5.84063700 | 0.23560700 | 0.45519100 |
| C | -4.62371500 | -0.44261900 | 0.52154200 |
| C | -3.55031300 | 0.07023800 | -0.18686000 |
| C | -3.61052100 | 1.21936300 | -0.94985100 |
| C | -4.83864400 | 1.88095000 | -1.00015500 |
| C | -5.94449300 | 1.39267200 | -0.30602100 |
| H | -6.69144200 | -0.15918800 | 1.00811000 |
| H | -2.74014300 | 1.60143400 | -1.47911700 |
| H | -4.92407100 | 2.79087500 | -1.59133500 |
| H | -6.89293200 | 1.92317600 | -0.35848700 |
| I | -1.77605200 | -1.09677600 | 0.04851100 |
| C | -4.44650800 | -1.67940000 | 1.33014200 |
| O | -5.36309200 | -2.18170300 | 1.97087500 |
| O | -3.23940500 | -2.18030500 | 1.30068900 |
| O | -0.79625600 | 0.24694800 | -1.23325300 |
| C | -0.76878600 | -0.14673800 | -2.60355000 |
| H | -0.37590800 | 0.70660800 | -3.17194600 |
| H | -1.78506700 | -0.37683100 | -2.95664400 |
| H | -0.11769400 | -1.01876600 | -2.76638200 |
| O | -0.54648300 | 3.10468400 | -0.18008200 |
| C | -1.11375800 | 2.79813700 | 1.07675500 |
| H | -2.20110800 | 2.89225600 | 0.97379500 |
| H | -0.78249600 | 3.49280400 | 1.86307800 |
| H | -0.86560000 | 1.76702100 | 1.38185100 |

**$TS_{17\text{-}18\_OMe}$**

E (M06-SMD/BS1) = -1435.714267 au

H (M06-SMD/BS1) = -1435.320514 au

G (M06-SMD/BS1) = -1435.410488 au

E (M06-SMD/BS2//M06-SMD/BS1) = -1722.636815 au

| | | | |
|---|---|---|---|
| C | 4.66348700 | 0.64270400 | 1.35816200 |
| C | 3.50528800 | 0.39532500 | 0.63275600 |
| C | 3.45650100 | -0.79039100 | -0.17249600 |
| C | 4.56310900 | -1.68136600 | -0.20046200 |
| C | 5.72117400 | -1.38840900 | 0.55448900 |
| C | 5.75651700 | -0.24440300 | 1.31149300 |
| H | 4.72234600 | 1.53557300 | 1.96996000 |
| C | 4.45298600 | -2.84453400 | -0.99602600 |
| H | 6.56368200 | -2.07809200 | 0.52041000 |
| H | 6.64388900 | -0.00475900 | 1.89579000 |
| C | 3.30506700 | -3.08432600 | -1.70582600 |
| C | 2.25990400 | -2.14644000 | -1.62896500 |
| H | 5.29183200 | -3.53979300 | -1.02919100 |
| H | 3.18792700 | -3.97149100 | -2.32303500 |
| H | 1.33770600 | -2.30900700 | -2.18657100 |
| N | 2.32658300 | -1.04601700 | -0.90299900 |
| N | 2.37022600 | 1.20405700 | 0.60486100 |
| C | 2.11949000 | 2.24775200 | 1.43667200 |
| C | 0.73564500 | 2.82178000 | 1.22225000 |
| H | 0.73006600 | 3.89270700 | 1.47722100 |
| H | 0.05336700 | 2.32618800 | 1.93348000 |
| C | 0.23944800 | 2.59981200 | -0.18470500 |
| H | 0.83662500 | 3.14094600 | -0.94295300 |
| O | 2.87351900 | 2.69769700 | 2.30789800 |
| Pd | 0.86876600 | 0.69098000 | -0.66117300 |
| C | -5.57586300 | -0.73528400 | 0.40139000 |
| C | -4.22672400 | -0.90675000 | 0.71311800 |
| C | -3.28336100 | -0.30282500 | -0.10883700 |
| C | -3.62130300 | 0.46197400 | -1.20867800 |
| C | -4.97798600 | 0.62092500 | -1.49446700 |
| C | -5.95141800 | 0.02682000 | -0.69597000 |
| H | -6.31637900 | -1.21361700 | 1.04088500 |
| H | -2.85469700 | 0.91429700 | -1.83024500 |
| H | -5.26728700 | 1.21907100 | -2.35698700 |
| H | -7.00545100 | 0.15876300 | -0.93174300 |

| | | | |
|---|---|---|---|
| I | -1.24804900 | -0.64206000 | 0.55482600 |
| C | -3.81880500 | -1.72344800 | 1.89120000 |
| O | -4.64786100 | -2.27186700 | 2.61605900 |
| O | -2.53865300 | -1.81205300 | 2.08978100 |
| O | -0.81832700 | 0.27729500 | -1.78006800 |
| C | -0.76422800 | -0.75747200 | -2.72310900 |
| H | -1.63626400 | -0.66525600 | -3.39334300 |
| H | -0.81583300 | -1.76176600 | -2.26561200 |
| H | 0.14990100 | -0.70941800 | -3.33742000 |
| O | -1.09732600 | 2.89641600 | -0.29111100 |
| C | -1.49922100 | 3.36255500 | -1.56952100 |
| H | -0.98272000 | 4.30239100 | -1.81424200 |
| H | -2.57705300 | 3.54505700 | -1.52281300 |
| H | -1.28503100 | 2.61212400 | -2.34372100 |

**19'_OMe**

E (M06-SMD/BS1) = -1435.751932 au
H (M06-SMD/BS1) = -1435.35699 au
G (M06-SMD/BS1) = -1435.448883 au
E (M06-SMD/BS2//M06-SMD/BS1) = -1722.665733 au

| | | | |
|---|---|---|---|
| C | 4.23294800 | -1.72592800 | -0.72848100 |
| C | 3.20739700 | -0.91680300 | -0.27136900 |
| C | 3.52807900 | 0.41995200 | 0.10111900 |
| C | 4.86093000 | 0.89507400 | 0.07436600 |
| C | 5.88208400 | 0.02996400 | -0.38359400 |
| C | 5.55835800 | -1.24264100 | -0.78398000 |
| H | 4.01846000 | -2.74168600 | -1.04038700 |
| C | 5.08891500 | 2.22366100 | 0.49909500 |
| H | 6.90736100 | 0.39489400 | -0.42101000 |
| H | 6.33661800 | -1.90893800 | -1.15248500 |
| C | 4.03808600 | 3.00784000 | 0.90792200 |
| C | 2.73701300 | 2.47926900 | 0.87302100 |
| H | 6.10682200 | 2.61220600 | 0.49178500 |
| H | 4.18586200 | 4.03247300 | 1.23838700 |
| H | 1.87020400 | 3.08205400 | 1.13900800 |
| N | 2.50071900 | 1.23731100 | 0.49012500 |
| N | 1.86093200 | -1.26626100 | -0.07153300 |
| C | 1.52061600 | -2.52030600 | 0.30597700 |
| C | 0.16796300 | -2.61841700 | 0.99448300 |
| H | 0.20196800 | -3.49651800 | 1.66470000 |
| H | -0.67070400 | -2.79313200 | 0.30621000 |

| | | | |
|---|---|---|---|
| C | -0.11729800 | -1.48354800 | 1.87908200 |
| H | 0.67824100 | -0.98082800 | 2.44710500 |
| O | 2.21683100 | -3.53230900 | 0.19459100 |
| Pd | 0.67446300 | 0.43618000 | -0.06846100 |
| C | -4.87165900 | 0.85680500 | 0.59534100 |
| C | -3.86894600 | 0.00581700 | 0.11188100 |
| C | -3.03014500 | 0.53375000 | -0.87193400 |
| C | -3.18605000 | 1.82027400 | -1.38151000 |
| C | -4.21063800 | 2.62629400 | -0.89518300 |
| C | -5.05055000 | 2.14288000 | 0.10269100 |
| H | -5.52456000 | 0.47075000 | 1.37646300 |
| H | -2.50563600 | 2.19890300 | -2.14167800 |
| H | -4.34062900 | 3.63068000 | -1.29452200 |
| H | -5.84962600 | 2.76822300 | 0.49760200 |
| I | -1.32133200 | -0.52805300 | -1.62840600 |
| C | -3.81843600 | -1.41095300 | 0.68246400 |
| O | -4.21949500 | -1.54002300 | 1.86307700 |
| O | -3.41335300 | -2.32179600 | -0.07969400 |
| O | -0.17721600 | 2.24703900 | -0.08942800 |
| C | -0.87061400 | 2.48935800 | 1.09230000 |
| H | -1.73724900 | 1.81244000 | 1.23450400 |
| H | -1.27569000 | 3.51759700 | 1.06907500 |
| H | -0.22938000 | 2.41748400 | 1.99571000 |
| O | -1.31992500 | -1.20516500 | 2.14021400 |
| C | -1.65589700 | -0.26121100 | 3.19376600 |
| H | -1.97716500 | -0.86306500 | 4.04726500 |
| H | -2.48717400 | 0.33391200 | 2.81326900 |
| H | -0.78166200 | 0.34898800 | 3.43875800 |

**22_OMe**

E (M06-SMD/BS1) = -1551.470885 au
H (M06-SMD/BS1) = -1551.015221 au
G (M06-SMD/BS1) = -1551.111948 au
E (M06-SMD/BS2//M06-SMD/BS1) = -1838.427742 au

| | | | |
|---|---|---|---|
| C | 4.04386400 | -0.02665200 | 1.78016500 |
| C | 2.98074700 | -0.21452900 | 0.91244700 |
| C | 3.20242300 | -1.02789000 | -0.23678000 |
| C | 4.47915100 | -1.56368800 | -0.53247400 |
| C | 5.54215900 | -1.32643500 | 0.36956200 |
| C | 5.30998700 | -0.58611200 | 1.50223800 |
| H | 3.90627900 | 0.56299400 | 2.67908300 |

| | | | |
|---|---|---|---|
| C | 4.61191200 | -2.32126900 | -1.71811700 |
| H | 6.52324800 | -1.74748300 | 0.15448700 |
| H | 6.11819600 | -0.41433700 | 2.21157900 |
| C | 3.52424900 | -2.52854900 | -2.53040700 |
| C | 2.27918500 | -1.99831300 | -2.15267500 |
| H | 5.58625600 | -2.73889400 | -1.97049000 |
| H | 3.59864000 | -3.10767300 | -3.44697700 |
| H | 1.38074900 | -2.18193200 | -2.73993500 |
| N | 2.13310300 | -1.27621200 | -1.05649100 |
| N | 1.69483000 | 0.34753900 | 0.99362000 |
| C | 1.55422900 | 1.63601900 | 1.40484600 |
| C | 0.35155800 | 2.38958500 | 0.86828500 |
| H | 0.28237100 | 3.34979300 | 1.39509800 |
| H | -0.58564500 | 1.84555200 | 1.03261200 |
| C | 0.50519300 | 2.63281500 | -0.62958100 |
| O | 2.38465500 | 2.24105700 | 2.09791200 |
| Pd | 0.36473100 | -0.77677600 | -0.11570700 |
| C | -4.78837400 | 0.06349100 | -1.15476100 |
| C | -3.76579500 | 0.20112000 | -0.21258900 |
| C | -3.33014300 | -0.93271900 | 0.47517400 |
| C | -3.89776000 | -2.17908100 | 0.24273000 |
| C | -4.93759200 | -2.29162200 | -0.67736200 |
| C | -5.38093200 | -1.17314700 | -1.37586500 |
| H | -5.11779500 | 0.94191600 | -1.70796900 |
| H | -3.52501500 | -3.05949700 | 0.76258100 |
| H | -5.38966900 | -3.26566200 | -0.85507100 |
| H | -6.18429300 | -1.26499700 | -2.10393800 |
| I | -1.62832900 | -0.85514600 | 1.77175700 |
| C | -3.14043300 | 1.53385000 | -0.00036200 |
| O | -2.69949400 | 2.06375000 | -1.13526400 |
| O | -3.02157300 | 2.07214200 | 1.08336100 |
| O | -0.73349900 | -1.94540700 | -1.32783000 |
| C | -1.20755000 | -1.16952500 | -2.38330500 |
| H | -1.83526500 | -0.31810200 | -2.05261600 |
| H | -1.84095300 | -1.79131800 | -3.04272700 |
| H | -0.39627700 | -0.75197300 | -3.01735900 |
| H | 0.33181100 | 1.69627400 | -1.19601400 |
| C | -0.47760100 | 3.81748700 | -2.41668200 |
| H | -0.56563600 | 2.86445600 | -2.96304600 |
| H | 0.42707200 | 4.34042700 | -2.75057000 |
| H | -1.35344000 | 4.43932900 | -2.62663200 |

| | | | |
|---|---|---|---|
| O | -0.45834300 | 3.59049200 | -1.01081600 |
| H | -2.14247000 | 2.85487300 | -0.91610700 |
| O | 1.79572000 | 3.02669100 | -1.01074000 |
| C | 2.24571300 | 4.25969200 | -0.46131300 |
| H | 2.44410400 | 4.17446600 | 0.61496000 |
| H | 1.52154400 | 5.06680000 | -0.63639600 |
| H | 3.17962200 | 4.50314800 | -0.97707600 |

**31**

E (M06-SMD/BS1) = -652.4065225 au

H (M06-SMD/BS1) = -652.105395 au

G (M06-SMD/BS1) = -652.164535 au

E (M06-SMD/BS2//M06-SMD/BS1) = -652.6584964 au

| | | | |
|---|---|---|---|
| C | 2.81888900 | -1.83750200 | -0.38782800 |
| C | 3.20255700 | -0.38581800 | -0.65346400 |
| H | 3.42034200 | -0.24180200 | -1.72340000 |
| C | 2.20373000 | 0.68821600 | -0.26775500 |
| C | -0.24922100 | 1.13484700 | 0.06616400 |
| C | -0.39882000 | 2.33253300 | -0.87117700 |
| H | 0.50590100 | 2.94999100 | -0.83372200 |
| H | -1.24945200 | 2.96186100 | -0.58186200 |
| H | -0.55004000 | 1.99751100 | -1.90596100 |
| C | -0.11291600 | 1.58794900 | 1.52260300 |
| H | -0.00360100 | 0.71681200 | 2.18395200 |
| H | -1.00172800 | 2.15026800 | 1.83653000 |
| H | 0.76369600 | 2.23341900 | 1.64549100 |
| C | -1.47241400 | 0.22129400 | -0.03196100 |
| C | -2.75808400 | 0.73656500 | 0.15166500 |
| H | -2.91039800 | 1.79669100 | 0.34554900 |
| C | -3.84217000 | -0.12665100 | 0.08866200 |
| H | -4.85297400 | 0.25348200 | 0.22724900 |
| C | -3.62043900 | -1.47947300 | -0.15331600 |
| H | -4.44214600 | -2.18959200 | -0.21083700 |
| C | -2.31108400 | -1.90255800 | -0.32004100 |
| H | -2.08811500 | -2.95347500 | -0.51076800 |
| N | 0.90830700 | 0.33084000 | -0.32971500 |
| O | 2.59477000 | 1.82279600 | 0.03659000 |
| N | -1.26006900 | -1.07782600 | -0.26002600 |
| H | 4.13073000 | -0.13718600 | -0.11925100 |
| H | 3.63692300 | -2.47172300 | -0.75856900 |
| C | 2.57304500 | -2.13756500 | 1.08239000 |

| | | | |
|---|---|---|---|
| H | 2.37638800 | -3.20517500 | 1.24457200 |
| H | 3.44713500 | -1.86275500 | 1.69006100 |
| H | 1.70936200 | -1.58092000 | 1.47480300 |
| H | 0.67401200 | -0.63764000 | -0.53638200 |
| H | 1.93971200 | -2.12425800 | -0.98763300 |

**32 (PIDA)**

E (M06-SMD/BS1) = -699.5035232 au
H (M06-SMD/BS1) = -699.293435 au
G (M06-SMD/BS1) = -699.360821 au
E (M06-SMD/BS2//M06-SMD/BS1) = -986.1874142 au

| | | | |
|---|---|---|---|
| C | 0.16764200 | -1.51068800 | -0.00044200 |
| C | 0.79221000 | -2.10586000 | 1.08777800 |
| C | 0.94782400 | -3.48958700 | 1.07914200 |
| C | 0.47548900 | -4.24096000 | 0.00625800 |
| C | -0.15152600 | -3.61879000 | -1.07006300 |
| C | -0.30795700 | -2.23514000 | -1.08550300 |
| H | 1.15342300 | -1.50802600 | 1.92239100 |
| H | 1.43624800 | -3.97867600 | 1.92001100 |
| H | 0.59787500 | -5.32263700 | 0.00896300 |
| H | -0.51787900 | -4.20844400 | -1.90849600 |
| H | -0.79240200 | -1.73612400 | -1.92253900 |
| I | -0.06864200 | 0.60618800 | -0.00164800 |
| O | -2.21201000 | 0.06204700 | 0.08067000 |
| C | -2.91038100 | 1.16248100 | 0.10186700 |
| O | -2.38989400 | 2.27973800 | 0.08058900 |
| O | 2.13939700 | 0.54137700 | -0.09949700 |
| C | 2.58462500 | 1.76694400 | -0.10043900 |
| O | 1.83596900 | 2.74492100 | -0.05270900 |
| C | 4.07874300 | 1.87603500 | -0.16787800 |
| H | 4.44258200 | 1.39860000 | -1.08560300 |
| H | 4.38604900 | 2.92529800 | -0.15190000 |
| H | 4.52914300 | 1.34458500 | 0.67874000 |
| C | -4.39340200 | 0.94559400 | 0.15595800 |
| H | -4.65196700 | 0.39129100 | 1.06621100 |
| H | -4.92065400 | 1.90349400 | 0.14633600 |
| H | -4.71169800 | 0.33786200 | -0.69928300 |

**34**

E (M06-SMD/BS1) = -1237.119136 au
H (M06-SMD/BS1) = -1236.700449 au

G (M06-SMD/BS1) = -1236.78654 au

E (M06-SMD/BS2//M06-SMD/BS1) = -1237.580445 au

| | | | |
|---|---|---|---|
| C | 3.33143100 | 0.26690700 | -1.98726600 |
| C | 3.26053100 | -0.57865800 | -0.72266200 |
| H | 4.26693400 | -0.74594000 | -0.31340100 |
| C | 2.43239200 | -0.08861000 | 0.43563800 |
| C | 0.74119300 | 1.73722100 | 1.08792000 |
| C | 1.78691800 | 2.81790900 | 1.31184300 |
| H | 2.71195100 | 2.36768800 | 1.69265000 |
| H | 1.44244800 | 3.53811100 | 2.06372400 |
| H | 2.01106700 | 3.36078700 | 0.38368200 |
| C | 0.31496400 | 1.10711500 | 2.41387600 |
| H | -0.32910300 | 0.23058400 | 2.26042700 |
| H | -0.25488900 | 1.85881600 | 2.97642600 |
| H | 1.18136100 | 0.81137900 | 3.01102200 |
| C | -0.51261800 | 2.26338500 | 0.40850900 |
| C | -0.97561800 | 3.56385800 | 0.54735200 |
| H | -0.40971000 | 4.28656900 | 1.12957900 |
| C | -2.16962800 | 3.92878800 | -0.06461400 |
| H | -2.54260300 | 4.94566400 | 0.03676700 |
| C | -2.87535400 | 2.99043900 | -0.80735300 |
| H | -3.80903900 | 3.24002500 | -1.30385900 |
| C | -2.36401300 | 1.70813200 | -0.91159200 |
| H | -2.86395500 | 0.92756500 | -1.48195100 |
| N | 1.29191500 | 0.72045800 | 0.10649500 |
| O | 2.65262500 | -0.42315200 | 1.57945800 |
| N | -1.21673700 | 1.36248200 | -0.31112100 |
| H | 2.85202100 | -1.57601100 | -0.95291700 |
| H | 2.32354300 | 0.43706200 | -2.40062300 |
| C | 4.07079500 | 1.58018800 | -1.78762400 |
| H | 4.12453900 | 2.15384900 | -2.72136000 |
| H | 3.58938200 | 2.22210500 | -1.03437900 |
| H | 5.09906100 | 1.39612700 | -1.44648400 |
| H | 1.46341600 | 1.20275900 | -0.78293800 |
| H | 3.85105000 | -0.33021100 | -2.74902800 |
| Pd | -0.37045100 | -0.49018600 | -0.42939400 |
| O | -2.06963400 | -1.45579200 | -0.99731400 |
| C | -2.98237100 | -1.58261200 | -0.07998500 |
| O | -2.88493900 | -1.15895400 | 1.07510500 |
| O | 0.61114300 | -2.27691900 | -0.55807200 |
| C | 0.67939600 | -2.94691700 | 0.55157100 |

| | | | |
|---|---|---|---|
| O | 0.11402000 | -2.62248700 | 1.60148800 |
| C | -4.21790400 | -2.29460800 | -0.56510500 |
| H | -3.95258700 | -3.27357100 | -0.98195400 |
| H | -4.93515100 | -2.42463700 | 0.25066700 |
| H | -4.68503100 | -1.71311900 | -1.37028900 |
| C | 1.57148100 | -4.15703000 | 0.48340800 |
| H | 1.27121200 | -4.89818000 | 1.23112100 |
| H | 1.57521500 | -4.60702500 | -0.51481400 |
| H | 2.59808100 | -3.83702800 | 0.71362900 |

**$TS_{34\text{-}35}$**

E (M06-SMD/BS1) = -1237.113665 au

H (M06-SMD/BS1) = -1236.701033 au

G (M06-SMD/BS1) = -1236.78396 au

E (M06-SMD/BS2//M06-SMD/BS1) = -1237.575824 au

| | | | |
|---|---|---|---|
| C | 1.09034900 | 1.74597600 | -2.66317800 |
| C | 0.83119000 | 2.64688900 | -1.44819900 |
| H | 0.58428900 | 3.65646700 | -1.80805000 |
| C | -0.34943700 | 2.22188600 | -0.60297100 |
| C | -1.22883200 | 1.20590500 | 1.45286100 |
| C | -1.91849100 | 2.48459600 | 1.91878100 |
| H | -1.15348200 | 3.18530900 | 2.27905800 |
| H | -2.58907000 | 2.26769900 | 2.76036100 |
| H | -2.49288500 | 2.97161100 | 1.12667200 |
| C | -0.68116500 | 0.50144200 | 2.70051100 |
| H | -0.12555900 | -0.41442100 | 2.45562700 |
| H | -1.51051000 | 0.23101300 | 3.36755700 |
| H | -0.01054200 | 1.18144100 | 3.24374600 |
| C | -2.14749500 | 0.24356800 | 0.72875900 |
| C | -3.52378700 | 0.17344800 | 0.89250000 |
| H | -4.02529900 | 0.87306600 | 1.55740800 |
| C | -4.24377400 | -0.79068800 | 0.19702000 |
| H | -5.32356200 | -0.85359300 | 0.31579700 |
| C | -3.57880200 | -1.66945200 | -0.65175900 |
| H | -4.10989900 | -2.43320400 | -1.21319300 |
| C | -2.20424300 | -1.55981900 | -0.77325200 |
| H | -1.61927500 | -2.21704700 | -1.41420000 |
| N | -0.07516900 | 1.48736700 | 0.54973300 |
| O | -1.48888700 | 2.54002400 | -0.94302700 |
| N | -1.52043000 | -0.62965100 | -0.09233000 |
| H | 1.74422400 | 2.72575300 | -0.84443300 |

| | | | |
|---|---|---|---|
| H | 1.49020900 | 0.77654200 | -2.32443900 |
| C | -0.12475000 | 1.51039300 | -3.54382100 |
| H | 0.15775300 | 0.96876800 | -4.45602800 |
| H | -0.89019300 | 0.91087500 | -3.03022100 |
| H | -0.59352800 | 2.45751100 | -3.84693600 |
| H | 1.27545300 | 1.62048500 | 1.14456400 |
| H | 1.88998100 | 2.21809300 | -3.25227100 |
| Pd | 0.49077200 | -0.38630000 | -0.14531800 |
| O | 0.87953900 | -2.26474400 | -0.89520000 |
| C | 0.88060000 | -3.20749100 | -0.00738100 |
| O | 0.70439200 | -3.02790400 | 1.20516200 |
| O | 2.55531800 | -0.12622300 | 0.08805300 |
| C | 3.01965100 | 0.71997400 | 0.88863700 |
| O | 2.30996200 | 1.62105500 | 1.48073800 |
| C | 1.09022200 | -4.58575100 | -0.57609000 |
| H | 1.94439200 | -4.59468600 | -1.26307600 |
| H | 1.24774900 | -5.31726700 | 0.22211900 |
| H | 0.20249800 | -4.87401200 | -1.15476000 |
| C | 4.47423200 | 0.73141600 | 1.19910500 |
| H | 4.61407600 | 0.65002400 | 2.28367400 |
| H | 4.99013600 | -0.08791100 | 0.69325300 |
| H | 4.90131100 | 1.69184600 | 0.88561100 |

**35**

E (M06-SMD/BS1) = -1237.113876 au

H (M06-SMD/BS1) = -1236.698038 au

G (M06-SMD/BS1) = -1236.781972 au

E (M06-SMD/BS2//M06-SMD/BS1) = -1237.576255 au

| | | | |
|---|---|---|---|
| C | 0.96679100 | -1.75125000 | 2.68148500 |
| C | 0.73422400 | -2.64623400 | 1.45704300 |
| H | 0.48454600 | -3.65859500 | 1.80744300 |
| C | -0.43032300 | -2.22302800 | 0.58612600 |
| C | -1.26759600 | -1.17185800 | -1.46258900 |
| C | -1.99829400 | -2.42235200 | -1.94326000 |
| H | -1.25459800 | -3.15087800 | -2.29308300 |
| H | -2.64593600 | -2.17783500 | -2.79535500 |
| H | -2.60310900 | -2.89011800 | -1.16265800 |
| C | -0.68697200 | -0.48192500 | -2.70404800 |
| H | -0.10459200 | 0.41505700 | -2.45164400 |
| H | -1.49926700 | -0.18387500 | -3.38047600 |
| H | -0.03152600 | -1.18240200 | -3.24021400 |

| | | | |
|---|---|---|---|
| C | -2.15732900 | -0.18063200 | -0.74318800 |
| C | -3.53033800 | -0.06185700 | -0.90668700 |
| H | -4.05542200 | -0.74106400 | -1.57457600 |
| C | -4.21628000 | 0.92371000 | -0.20666900 |
| H | -5.29337900 | 1.02448900 | -0.32421100 |
| C | -3.52084600 | 1.77550600 | 0.64528300 |
| H | -4.02502900 | 2.55471400 | 1.21027600 |
| C | -2.15083600 | 1.61808500 | 0.76566600 |
| H | -1.54315700 | 2.25212500 | 1.40886300 |
| N | -0.13106900 | -1.49116700 | -0.55346100 |
| O | -1.57484400 | -2.55532400 | 0.90511200 |
| N | -1.50028600 | 0.66702100 | 0.08084400 |
| H | 1.65937600 | -2.71884900 | 0.87088900 |
| H | 1.39052200 | -0.78628400 | 2.35814600 |
| C | -0.27288100 | -1.49998100 | 3.52284600 |
| H | -0.01308000 | -0.96515200 | 4.44577900 |
| H | -1.01164700 | -0.88783200 | 2.98542900 |
| H | -0.76582000 | -2.44077500 | 3.80668300 |
| H | 1.37564700 | -1.65328000 | -1.19864100 |
| H | 1.74105300 | -2.23376900 | 3.29575800 |
| Pd | 0.50123000 | 0.35743500 | 0.13906000 |
| O | 0.94146700 | 2.22746000 | 0.89415800 |
| C | 0.96445700 | 3.17332200 | 0.01069100 |
| O | 0.78262100 | 3.00356500 | -1.20279800 |
| O | 2.57238200 | 0.05087500 | -0.05034000 |
| C | 3.06494600 | -0.77589500 | -0.84411500 |
| O | 2.36887600 | -1.66638900 | -1.48612800 |
| C | 1.20827800 | 4.54423100 | 0.58398000 |
| H | 2.06286000 | 4.52986500 | 1.27039800 |
| H | 1.38309900 | 5.27473500 | -0.21157200 |
| H | 0.32836000 | 4.85218500 | 1.16449300 |
| C | 4.52563000 | -0.80385000 | -1.10956000 |
| H | 4.70059800 | -0.71810000 | -2.18864100 |
| H | 5.03274700 | 0.00646300 | -0.58129800 |
| H | 4.93081600 | -1.77124100 | -0.78829400 |

**36**

E (M06-SMD/BS1) = -1008.110912 au

H (M06-SMD/BS1) = -1007.763247 au

G (M06-SMD/BS1) = -1007.837463 au

E (M06-SMD/BS2//M06-SMD/BS1) = -1008.482903 au

| | | | |
|---|---|---|---|
| C | 0.31830100 | -3.00671000 | 0.18484000 |
| C | -0.99076000 | -3.04497400 | -0.58810000 |
| H | -1.54106400 | -3.95047800 | -0.29857600 |
| C | -1.94827600 | -1.87678400 | -0.43089700 |
| C | -2.21338900 | 0.56141900 | -0.13124300 |
| C | -3.05646500 | 0.47425600 | 1.14665300 |
| H | -3.76188700 | -0.35995900 | 1.07584900 |
| H | -3.62644900 | 1.39926500 | 1.30479500 |
| H | -2.40584900 | 0.31446200 | 2.01892600 |
| C | -3.08655300 | 0.79004700 | -1.36797100 |
| H | -2.46007400 | 0.90440100 | -2.26293100 |
| H | -3.69985200 | 1.69289900 | -1.25629800 |
| H | -3.75722600 | -0.06116100 | -1.51359600 |
| C | -1.26920500 | 1.73193600 | 0.02745100 |
| C | -1.70997200 | 3.05233000 | 0.06398700 |
| H | -2.77026700 | 3.26819600 | -0.04572200 |
| C | -0.79804500 | 4.08031700 | 0.24399000 |
| H | -1.14039000 | 5.11259400 | 0.27042700 |
| C | 0.55341500 | 3.78191900 | 0.39258900 |
| H | 1.29902200 | 4.55860600 | 0.53818700 |
| C | 0.94278000 | 2.45797400 | 0.35575400 |
| H | 1.97881700 | 2.15182600 | 0.48080100 |
| N | -1.38815100 | -0.65805900 | -0.29435700 |
| O | -3.17181400 | -2.08824300 | -0.50097200 |
| N | 0.04726100 | 1.47037000 | 0.17725000 |
| H | -0.78170300 | -3.14047600 | -1.66482400 |
| H | 1.05383200 | -2.22804700 | -0.21080300 |
| C | 0.16901100 | -3.03288400 | 1.69653800 |
| H | 1.13676800 | -2.89864400 | 2.19641300 |
| H | -0.51360200 | -2.25601500 | 2.06774300 |
| H | -0.23719100 | -4.00668500 | 2.00354900 |
| H | 0.92019700 | -3.87442800 | -0.12676800 |
| Pd | 0.55439900 | -0.46921000 | 0.06856300 |
| O | 2.55352900 | -0.19488800 | 0.54619400 |
| C | 3.33456500 | -0.03188500 | -0.47436100 |
| O | 2.95318000 | -0.04620500 | -1.65249000 |
| C | 4.77409000 | 0.22672600 | -0.11852500 |
| H | 5.10790700 | -0.44533000 | 0.68011000 |
| H | 5.41879600 | 0.11452300 | -0.99557800 |
| H | 4.86793700 | 1.25450700 | 0.25807000 |

**$TS_{36\text{-}37}$**

E (M06-SMD/BS1) = -1008.084031 au

H (M06-SMD/BS1) = -1007.742553 au

G (M06-SMD/BS1) = -1007.811391 au

E (M06-SMD/BS2//M06-SMD/BS1) = -1008.457194 au

| | | | |
|---|---|---|---|
| C | 1.78133200 | -1.89168800 | 0.15666600 |
| C | 0.81635000 | -2.87011900 | -0.50472500 |
| H | 0.99461100 | -3.88962200 | -0.12698900 |
| C | -0.64906800 | -2.54832400 | -0.32897900 |
| C | -2.25107400 | -0.68077400 | -0.06211000 |
| C | -2.82693400 | -1.15134200 | 1.27889200 |
| H | -2.91776200 | -2.24287300 | 1.28578200 |
| H | -3.82042900 | -0.71859900 | 1.45636300 |
| H | -2.16434000 | -0.84775200 | 2.10272800 |
| C | -3.15857600 | -1.07818700 | -1.22998500 |
| H | -2.75791000 | -0.68854500 | -2.17583000 |
| H | -4.17514300 | -0.68695400 | -1.09756700 |
| H | -3.21958900 | -2.16838600 | -1.29740600 |
| C | -2.14202700 | 0.83046200 | -0.01063400 |
| C | -3.26537100 | 1.65364500 | 0.03573100 |
| H | -4.26016500 | 1.21415400 | 0.02018800 |
| C | -3.10862200 | 3.02925000 | 0.10750800 |
| H | -3.98322600 | 3.67579400 | 0.14203300 |
| C | -1.82738000 | 3.57260400 | 0.13489200 |
| H | -1.66165800 | 4.64517600 | 0.18983600 |
| C | -0.74917700 | 2.70971900 | 0.09410500 |
| H | 0.27864800 | 3.06604700 | 0.11775700 |
| N | -0.89285500 | -1.22733400 | -0.25775900 |
| O | -1.50795500 | -3.44922700 | -0.30811600 |
| N | -0.90924400 | 1.37727600 | 0.02512200 |
| H | 1.00610200 | -2.90623100 | -1.58928400 |
| H | 2.39394100 | -0.73716700 | -0.42857100 |
| C | 1.83773200 | -2.00143900 | 1.67488600 |
| H | 2.51563200 | -1.24889800 | 2.10379300 |
| H | 0.85234000 | -1.86657800 | 2.14392600 |
| H | 2.21144300 | -2.99403700 | 1.97592700 |
| H | 2.77832000 | -2.19750000 | -0.20696500 |
| Pd | 0.63678100 | 0.01630900 | -0.02937600 |
| O | 2.12550800 | 1.48939000 | 0.23037200 |
| C | 3.25710600 | 1.15655600 | -0.23049000 |
| O | 3.47526100 | 0.00460000 | -0.71443700 |

| | | | |
|---|---|---|---|
| C | 4.37255000 | 2.14865700 | -0.21751100 |
| H | 4.08690400 | 3.06514400 | 0.30535200 |
| H | 5.25434000 | 1.70474700 | 0.25860500 |
| H | 4.64715200 | 2.39006300 | -1.25196200 |

**37**

E (M06-SMD/BS1) = -1008.11293 au

H (M06-SMD/BS1) = -1007.765456 au

G (M06-SMD/BS1) = -1007.840232 au

E (M06-SMD/BS2//M06-SMD/BS1) = -1008.48581 au

| | | | |
|---|---|---|---|
| C | 1.51838200 | -2.02331400 | 0.21804600 |
| C | 0.55386600 | -2.95707100 | -0.50804200 |
| H | 0.63627500 | -3.99917400 | -0.15414800 |
| C | -0.89368900 | -2.51164600 | -0.38992400 |
| C | -2.29471800 | -0.47617200 | -0.14652400 |
| C | -3.06609200 | -0.94405400 | 1.09340200 |
| H | -3.27526200 | -2.01721200 | 1.02133800 |
| H | -4.02083100 | -0.41188300 | 1.19809700 |
| H | -2.46953500 | -0.76389200 | 1.99974000 |
| C | -3.10987500 | -0.70285800 | -1.42484100 |
| H | -2.56600600 | -0.31571000 | -2.29789600 |
| H | -4.08421000 | -0.20027900 | -1.37607900 |
| H | -3.28645700 | -1.77304100 | -1.57083500 |
| C | -2.03890800 | 1.02372000 | -0.00168400 |
| C | -3.10072100 | 1.93032300 | 0.02797300 |
| H | -4.12555800 | 1.57532500 | -0.05727600 |
| C | -2.84526400 | 3.28596400 | 0.16856500 |
| H | -3.67005900 | 3.99597400 | 0.19145800 |
| C | -1.52943100 | 3.72689000 | 0.27844200 |
| H | -1.28670400 | 4.78082000 | 0.38820600 |
| C | -0.52424200 | 2.77763800 | 0.24827700 |
| H | 0.52688300 | 3.05472200 | 0.33409400 |
| N | -1.00277000 | -1.17722200 | -0.23728200 |
| O | -1.83382600 | -3.32547800 | -0.48242000 |
| N | -0.77223800 | 1.46590200 | 0.11462100 |
| H | 0.77956300 | -2.97962100 | -1.58674700 |
| H | 2.19427300 | 0.30826300 | -1.64946900 |
| C | 1.68990700 | -2.38764100 | 1.67937500 |
| H | 2.32184100 | -1.66685700 | 2.21854400 |
| H | 0.72058600 | -2.43662800 | 2.20033500 |
| H | 2.16231400 | -3.38104000 | 1.78688700 |

| | | | |
|---|---|---|---|
| H | 2.49818200 | -1.98427000 | -0.28505100 |
| Pd | 0.66642400 | -0.16806100 | 0.14556200 |
| O | 2.53052000 | 0.84891400 | 0.56269800 |
| C | 3.31783500 | 1.09613900 | -0.35586900 |
| O | 3.07140100 | 0.76392900 | -1.60817900 |
| C | 4.61387400 | 1.78705500 | -0.15535100 |
| H | 4.75712400 | 2.03139600 | 0.89936400 |
| H | 5.42902500 | 1.14252700 | -0.50591000 |
| H | 4.63780100 | 2.70188300 | -0.75973800 |

**38**

E (M06-SMD/BS1) = -779.1100022 au

H (M06-SMD/BS1) = -778.83203 au

G (M06-SMD/BS1) = -778.892463 au

E (M06-SMD/BS2//M06-SMD/BS1) = -779.3889206 au

| | | | |
|---|---|---|---|
| C | -2.67880200 | -0.93612400 | -0.14062700 |
| C | -2.96711500 | 0.48578300 | -0.60759000 |
| H | -3.90770000 | 0.88162300 | -0.18936700 |
| C | -1.83521400 | 1.43418800 | -0.26033100 |
| C | 0.62817000 | 1.48687000 | 0.08756600 |
| C | 0.58203500 | 2.07876700 | 1.50143800 |
| H | -0.21782700 | 2.82461600 | 1.56865700 |
| H | 1.53131000 | 2.56408100 | 1.76439900 |
| H | 0.38455300 | 1.28491000 | 2.23682700 |
| C | 0.90014900 | 2.57211300 | -0.96036800 |
| H | 0.98961700 | 2.12467500 | -1.95992800 |
| H | 1.82658500 | 3.11845600 | -0.74326100 |
| H | 0.07686500 | 3.29256600 | -0.97234200 |
| C | 1.77220200 | 0.47351800 | 0.04589100 |
| C | 3.09606200 | 0.89661700 | 0.18176400 |
| H | 3.32264600 | 1.95315700 | 0.30713400 |
| C | 4.12341800 | -0.03405100 | 0.16027200 |
| H | 5.15663300 | 0.29203500 | 0.26447400 |
| C | 3.81896500 | -1.38287700 | 0.00376700 |
| H | 4.59330300 | -2.14548500 | -0.02092000 |
| C | 2.48738900 | -1.73630800 | -0.11730600 |
| H | 2.18489600 | -2.77618800 | -0.23721800 |
| N | -0.64104000 | 0.80341100 | -0.21779800 |
| O | -2.02307400 | 2.65289300 | -0.09325000 |
| N | 1.49159600 | -0.83648900 | -0.09433100 |
| H | -3.07566300 | 0.51995500 | -1.70338100 |

| | | | |
|---|---|---|---|
| C | -3.12981800 | -1.18707200 | 1.28198800 |
| H | -2.81889700 | -2.17437000 | 1.65376800 |
| H | -2.72875000 | -0.42647900 | 1.97036600 |
| H | -4.23146300 | -1.14411900 | 1.36100700 |
| H | -3.09477000 | -1.68952300 | -0.82977400 |
| Pd | -0.66402800 | -1.18261000 | -0.18692000 |

**39**

E (M06-SMD/BS1) = -1478.663362 au

H (M06-SMD/BS1) = -1478.172961 au

G (M06-SMD/BS1) = -1478.274095 au

E (M06-SMD/BS2//M06-SMD/BS1) = -1765.613528 au

| | | | |
|---|---|---|---|
| Pd | -0.05572500 | 1.21317400 | 1.04373200 |
| C | 1.60306900 | 2.26614500 | 0.46441700 |
| C | 1.12379300 | 3.28128600 | -0.57329900 |
| H | 1.89706400 | 3.50907000 | -1.32593800 |
| C | -0.16029600 | 2.89035900 | -1.27977700 |
| C | -2.25990000 | 1.61313300 | -0.95290700 |
| C | -2.17924800 | 0.80337500 | -2.25475900 |
| H | -1.73516700 | 1.41288300 | -3.04952300 |
| H | -3.17557400 | 0.47841500 | -2.58412500 |
| H | -1.55987400 | -0.09490800 | -2.11168800 |
| C | -3.19616900 | 2.81574500 | -1.11348400 |
| H | -3.28417400 | 3.35926300 | -0.16234700 |
| H | -4.20146300 | 2.50809800 | -1.42862400 |
| H | -2.79706100 | 3.49955400 | -1.86997900 |
| C | -2.83095000 | 0.68596100 | 0.11898300 |
| C | -4.12338500 | 0.16798300 | 0.00021000 |
| H | -4.74612000 | 0.44121300 | -0.84920700 |
| C | -4.60888800 | -0.70024500 | 0.96635200 |
| H | -5.61496200 | -1.10632200 | 0.87835700 |
| C | -3.79780600 | -1.04591600 | 2.04391300 |
| H | -4.13870800 | -1.72502000 | 2.82137900 |
| C | -2.52728200 | -0.50100000 | 2.09900400 |
| H | -1.83979400 | -0.73766400 | 2.91230200 |
| N | -0.92508400 | 2.07482900 | -0.53132600 |
| O | -0.44651000 | 3.34895900 | -2.40585400 |
| N | -2.05913300 | 0.34045800 | 1.16543000 |
| H | 0.90431600 | 4.24038500 | -0.07010400 |
| H | 2.24361100 | 1.50935900 | -0.02351000 |
| C | 2.35370700 | 2.91796100 | 1.60585600 |

| | | | |
|---|---|---|---|
| H | 2.79342600 | 2.18434400 | 2.29566000 |
| H | 3.18257900 | 3.54311900 | 1.22595100 |
| H | 1.69970400 | 3.57979000 | 2.19514900 |
| C | 2.07411700 | -0.99972900 | -1.25814700 |
| C | 3.40726300 | -1.37867700 | -1.19214700 |
| C | 4.30954600 | -0.73819000 | -2.03938200 |
| C | 3.86796200 | 0.23791100 | -2.92868500 |
| C | 2.52036300 | 0.58469900 | -2.98287800 |
| C | 1.60388300 | -0.03094100 | -2.13450100 |
| H | 3.74328600 | -2.14634400 | -0.49818000 |
| H | 5.36170300 | -1.01446700 | -2.00398100 |
| H | 4.58075600 | 0.73159700 | -3.58669600 |
| H | 2.17214700 | 1.35066200 | -3.67414700 |
| H | 0.55528300 | 0.25971000 | -2.15001600 |
| I | 0.70546400 | -1.88458000 | 0.11867100 |
| O | -0.40198300 | -2.38364700 | -1.66627400 |
| C | -1.60872600 | -2.77628400 | -1.32624500 |
| O | -1.94357500 | -2.92896200 | -0.15352700 |
| O | 2.18392800 | -1.26815100 | 1.72511300 |
| C | 1.88915200 | -0.48341400 | 2.70233700 |
| O | 0.93456300 | 0.32156800 | 2.74436000 |
| C | 2.81683000 | -0.58727100 | 3.87718200 |
| H | 2.55300600 | 0.13934100 | 4.65085900 |
| H | 2.77472100 | -1.60246300 | 4.29015800 |
| H | 3.84727300 | -0.41372000 | 3.54345700 |
| C | -2.52375100 | -3.03773500 | -2.48213700 |
| H | -2.60089100 | -4.12280300 | -2.62872200 |
| H | -3.52376200 | -2.66076600 | -2.23975300 |
| H | -2.15914200 | -2.58167000 | -3.40667400 |

**$TS_{39\text{-}40}$**

E (M06-SMD/BS1) = -1478.655863 au

H (M06-SMD/BS1) = -1478.168355 au

G (M06-SMD/BS1) = -1478.264828 au

E (M06-SMD/BS2//M06-SMD/BS1) = -1765.602163 au

| | | | |
|---|---|---|---|
| Pd | -0.50273900 | -1.58511000 | -0.24210200 |
| C | 0.87375500 | -2.70308900 | 0.77386900 |
| C | 0.89109800 | -2.17899900 | 2.20681600 |
| H | 1.71932200 | -1.46450800 | 2.34533200 |
| C | -0.36330300 | -1.43059800 | 2.62318000 |
| C | -2.31086900 | -0.19051800 | 1.72297900 |

| | | | |
|---|---|---|---|
| C | -1.87573600 | 1.16366100 | 2.30549700 |
| H | -1.45510900 | 1.02482900 | 3.30718500 |
| H | -2.72417600 | 1.85655800 | 2.38137300 |
| H | -1.11023800 | 1.62912400 | 1.66647600 |
| C | -3.37931800 | -0.85132700 | 2.60093300 |
| H | -3.71701300 | -1.79276900 | 2.14532300 |
| H | -4.25306500 | -0.20063500 | 2.73476100 |
| H | -2.96270700 | -1.07219300 | 3.58931600 |
| C | -2.91051600 | 0.07876000 | 0.34191200 |
| C | -4.06168700 | 0.85815400 | 0.20278200 |
| H | -4.55961200 | 1.26252700 | 1.08160900 |
| C | -4.56419400 | 1.12452500 | -1.06219700 |
| H | -5.45966500 | 1.73299100 | -1.17480600 |
| C | -3.91161000 | 0.61381600 | -2.18110700 |
| H | -4.26926600 | 0.80558600 | -3.18958500 |
| C | -2.77746900 | -0.15087500 | -1.97435800 |
| H | -2.21619400 | -0.57920500 | -2.80586600 |
| N | -1.14608400 | -1.08430400 | 1.58364200 |
| O | -0.56303400 | -1.15117400 | 3.82238600 |
| N | -2.29863800 | -0.41498000 | -0.74983700 |
| H | 1.07384600 | -2.98772800 | 2.93568100 |
| H | 1.85278500 | -2.58193600 | 0.28591700 |
| C | 0.39857700 | -4.13805400 | 0.68065900 |
| H | 0.32646200 | -4.49118800 | -0.35761800 |
| H | 1.09709000 | -4.81276100 | 1.20804800 |
| H | -0.58775800 | -4.26942700 | 1.15244700 |
| C | 2.59541800 | 1.04030900 | 0.28245200 |
| C | 3.80701800 | 1.09822300 | -0.39718000 |
| C | 4.97789600 | 1.09732600 | 0.35601500 |
| C | 4.91899600 | 1.04700600 | 1.74708800 |
| C | 3.69186400 | 1.00039900 | 2.40355700 |
| C | 2.50806700 | 0.99539700 | 1.67094000 |
| H | 3.84033200 | 1.14092000 | -1.48342500 |
| H | 5.93976000 | 1.13986400 | -0.15141700 |
| H | 5.84072400 | 1.04648500 | 2.32601900 |
| H | 3.64914300 | 0.96372300 | 3.49048100 |
| H | 1.54149700 | 0.95780100 | 2.17357400 |
| I | 0.76918400 | 1.07035400 | -0.79233500 |
| O | 0.67477800 | 3.18433600 | -0.17068900 |
| C | -0.54299300 | 3.60221400 | -0.40651600 |
| O | -1.39075000 | 2.90213900 | -0.95822100 |

| | | | |
|---|---|---|---|
| O | 1.97571500 | -0.90647400 | -2.03232700 |
| C | 1.31949000 | -1.85012800 | -2.53853000 |
| O | 0.18027800 | -2.25060700 | -2.13532600 |
| C | 1.92154400 | -2.60740400 | -3.69281000 |
| H | 1.14382700 | -2.93387500 | -4.39143400 |
| H | 2.67039200 | -2.00496600 | -4.21543500 |
| H | 2.41371900 | -3.50633300 | -3.29778000 |
| C | -0.81681700 | 4.98647500 | 0.10003100 |
| H | 0.06060500 | 5.63136600 | -0.00562400 |
| H | -1.67365200 | 5.41421100 | -0.42818800 |
| H | -1.05991400 | 4.91542300 | 1.16875300 |

**40**

E (M06-SMD/BS1) = -1478.672403 au
H (M06-SMD/BS1) = -1478.185522 au
G (M06-SMD/BS1) = -1478.285741 au
E (M06-SMD/BS2//M06-SMD/BS1) = -1765.612048 au

| | | | |
|---|---|---|---|
| Pd | 0.96978400 | 0.70921200 | 0.62575000 |
| C | 0.25619100 | 0.41928900 | 2.57292100 |
| C | 0.56317800 | -1.01271300 | 2.94170100 |
| H | -0.32153600 | -1.65466100 | 2.82250900 |
| C | 1.68105900 | -1.64039900 | 2.12774800 |
| C | 3.06501800 | -1.36297200 | 0.07341600 |
| C | 2.52647200 | -2.56687900 | -0.70390700 |
| H | 2.28316200 | -3.38179900 | -0.01250800 |
| H | 3.26818300 | -2.93738400 | -1.42337800 |
| H | 1.61992700 | -2.29173100 | -1.26285100 |
| C | 4.38010200 | -1.70038300 | 0.78862800 |
| H | 4.75137500 | -0.82273500 | 1.33539500 |
| H | 5.14535600 | -2.00504600 | 0.06437500 |
| H | 4.23178500 | -2.52005100 | 1.49552800 |
| C | 3.34984900 | -0.23416500 | -0.90712100 |
| C | 4.33754700 | -0.36534700 | -1.88452800 |
| H | 4.90630400 | -1.28830300 | -1.96695000 |
| C | 4.59474400 | 0.69098400 | -2.74465600 |
| H | 5.36454500 | 0.59528500 | -3.50823800 |
| C | 3.86835300 | 1.87157100 | -2.61745300 |
| H | 4.04851800 | 2.72601100 | -3.26455200 |
| C | 2.89126100 | 1.93017300 | -1.64180400 |
| H | 2.27785800 | 2.81833300 | -1.50050800 |
| N | 2.05942700 | -0.90012400 | 1.05737900 |

| | | | |
|---|---|---|---|
| O | 2.15460900 | -2.73629000 | 2.45417800 |
| N | 2.63531600 | 0.90147600 | -0.81913900 |
| H | 0.85272300 | -1.07386000 | 4.00353500 |
| H | -0.81132600 | 0.66726600 | 2.56208600 |
| C | 1.09173400 | 1.45716800 | 3.26338200 |
| H | 0.94053600 | 2.46706000 | 2.86150600 |
| H | 0.78751400 | 1.48738800 | 4.32517400 |
| H | 2.16342800 | 1.21436900 | 3.24105400 |
| C | -2.67198400 | 0.21831600 | 0.39850200 |
| C | -3.16966200 | 1.18380000 | -0.46794700 |
| C | -4.29368200 | 1.90242500 | -0.06803400 |
| C | -4.89048900 | 1.64781900 | 1.16424200 |
| C | -4.37465200 | 0.66867000 | 2.00946300 |
| C | -3.25167800 | -0.06327600 | 1.63092700 |
| H | -2.69318100 | 1.38154300 | -1.42634500 |
| H | -4.69972500 | 2.66659400 | -0.72878500 |
| H | -5.76797400 | 2.21604600 | 1.46781200 |
| H | -4.84648000 | 0.46631500 | 2.96941900 |
| H | -2.84503300 | -0.83713500 | 2.28045600 |
| I | -0.98999700 | -0.93522700 | -0.19954700 |
| O | -2.77388100 | -2.47871400 | -0.92911700 |
| C | -3.26818600 | -2.20715300 | -2.08851200 |
| O | -2.89025900 | -1.29744300 | -2.83691900 |
| O | -0.08753100 | 2.47756200 | 0.37573400 |
| C | -0.48374900 | 2.83723300 | -0.80075600 |
| O | -0.29259300 | 2.19282400 | -1.84062300 |
| C | -1.19639600 | 4.16630400 | -0.83291900 |
| H | -0.44883000 | 4.97092700 | -0.85805400 |
| H | -1.81847600 | 4.24513000 | -1.73115500 |
| H | -1.81010500 | 4.31034700 | 0.06347000 |
| C | -4.40373700 | -3.12238500 | -2.49855300 |
| H | -5.25732700 | -2.97028100 | -1.82579600 |
| H | -4.71506200 | -2.91615800 | -3.52764600 |
| H | -4.10006300 | -4.17168000 | -2.40621100 |

**$TS_{40\text{-}41}$**

E (M06-SMD/BS1) = -1478.672267 au
H (M06-SMD/BS1) = -1478.183104 au
G (M06-SMD/BS1) = -1478.287105 au
E (M06-SMD/BS2//M06-SMD/BS1) = -1765.611033 au

| | | | |
|---|---|---|---|
| Pd | 0.98264400 | -0.50244100 | -0.60447600 |

| | | | |
|---|---|---|---|
| C | 0.33886500 | 0.03692900 | -2.55245700 |
| C | 0.57604900 | 1.51857900 | -2.68841800 |
| H | -0.34795100 | 2.09114800 | -2.52934700 |
| C | 1.62433200 | 2.07135400 | -1.73467100 |
| C | 3.04674900 | 1.45930700 | 0.22976200 |
| C | 2.40034600 | 2.30323900 | 1.33138400 |
| H | 2.02560400 | 3.24393000 | 0.90728600 |
| H | 3.12403900 | 2.54409700 | 2.12153200 |
| H | 1.55980100 | 1.76135900 | 1.78914000 |
| C | 4.27473300 | 2.16340300 | -0.35780200 |
| H | 4.70475900 | 1.56775700 | -1.17469000 |
| H | 5.04375500 | 2.29849400 | 0.41172100 |
| H | 4.00498900 | 3.14768500 | -0.74575800 |
| C | 3.49472200 | 0.13103200 | 0.82942900 |
| C | 4.55260700 | 0.05410300 | 1.73447900 |
| H | 5.08207400 | 0.95462000 | 2.03731500 |
| C | 4.92827800 | -1.18010300 | 2.24423500 |
| H | 5.75703800 | -1.24746600 | 2.94642700 |
| C | 4.24601100 | -2.32667000 | 1.84716400 |
| H | 4.51886500 | -3.31130200 | 2.21738800 |
| C | 3.18793100 | -2.18127800 | 0.96930800 |
| H | 2.60200600 | -3.03492400 | 0.63078800 |
| N | 2.05469900 | 1.16481600 | -0.82614200 |
| O | 2.00763900 | 3.24198200 | -1.83860600 |
| N | 2.82453100 | -0.98425100 | 0.48639300 |
| H | 0.91678100 | 1.74483300 | -3.71237600 |
| H | -0.71148500 | -0.27350600 | -2.59553300 |
| C | 1.25268100 | -0.85615600 | -3.32994400 |
| H | 1.15234500 | -1.91433600 | -3.05627700 |
| H | 0.96522400 | -0.77422100 | -4.39421500 |
| H | 2.30534900 | -0.55331100 | -3.25086700 |
| C | -2.70844200 | -0.23475300 | -0.53420900 |
| C | -3.15827700 | -1.36416300 | 0.14038000 |
| C | -4.21436500 | -2.08217900 | -0.41453100 |
| C | -4.79846900 | -1.66472300 | -1.60851500 |
| C | -4.33523700 | -0.52425900 | -2.25877500 |
| C | -3.27717200 | 0.20769000 | -1.72368400 |
| H | -2.70085000 | -1.67907300 | 1.07698200 |
| H | -4.57931400 | -2.97231100 | 0.09547200 |
| H | -5.62419200 | -2.23217300 | -2.03394300 |
| H | -4.79699400 | -0.19486700 | -3.18779800 |

| | | | |
|---|---|---|---|
| H | -2.91271700 | 1.10471900 | -2.22174600 |
| I | -1.11465100 | 0.89417500 | 0.30463500 |
| O | -3.09162600 | 2.16881500 | 1.27937400 |
| C | -3.58166900 | 1.63189800 | 2.33759100 |
| O | -3.14187600 | 0.62234500 | 2.91039200 |
| O | -0.00246700 | -2.32671800 | -0.57182200 |
| C | -0.30553300 | -2.82791000 | 0.58139600 |
| O | -0.08566200 | -2.27422500 | 1.66719700 |
| C | -0.93677800 | -4.19477500 | 0.51912700 |
| H | -0.13918000 | -4.94905500 | 0.47133000 |
| H | -1.53199600 | -4.38312100 | 1.41893500 |
| H | -1.55889500 | -4.30976800 | -0.37524700 |
| C | -4.81504600 | 2.33182200 | 2.87685900 |
| H | -5.63374100 | 2.23943800 | 2.15177500 |
| H | -5.12895000 | 1.89416400 | 3.83004500 |
| H | -4.61809900 | 3.40232700 | 3.00924900 |

**41**

E (M06-SMD/BS1) = -1478.684728 au
H (M06-SMD/BS1) = -1478.194124 au
G (M06-SMD/BS1) = -1478.299778 au
E (M06-SMD/BS2//M06-SMD/BS1) = -1765.621111 au

| | | | |
|---|---|---|---|
| Pd | 0.94236000 | 0.17569300 | 0.12181300 |
| C | 0.87816700 | -0.08922400 | 2.26591800 |
| C | 1.18962100 | -1.54574100 | 2.42687400 |
| H | 0.30191400 | -2.16605100 | 2.24796400 |
| C | 2.29320500 | -1.93162200 | 1.45297800 |
| C | 3.04346400 | -1.45210800 | -0.87711200 |
| C | 2.33125700 | -1.43667600 | -2.23599300 |
| H | 1.84290500 | -2.40759100 | -2.39650600 |
| H | 3.06037700 | -1.27281800 | -3.04061300 |
| H | 1.56669900 | -0.65110500 | -2.31246000 |
| C | 4.03179900 | -2.61534900 | -0.89860900 |
| H | 4.75374500 | -2.58040700 | -0.07983000 |
| H | 4.57521700 | -2.60928900 | -1.85272600 |
| H | 3.47970500 | -3.56136100 | -0.83729300 |
| C | 3.71016300 | -0.10175600 | -0.66476700 |
| C | 5.01951700 | 0.20369700 | -1.01102300 |
| H | 5.66495700 | -0.56570100 | -1.42846100 |
| C | 5.48908100 | 1.49820400 | -0.81726700 |
| H | 6.51560400 | 1.74741100 | -1.07818800 |

| | | | |
|---|---|---|---|
| C | 4.64376300 | 2.46956200 | -0.29281000 |
| H | 4.97613500 | 3.49178100 | -0.13422600 |
| C | 3.34550400 | 2.11092500 | 0.02950500 |
| H | 2.63007900 | 2.82325600 | 0.43787200 |
| N | 2.00140800 | -1.55580600 | 0.17256600 |
| O | 3.30920700 | -2.51464700 | 1.82588800 |
| N | 2.91164800 | 0.85877000 | -0.15059200 |
| H | 1.54683600 | -1.72965300 | 3.45071400 |
| H | -0.17378500 | 0.20267300 | 2.37041000 |
| C | 1.84742300 | 0.87310600 | 2.84309600 |
| H | 1.68265900 | 1.90305900 | 2.50699600 |
| H | 1.66432100 | 0.86257300 | 3.93334000 |
| H | 2.89240300 | 0.57752200 | 2.68347100 |
| C | -2.72103200 | 0.38811100 | 0.50323100 |
| C | -3.14128200 | 1.37749400 | -0.37779200 |
| C | -4.02277400 | 2.34731300 | 0.09518600 |
| C | -4.46675200 | 2.31069900 | 1.41522500 |
| C | -4.03559000 | 1.30345200 | 2.27505000 |
| C | -3.15104300 | 0.32569100 | 1.82353000 |
| H | -2.79214400 | 1.39554500 | -1.40911500 |
| H | -4.36100800 | 3.13412900 | -0.57728000 |
| H | -5.15629400 | 3.07184000 | 1.77597000 |
| H | -4.38726300 | 1.27144900 | 3.30477500 |
| H | -2.81393800 | -0.47032700 | 2.48567100 |
| I | -1.38444900 | -1.11119900 | -0.19402100 |
| O | -4.16469000 | -2.23274800 | -0.39843100 |
| C | -4.76869500 | -1.49130900 | -1.22822700 |
| O | -4.30633900 | -1.08391700 | -2.32169000 |
| O | 0.05786600 | 2.05522500 | 0.18862800 |
| C | -0.05058600 | 2.47417300 | -1.02814800 |
| O | 0.30655300 | 1.79021800 | -2.00299000 |
| C | -0.60328300 | 3.85851500 | -1.20268000 |
| H | 0.23407700 | 4.55126300 | -1.36313300 |
| H | -1.24325200 | 3.89877800 | -2.09120300 |
| H | -1.16164400 | 4.18896400 | -0.32103600 |
| C | -6.15866100 | -1.02066900 | -0.82448100 |
| H | -6.05528600 | -0.19377400 | -0.10554600 |
| H | -6.73030600 | -0.65475500 | -1.68483200 |
| H | -6.71722000 | -1.81850500 | -0.32020900 |

**$TS_{41\text{-}42}$**

E (M06-SMD/BS1) = -1594.359776 au
H (M06-SMD/BS1) = -1593.81243 au
G (M06-SMD/BS1) = -1593.923189 au
E (M06-SMD/BS2//M06-SMD/BS1) = -1881.344118 au

| | | | |
|---|---|---|---|
| Pd | -0.66778700 | -0.12458800 | 0.05936500 |
| C | -0.69931700 | 2.21809700 | 0.44470100 |
| C | -1.14739000 | 2.61193800 | -0.91623300 |
| H | -0.32007900 | 2.57029800 | -1.63640600 |
| C | -2.30343500 | 1.71158100 | -1.36036600 |
| C | -2.97389400 | -0.67428500 | -1.47936700 |
| C | -2.26610100 | -1.96150300 | -1.92492600 |
| H | -1.90882900 | -1.83683000 | -2.95662800 |
| H | -2.96829200 | -2.80530300 | -1.89490800 |
| H | -1.40380600 | -2.21785400 | -1.29205800 |
| C | -4.10989000 | -0.41578900 | -2.46437100 |
| H | -4.81562600 | 0.34040700 | -2.11437700 |
| H | -4.65216600 | -1.35283700 | -2.64784900 |
| H | -3.68896200 | -0.08250800 | -3.42147500 |
| C | -3.44484700 | -0.85422800 | -0.04639400 |
| C | -4.70768000 | -1.28430000 | 0.33577500 |
| H | -5.46384700 | -1.48270500 | -0.42015800 |
| C | -4.98663200 | -1.45502200 | 1.68742700 |
| H | -5.97461200 | -1.78688900 | 1.99999500 |
| C | -4.00047600 | -1.20145100 | 2.63424400 |
| H | -4.18331400 | -1.32782100 | 3.69781500 |
| C | -2.75658100 | -0.77742600 | 2.19743100 |
| H | -1.94009600 | -0.56310000 | 2.88543900 |
| N | -1.95180500 | 0.39940700 | -1.42519600 |
| O | -3.41096000 | 2.18747100 | -1.60625000 |
| N | -2.50562300 | -0.61045900 | 0.89387000 |
| H | -1.52788800 | 3.64219800 | -0.86949600 |
| H | 0.37165700 | 2.11740000 | 0.66023100 |
| C | -1.61164500 | 2.39152400 | 1.58542700 |
| H | -1.30583100 | 1.81365500 | 2.46453100 |
| H | -1.53649400 | 3.45949100 | 1.85829100 |
| H | -2.66388900 | 2.20698400 | 1.33374100 |
| C | 2.51124700 | -1.68351800 | -0.72468600 |
| C | 2.33055000 | -2.93034500 | -1.31148300 |
| C | 2.95891000 | -4.02875300 | -0.72881700 |
| C | 3.74119900 | -3.86608100 | 0.41169400 |
| C | 3.90620700 | -2.60429900 | 0.97754500 |

| | | | |
|---|---|---|---|
| C | 3.28730800 | -1.49113300 | 0.41143800 |
| H | 1.70474800 | -3.04850000 | -2.19476900 |
| H | 2.82963300 | -5.01538000 | -1.17052400 |
| H | 4.22616300 | -4.72965200 | 0.86344100 |
| H | 4.51489200 | -2.47881400 | 1.87179900 |
| H | 3.38694100 | -0.50264700 | 0.85979300 |
| I | 1.50073700 | -0.00978100 | -1.57663600 |
| O | 2.66006000 | 3.35164900 | -0.40598400 |
| C | 3.15668600 | 2.49985500 | 0.39037600 |
| O | 2.53549100 | 1.92884000 | 1.32347900 |
| O | 0.42946500 | -0.59914600 | 1.75011000 |
| C | 0.50236700 | -1.87410400 | 1.96016100 |
| O | 0.01870400 | -2.72764700 | 1.20264200 |
| C | 1.17432500 | -2.25975600 | 3.24962500 |
| H | 0.43187200 | -2.19767000 | 4.05798400 |
| H | 1.54334300 | -3.28940600 | 3.20090300 |
| H | 1.99245900 | -1.57420700 | 3.49681000 |
| C | 4.60583700 | 2.12012500 | 0.15352100 |
| H | 5.18572200 | 2.97796300 | -0.20667200 |
| H | 5.07030500 | 1.71052000 | 1.05784500 |
| H | 4.64518800 | 1.34641500 | -0.62832800 |
| C | 0.92915000 | 4.76869700 | 1.99727600 |
| H | 1.93301400 | 5.22244900 | 2.02862900 |
| H | 0.22691000 | 5.46224300 | 2.47683400 |
| H | 0.95947000 | 3.84153900 | 2.59518700 |
| O | 0.50234200 | 4.54178100 | 0.67388800 |
| H | 1.25636700 | 4.08485300 | 0.21150200 |

**42**

E (M06-SMD/BS1) = -1365.443307 au
H (M06-SMD/BS1) = -1364.961359 au
G (M06-SMD/BS1) = -1365.058313 au
E (M06-SMD/BS2//M06-SMD/BS1) = -1652.328603 au

| | | | |
|---|---|---|---|
| Pd | -0.29635400 | -0.12985400 | 0.08968900 |
| C | -1.77171800 | 2.69533800 | 0.87604500 |
| C | -2.05844300 | 2.47700700 | -0.60770800 |
| H | -1.14205100 | 2.67952900 | -1.17664700 |
| C | -2.60355000 | 1.10130200 | -0.94439500 |
| C | -2.18990500 | -1.17233000 | -1.72763800 |
| C | -1.02260300 | -1.95114900 | -2.35145300 |
| H | -0.69595400 | -1.44061900 | -3.26858400 |

| | | | |
|---|---|---|---|
| H | -1.34335600 | -2.96911900 | -2.61201900 |
| H | -0.15922600 | -2.02708200 | -1.67544900 |
| C | -3.31577600 | -1.15881600 | -2.75792200 |
| H | -4.26300500 | -0.79973600 | -2.34967700 |
| H | -3.46303000 | -2.16659500 | -3.16861800 |
| H | -3.02515600 | -0.50252900 | -3.58933700 |
| C | -2.55707000 | -1.84333900 | -0.42106300 |
| C | -3.57627000 | -2.76690900 | -0.23326100 |
| H | -4.21440900 | -3.04798400 | -1.06814900 |
| C | -3.76953900 | -3.31588200 | 1.02967700 |
| H | -4.56819300 | -4.03688500 | 1.19268500 |
| C | -2.94209500 | -2.93610800 | 2.08128400 |
| H | -3.06713000 | -3.34184200 | 3.08157400 |
| C | -1.93684300 | -2.01610900 | 1.83262400 |
| H | -1.25202700 | -1.67701800 | 2.60849300 |
| N | -1.69199700 | 0.18449500 | -1.38802600 |
| O | -3.82301600 | 0.89908800 | -0.83712300 |
| N | -1.75906500 | -1.49715700 | 0.61178500 |
| H | -2.81720100 | 3.20999800 | -0.92052600 |
| H | -1.03169600 | 1.94178300 | 1.21890900 |
| C | -3.01006200 | 2.59497500 | 1.74313700 |
| H | -2.78064700 | 2.80701300 | 2.79500500 |
| H | -3.77924700 | 3.30296400 | 1.40301600 |
| H | -3.42987000 | 1.58133200 | 1.69750200 |
| C | 3.18599100 | 0.12671700 | -0.62791700 |
| C | 3.36820700 | -0.85595800 | -1.59391500 |
| C | 4.36309400 | -1.80955400 | -1.38693100 |
| C | 5.14391400 | -1.77224900 | -0.23436100 |
| C | 4.94114000 | -0.77727000 | 0.71809700 |
| C | 3.95307500 | 0.18765900 | 0.52865300 |
| H | 2.74210600 | -0.88745900 | -2.48445500 |
| H | 4.52008600 | -2.58779800 | -2.13199100 |
| H | 5.91548300 | -2.52403100 | -0.07787000 |
| H | 5.54936800 | -0.74848800 | 1.62101300 |
| H | 3.77976600 | 0.96050900 | 1.27566700 |
| I | 1.59759400 | 1.53508000 | -0.88430400 |
| O | 0.90072400 | -0.52665500 | 1.73832300 |
| C | 1.53482000 | -1.65393100 | 1.73919000 |
| O | 1.49651200 | -2.48758900 | 0.82324700 |
| C | 2.31277900 | -1.92999300 | 3.00082100 |
| H | 1.63066200 | -2.37241400 | 3.74067300 |

| | | | |
|---|---|---|---|
| H | 3.11870600 | -2.64598800 | 2.80777800 |
| H | 2.72180900 | -1.01066300 | 3.43447400 |
| C | -0.44898300 | 4.18507400 | 2.15733700 |
| H | 0.07187600 | 5.14444700 | 2.06493100 |
| H | -1.10656900 | 4.23118000 | 3.03970700 |
| H | 0.29941800 | 3.38914300 | 2.31941500 |
| O | -1.16099900 | 3.97642800 | 0.96014700 |

**43**

E (M06-SMD/BS1) = -930.4838017 au

H (M06-SMD/BS1) = -930.186073 au

G (M06-SMD/BS1) = -930.251745 au

E (M06-SMD/BS2//M06-SMD/BS1) = -930.8216598 au

| | | | |
|---|---|---|---|
| C | -2.33569200 | 2.15883600 | -0.23277600 |
| C | -1.38558100 | 1.15756800 | -0.07661900 |
| C | -1.85479200 | -0.19380000 | 0.04774100 |
| C | -3.24359800 | -0.48331800 | 0.01152500 |
| C | -4.17305200 | 0.56993600 | -0.14612900 |
| C | -3.71171500 | 1.85795700 | -0.26384600 |
| H | -2.00767600 | 3.18721400 | -0.33211400 |
| C | -3.63535300 | -1.83726900 | 0.13241600 |
| H | -5.23721500 | 0.33883400 | -0.17428700 |
| H | -4.41779900 | 2.67804000 | -0.38841400 |
| C | -2.68893300 | -2.81906800 | 0.28028500 |
| C | -1.33256900 | -2.44689300 | 0.31052400 |
| H | -4.69754300 | -2.08060000 | 0.10488000 |
| H | -2.96271200 | -3.86695700 | 0.37453200 |
| H | -0.54973700 | -3.19743700 | 0.43109800 |
| N | -0.93158800 | -1.19468300 | 0.20068100 |
| N | -0.00316400 | 1.32654700 | -0.02331400 |
| C | 0.66769500 | 2.51120700 | -0.00599200 |
| C | 2.14980200 | 2.30730800 | 0.21137400 |
| H | 2.70592400 | 3.13490500 | -0.25947100 |
| H | 2.31187200 | 2.41675500 | 1.29896100 |
| C | 2.64633700 | 0.93560300 | -0.23514800 |
| H | 2.74942100 | 0.91890100 | -1.33482900 |
| C | 3.95244000 | 0.54638000 | 0.41777100 |
| H | 3.83931800 | 0.41163000 | 1.50488400 |
| H | 4.37363700 | -0.37965600 | 0.00013600 |
| H | 4.71576900 | 1.33201100 | 0.27225800 |
| O | 0.16020800 | 3.63726300 | -0.09702000 |

| | | | |
|---|---|---|---|
| Pd | 1.08706800 | -0.34671900 | 0.07775200 |
| O | 2.23236600 | -2.18035500 | 0.20812200 |
| H | 3.05661800 | -2.02893600 | 0.70109700 |
| C | 2.55498400 | -2.73809700 | -1.07063500 |
| H | 1.60524100 | -2.93905400 | -1.57585200 |
| H | 3.14713100 | -2.03589700 | -1.67242200 |
| H | 3.10547600 | -3.67708800 | -0.94036700 |

**44**

E (M06-SMD/BS1) = -1502.937736 au

H (M06-SMD/BS1) = -1502.433864 au

G (M06-SMD/BS1) = -1502.524366 au

E (M06-SMD/BS2//M06-SMD/BS1) = -1503.479093 au

| | | | |
|---|---|---|---|
| C | -4.03933800 | 0.25381100 | 0.32207300 |
| C | -2.66899600 | 0.41114500 | 0.49274400 |
| C | -1.94720800 | -0.66648500 | 1.11286300 |
| C | -2.62662600 | -1.83241700 | 1.55507300 |
| C | -4.02093800 | -1.94801100 | 1.35332500 |
| C | -4.69680800 | -0.91810300 | 0.74559700 |
| H | -4.60674600 | 1.04999300 | -0.14623400 |
| C | -1.85760800 | -2.84827200 | 2.16996900 |
| H | -4.53204600 | -2.85157800 | 1.68382500 |
| H | -5.77104300 | -0.99604700 | 0.58233500 |
| C | -0.50305400 | -2.69102500 | 2.32330100 |
| C | 0.09466400 | -1.51128400 | 1.84367600 |
| H | -2.36154400 | -3.75087000 | 2.51650900 |
| H | 0.11110200 | -3.45325000 | 2.79628500 |
| H | 1.17168300 | -1.35641400 | 1.94125000 |
| N | -0.59040400 | -0.54517800 | 1.26085600 |
| N | -1.90669000 | 1.51423400 | 0.12216200 |
| C | -2.37229600 | 2.68622700 | -0.38555500 |
| C | -1.25245200 | 3.68498500 | -0.57417700 |
| H | -1.53162600 | 4.40404600 | -1.36225500 |
| H | -1.20399900 | 4.26779500 | 0.36362400 |
| C | 0.10459600 | 3.03405200 | -0.83235900 |
| H | 0.14476500 | 2.68593500 | -1.87930300 |
| C | 1.25688600 | 3.96881200 | -0.55065700 |
| H | 1.29825800 | 4.25445200 | 0.51239500 |
| H | 2.22662700 | 3.52695500 | -0.81787300 |
| H | 1.15410500 | 4.90649100 | -1.12669700 |
| O | -3.55686200 | 2.96267700 | -0.62392400 |

| | | | |
|---|---|---|---|
| Pd | 0.08893200 | 1.28077200 | 0.23764800 |
| C | 2.98611400 | -1.95531000 | -1.72302700 |
| C | 2.35322100 | -0.82097200 | -1.26205300 |
| C | 2.82699700 | -0.17882900 | -0.07807900 |
| C | 3.98413700 | -0.71252200 | 0.55946200 |
| C | 4.59193000 | -1.89014500 | 0.06580300 |
| C | 4.08776900 | -2.51196600 | -1.04674800 |
| H | 2.63589500 | -2.40741400 | -2.64934400 |
| C | 4.49763200 | -0.04303900 | 1.69211900 |
| H | 5.46687300 | -2.27965000 | 0.58490300 |
| H | 4.55390400 | -3.41403100 | -1.43732100 |
| C | 3.88374200 | 1.09438900 | 2.14550800 |
| C | 2.72338500 | 1.53507300 | 1.48956500 |
| H | 5.38340600 | -0.44396600 | 2.18377900 |
| H | 4.25545100 | 1.64625000 | 3.00473400 |
| H | 2.19393200 | 2.41514600 | 1.85348900 |
| N | 2.19258900 | 0.92519300 | 0.43693900 |
| N | 1.29425900 | -0.25058600 | -2.00152800 |
| H | 1.36057400 | 0.74601400 | -2.20031900 |
| C | 0.06791900 | -0.78189100 | -2.29588500 |
| C | -0.22194100 | -2.19502200 | -1.86219600 |
| H | 0.34550400 | -2.88399900 | -2.50817400 |
| H | 0.17492800 | -2.35197900 | -0.84544000 |
| C | -1.70262800 | -2.53785700 | -1.92050800 |
| H | -2.06220500 | -2.43317000 | -2.95410300 |
| H | -2.27168400 | -1.80702500 | -1.32389800 |
| C | -1.96915200 | -3.94261400 | -1.41169600 |
| H | -1.60691600 | -4.06605900 | -0.37961800 |
| H | -3.04232700 | -4.17466400 | -1.41746100 |
| H | -1.45950700 | -4.69564800 | -2.03012400 |
| O | -0.75891200 | -0.09453400 | -2.89790300 |

**45**

E (M06-SMD/BS1) = -1502.919763 au

H (M06-SMD/BS1) = -1502.415861 au

G (M06-SMD/BS1) = -1502.510273 au

E (M06-SMD/BS2//M06-SMD/BS1) = -1503.461371 au

| | | | |
|---|---|---|---|
| C | -5.62935000 | -0.26338800 | 0.06070800 |
| C | -4.26184400 | -0.09383800 | -0.11638800 |
| C | -3.74630000 | 1.24612300 | -0.08131900 |
| C | -4.61514200 | 2.35058400 | 0.12071700 |

| | | | |
|---|---|---|---|
| C | -6.00057200 | 2.12765000 | 0.29129900 |
| C | -6.48037900 | 0.84159700 | 0.26003600 |
| H | -6.04420800 | -1.26475400 | 0.04428100 |
| C | -4.03947200 | 3.64259000 | 0.14658800 |
| H | -6.66011800 | 2.98033300 | 0.44812000 |
| H | -7.54538700 | 0.65611900 | 0.39440700 |
| C | -2.68708500 | 3.79861500 | -0.02003100 |
| C | -1.89629400 | 2.65161000 | -0.21559700 |
| H | -4.68942100 | 4.50394700 | 0.30097000 |
| H | -2.21919100 | 4.77982300 | -0.00485600 |
| H | -0.81738100 | 2.73570200 | -0.35488300 |
| N | -2.39887000 | 1.43225200 | -0.24627600 |
| N | -3.32154400 | -1.09955900 | -0.33028600 |
| C | -3.58443700 | -2.41792200 | -0.53960100 |
| C | -2.33095300 | -3.18261500 | -0.89726200 |
| H | -2.44000200 | -4.23401200 | -0.58283500 |
| H | -2.29535600 | -3.20107200 | -2.00160500 |
| C | -1.05319800 | -2.54021100 | -0.36479900 |
| H | -0.95231500 | -2.76193600 | 0.71276700 |
| C | 0.17666300 | -2.99882800 | -1.11465500 |
| H | 0.17019000 | -2.64595100 | -2.15765100 |
| H | 1.11177600 | -2.65343600 | -0.65128300 |
| H | 0.22544600 | -4.10231800 | -1.14893600 |
| O | -4.70115000 | -2.95398000 | -0.50805100 |
| Pd | -1.40280700 | -0.52425400 | -0.38276300 |
| C | 4.40808000 | -1.44270100 | 0.90780000 |
| C | 4.13365000 | -0.39662700 | 0.05519600 |
| C | 5.18994600 | 0.43723300 | -0.41777900 |
| C | 6.51016500 | 0.19606000 | 0.04926500 |
| C | 6.75422300 | -0.85511500 | 0.96410300 |
| C | 5.72077700 | -1.65753800 | 1.37736500 |
| H | 3.61324600 | -2.12333100 | 1.20737500 |
| C | 7.53334900 | 1.04087500 | -0.43944600 |
| H | 7.77158100 | -1.01953600 | 1.31804400 |
| H | 5.90520400 | -2.48251500 | 2.06265600 |
| C | 7.22131000 | 2.03217600 | -1.33334500 |
| C | 5.87811600 | 2.17517600 | -1.74117000 |
| H | 8.55686800 | 0.88552600 | -0.09826900 |
| H | 7.98192000 | 2.69949400 | -1.73187000 |
| H | 5.61643200 | 2.95453500 | -2.45966400 |
| N | 4.89110600 | 1.41928600 | -1.30776400 |

| | | | |
|---|---|---|---|
| N | 2.84875600 | -0.13576700 | -0.45807200 |
| H | 2.83691300 | 0.24562400 | -1.40440600 |
| C | 1.63520400 | -0.16447300 | 0.13227100 |
| C | 1.50993700 | -0.44853800 | 1.59897500 |
| H | 1.26739600 | -1.51794000 | 1.72073800 |
| H | 2.48150200 | -0.29722200 | 2.08744700 |
| C | 0.45353000 | 0.40331600 | 2.29644900 |
| H | -0.53987600 | 0.18592500 | 1.87320300 |
| H | 0.65355000 | 1.46812600 | 2.09910900 |
| C | 0.44657200 | 0.13732800 | 3.79073100 |
| H | 1.41899900 | 0.37484900 | 4.24498100 |
| H | -0.31603000 | 0.74070100 | 4.29922700 |
| H | 0.23207400 | -0.92023300 | 4.00198200 |
| O | 0.64992200 | 0.09159200 | -0.59643000 |

**$TS^{OMe}_{18-19}$**

E (M06-SMD/BS1) = -1360.53033543 au
H (M06-SMD/BS1) = -1360.143034 au
G (M06-SMD/BS1) = -1360.230451 au
E (M06-SMD/BS2//M06-SMD/BS1) = -1647.41281255 au

| | | | |
|---|---|---|---|
| C | -3.59499100 | 1.25130200 | 1.50901800 |
| C | -2.61915600 | 0.73667600 | 0.66745100 |
| C | -2.16198500 | 1.55457300 | -0.41811300 |
| C | -2.70687700 | 2.85124700 | -0.61266700 |
| C | -3.70084500 | 3.33461600 | 0.26646600 |
| C | -4.12449600 | 2.53989700 | 1.30097600 |
| H | -3.95782500 | 0.64902200 | 2.33279800 |
| C | -2.21949000 | 3.60898000 | -1.70378600 |
| H | -4.11400200 | 4.32913500 | 0.10441600 |
| H | -4.89143500 | 2.89726000 | 1.98612900 |
| C | -1.25934600 | 3.08989400 | -2.53375700 |
| C | -0.77209900 | 1.79649200 | -2.26950900 |
| H | -2.62363800 | 4.60756700 | -1.86844700 |
| H | -0.87109600 | 3.65058300 | -3.38006800 |
| H | -0.01062700 | 1.34455500 | -2.90720500 |
| N | -1.20128200 | 1.06989400 | -1.25840000 |
| N | -2.04673200 | -0.53738600 | 0.80295600 |
| C | -2.24967600 | -1.45868900 | 1.80152600 |
| C | -1.32827000 | -2.64915400 | 1.69655500 |
| H | -1.84272300 | -3.53369200 | 2.10097300 |
| H | -0.46938200 | -2.45817600 | 2.36078100 |

| | | | |
|---|---|---|---|
| C | -0.87250400 | -2.90379200 | 0.27872200 |
| H | -1.68982700 | -3.33305000 | -0.31316000 |
| C | 0.42045600 | -3.63264600 | 0.08917300 |
| H | 1.24505800 | -3.16861400 | 0.64725000 |
| H | 0.69908900 | -3.69795000 | -0.97263000 |
| H | 0.31101400 | -4.66758300 | 0.45760800 |
| O | -3.06786900 | -1.31301100 | 2.70594800 |
| Pd | -0.73542700 | -1.04220500 | -0.62179800 |
| C | 4.86265200 | 0.81036000 | -0.79242200 |
| C | 3.82003400 | 0.73266400 | 0.13434200 |
| C | 2.65359600 | 0.08876600 | -0.26689300 |
| C | 2.50868200 | -0.48121400 | -1.52451100 |
| C | 3.56781300 | -0.38812700 | -2.42577100 |
| C | 4.74278100 | 0.26101200 | -2.06144200 |
| H | 5.77725400 | 1.31438700 | -0.48509500 |
| H | 1.59806600 | -0.99967600 | -1.82803200 |
| H | 3.46331800 | -0.83080500 | -3.41474000 |
| H | 5.56876100 | 0.33321500 | -2.76640800 |
| I | 1.02969700 | 0.00412400 | 1.14495000 |
| C | 4.00715500 | 1.32115400 | 1.51367300 |
| O | 5.06606200 | 1.92004600 | 1.76624900 |
| O | 3.04660600 | 1.14512900 | 2.32863600 |
| O | -1.47141300 | -1.70359300 | -2.36946400 |
| C | -2.01365800 | -2.95119000 | -2.66367700 |
| H | -1.35840700 | -3.79640700 | -2.38670600 |
| H | -2.19496800 | -3.01104400 | -3.75012100 |
| H | -2.99258100 | -3.09892500 | -2.17199700 |

**$19^{OMe}$**

E (M06-SMD/BS1) = -1360.53508673 au

H (M06-SMD/BS1) = -1360.146486 au

G (M06-SMD/BS1) = -1360.235246 au

E (M06-SMD/BS2//M06-SMD/BS1) = -1647.41641234 au

| | | | |
|---|---|---|---|
| C | -4.16559600 | 0.39003700 | -1.35367700 |
| C | -2.97579600 | 0.14942600 | -0.68253900 |
| C | -2.69406700 | -1.18960200 | -0.25264300 |
| C | -3.60164400 | -2.23711300 | -0.56008900 |
| C | -4.78882500 | -1.95330100 | -1.27120400 |
| C | -5.05553500 | -0.66129200 | -1.64643100 |
| H | -4.39912000 | 1.39700600 | -1.67840300 |
| C | -3.26920900 | -3.54048300 | -0.12273000 |

| | | | |
|---|---|---|---|
| H | -5.47441700 | -2.76712700 | -1.50281100 |
| H | -5.97049900 | -0.42602400 | -2.18743200 |
| C | -2.11065500 | -3.75996100 | 0.57746300 |
| C | -1.27740700 | -2.66133100 | 0.85759400 |
| H | -3.95077900 | -4.35910300 | -0.35256000 |
| H | -1.83029900 | -4.75180000 | 0.92234200 |
| H | -0.35170300 | -2.79380300 | 1.41995300 |
| N | -1.55988700 | -1.43578400 | 0.46559100 |
| N | -2.01776800 | 1.13468900 | -0.40721700 |
| C | -1.98477000 | 2.44228600 | -0.82884400 |
| C | -0.81078900 | 3.21280300 | -0.27910800 |
| H | -1.10158700 | 4.26742800 | -0.16298000 |
| H | -0.01471400 | 3.18514300 | -1.04071800 |
| C | -0.31589700 | 2.65734100 | 1.03182800 |
| H | -1.01238800 | 2.86092100 | 1.85437000 |
| C | 1.10993500 | 2.89724000 | 1.40072900 |
| H | 1.80454400 | 2.69424400 | 0.57617100 |
| H | 1.41222200 | 2.31818500 | 2.28485700 |
| H | 1.21756000 | 3.96139400 | 1.67715000 |
| O | -2.79424800 | 2.91371900 | -1.62106400 |
| Pd | -0.63062200 | 0.59559800 | 0.90009400 |
| C | 5.06366000 | -1.07904200 | 0.53080800 |
| C | 4.06263200 | -0.66399100 | -0.35422100 |
| C | 2.77639900 | -0.52947000 | 0.16273700 |
| C | 2.47956100 | -0.79825200 | 1.49499400 |
| C | 3.49729700 | -1.22055900 | 2.34652300 |
| C | 4.79331900 | -1.36108300 | 1.86236600 |
| H | 6.07216300 | -1.17936000 | 0.13387000 |
| H | 1.46869600 | -0.69694300 | 1.89304000 |
| H | 3.26557300 | -1.43898400 | 3.38758800 |
| H | 5.59268700 | -1.69140700 | 2.52317700 |
| I | 1.17996300 | 0.14733800 | -1.11641200 |
| C | 4.43914600 | -0.41233400 | -1.80627900 |
| O | 5.65482900 | -0.31273400 | -2.07006700 |
| O | 3.48473900 | -0.33847000 | -2.62855600 |
| O | -1.99450400 | 0.80846300 | 2.36479400 |
| C | -1.28700200 | 0.44311900 | 3.50759400 |
| H | -0.86529900 | -0.58052300 | 3.43793000 |
| H | -1.98831900 | 0.43455800 | 4.36097700 |
| H | -0.47525300 | 1.15295000 | 3.76026800 |

**$TS^{\alpha}_{15\text{-}16}$**

E (M06-SMD/BS1) = -1043.75477998 au

H (M06-SMD/BS1) = -1043.451449 au

G (M06-SMD/BS1) = -1043.521142 au

E (M06-SMD/BS2//M06-SMD/BS1) = -1044.13672027 au

| | | | |
|---|---|---|---|
| C | 2.66119500 | -2.26790100 | -0.03478000 |
| C | 1.78901600 | -1.21416100 | 0.15640000 |
| C | 2.28661400 | 0.11643800 | 0.08412100 |
| C | 3.65918600 | 0.36673600 | -0.14329800 |
| C | 4.52893600 | -0.73497300 | -0.32355200 |
| C | 4.02802700 | -2.01464400 | -0.27815800 |
| H | 2.29563600 | -3.29044800 | 0.02191400 |
| C | 4.07189300 | 1.71840000 | -0.20164900 |
| H | 5.58549400 | -0.54793700 | -0.50954200 |
| H | 4.69584000 | -2.86184200 | -0.42489500 |
| C | 3.15210400 | 2.72647400 | -0.05005600 |
| C | 1.79961500 | 2.39843400 | 0.15573600 |
| H | 5.12419800 | 1.94310000 | -0.37337200 |
| H | 3.44295900 | 3.77263400 | -0.09312400 |
| H | 1.04021200 | 3.17114400 | 0.26743700 |
| N | 1.38348300 | 1.14659900 | 0.21844700 |
| N | 0.44003000 | -1.30578300 | 0.50389800 |
| C | -0.51183400 | -2.12512600 | -0.05053200 |
| C | -1.85931000 | -1.52818500 | 0.30564000 |
| O | -0.31334000 | -3.12138200 | -0.74114600 |
| Pd | -0.55290300 | 0.37252400 | 0.31196300 |
| O | -1.68473000 | 2.12309300 | 0.04531300 |
| C | -2.87030800 | 1.86414800 | -0.32048200 |
| O | -3.31232700 | 0.68215200 | -0.45433200 |
| C | -3.80320100 | 2.99592100 | -0.60476900 |
| H | -4.65352200 | 2.94313000 | 0.08607900 |
| H | -4.20247600 | 2.89350500 | -1.62072000 |
| H | -3.30412400 | 3.96272800 | -0.49884000 |
| H | -2.38264600 | -0.21932400 | -0.04967300 |
| C | -3.02919000 | -2.20828000 | -0.41199700 |
| H | -3.15370900 | -1.79660800 | -1.42477300 |
| H | -2.74689000 | -3.26256200 | -0.54853000 |
| C | -4.33303600 | -2.13426200 | 0.36205800 |
| H | -4.22853700 | -2.61487700 | 1.34518600 |
| H | -5.13771900 | -2.65151500 | -0.17656500 |
| H | -4.65602000 | -1.09878500 | 0.52861000 |

| | | | |
|---|---|---|---|
| H | -1.98481200 | -1.58918200 | 1.39731700 |

**$TS^{Y}_{15-16}$**

E (M06-SMD/BS1) = -1043.76169994 au
H (M06-SMD/BS1) = -1043.459012 au
G (M06-SMD/BS1) = -1043.525367 au
E (M06-SMD/BS2//M06-SMD/BS1) = -1044.14399250 au

| | | | |
|---|---|---|---|
| C | -3.02646400 | 1.29846000 | -0.74958700 |
| C | -1.85571200 | 0.73534700 | -0.26094500 |
| C | -1.87608000 | -0.65818700 | 0.01714800 |
| C | -3.05417100 | -1.43419000 | -0.12070700 |
| C | -4.22976500 | -0.81112000 | -0.59338700 |
| C | -4.19329200 | 0.52437600 | -0.91243600 |
| H | -3.05212800 | 2.35073000 | -1.00658500 |
| C | -2.97698500 | -2.80507900 | 0.21394600 |
| H | -5.13724600 | -1.40226900 | -0.70686500 |
| H | -5.08640800 | 1.01402500 | -1.29778200 |
| C | -1.78991600 | -3.34584100 | 0.64203100 |
| C | -0.65569800 | -2.52098300 | 0.72096800 |
| H | -3.87163900 | -3.42046000 | 0.12252200 |
| H | -1.70344300 | -4.39620900 | 0.90699600 |
| H | 0.31199200 | -2.91509600 | 1.02691400 |
| N | -0.70383200 | -1.23543500 | 0.41804400 |
| N | -0.62946100 | 1.39504100 | -0.04665200 |
| C | -0.61538800 | 2.72032900 | 0.26433700 |
| C | 1.92267100 | 2.89652200 | -0.29014600 |
| C | 2.46552800 | 1.58241200 | 0.23057700 |
| H | 2.43167200 | 1.53142700 | 1.33212000 |
| O | -1.63822100 | 3.40725300 | 0.40783600 |
| Pd | 0.89529000 | 0.05607900 | 0.26075700 |
| O | 2.29810000 | -1.49219500 | 0.45752800 |
| C | 3.14854100 | -1.47474600 | -0.48548200 |
| O | 3.24605300 | -0.49481600 | -1.28667700 |
| C | 4.05121400 | -2.64822500 | -0.66760500 |
| H | 5.07818700 | -2.30663000 | -0.83551800 |
| H | 3.73504500 | -3.19652000 | -1.56445700 |
| H | 4.01010300 | -3.32020400 | 0.19357800 |
| H | 2.58351100 | 0.48686000 | -0.65447500 |
| H | 3.54618100 | 1.50610800 | 0.02252100 |
| C | 0.71825800 | 3.39493300 | 0.48610300 |
| H | 0.93386400 | 3.33835500 | 1.56712400 |

| | | | |
|---|---|---|---|
| H | 0.53997900 | 4.45677500 | 0.27259500 |
| H | 1.67995600 | 2.82387800 | -1.36175700 |
| H | 2.70617100 | 3.66635000 | -0.20987400 |

**16$^{\alpha}$**

E (M06-SMD/BS1) = -1043.78400239 au
H (M06-SMD/BS1) = -1043.475339 au
G (M06-SMD/BS1) = -1043.547544 au
E (M06-SMD/BS2//M06-SMD/BS1) = -1044.16695403 au

| | | | |
|---|---|---|---|
| C | -2.91889700 | 2.00765300 | -0.15374300 |
| C | -1.92284400 | 1.10358400 | 0.16613800 |
| C | -2.21648800 | -0.29520600 | 0.12078800 |
| C | -3.52126400 | -0.73495400 | -0.21953700 |
| C | -4.51805200 | 0.22063300 | -0.52734200 |
| C | -4.20980000 | 1.55970900 | -0.50031300 |
| H | -2.70430700 | 3.07373800 | -0.11413200 |
| C | -3.75032500 | -2.13062300 | -0.25982400 |
| H | -5.51560400 | -0.12553700 | -0.79515100 |
| H | -4.97245100 | 2.29771500 | -0.74413500 |
| C | -2.72757000 | -3.00247400 | 0.01449200 |
| C | -1.45856500 | -2.48185300 | 0.33306200 |
| H | -4.74477600 | -2.49471200 | -0.51771500 |
| H | -2.87443300 | -4.07928100 | -0.01301600 |
| H | -0.62292000 | -3.14841600 | 0.54983200 |
| N | -1.20546800 | -1.18805900 | 0.38603900 |
| N | -0.65003400 | 1.45823400 | 0.61857500 |
| C | 0.22086900 | 2.30076500 | -0.05438400 |
| C | 1.56143800 | 1.89161700 | 0.44269900 |
| O | -0.04586400 | 3.06393300 | -0.98800200 |
| Pd | 0.68319300 | -0.01636400 | 0.57433600 |
| O | 2.27075700 | -1.49228400 | 0.52510300 |
| C | 2.87882200 | -1.78536800 | -0.50768800 |
| O | 2.62278400 | -1.22289100 | -1.67274400 |
| C | 3.98026700 | -2.77711900 | -0.54863800 |
| H | 4.92336600 | -2.24532800 | -0.73224000 |
| H | 3.83021300 | -3.47603400 | -1.37885600 |
| H | 4.04466600 | -3.31607400 | 0.39905300 |
| H | 1.89980600 | -0.56302400 | -1.56650200 |
| C | 2.74915900 | 2.18116700 | -0.43405000 |
| H | 2.58334700 | 1.75690200 | -1.43820300 |
| H | 2.81932300 | 3.27363100 | -0.58451500 |

| | | | |
|---|---|---|---|
| C | 4.05471900 | 1.67114100 | 0.15050000 |
| H | 4.25586100 | 2.13933200 | 1.12492400 |
| H | 4.90539000 | 1.89207500 | -0.50695400 |
| H | 4.02847300 | 0.58379600 | 0.31329000 |
| H | 1.71546100 | 2.13065000 | 1.50475200 |

**16$^{\gamma}$**

E (M06-SMD/BS1) = -1043.79871674 au
H (M06-SMD/BS1) = -1043.489349 au
G (M06-SMD/BS1) = -1043.559273 au
E (M06-SMD/BS2//M06-SMD/BS1) = -1044.18064468 au

| | | | |
|---|---|---|---|
| C | -3.22979900 | 0.74858100 | -0.62192500 |
| C | -1.95010800 | 0.43021100 | -0.18799600 |
| C | -1.67128500 | -0.94693900 | 0.07435900 |
| C | -2.68108300 | -1.93698000 | -0.05782300 |
| C | -3.97490200 | -1.55876700 | -0.47852200 |
| C | -4.22464200 | -0.23808300 | -0.76231800 |
| H | -3.47187900 | 1.77913600 | -0.85850600 |
| C | -2.32655300 | -3.27589000 | 0.22849000 |
| H | -4.74570300 | -2.32164400 | -0.58085200 |
| H | -5.21236300 | 0.06676400 | -1.10568300 |
| C | -1.04506900 | -3.58364800 | 0.60965900 |
| C | -0.10035800 | -2.54506400 | 0.69782500 |
| H | -3.08637000 | -4.05199200 | 0.13590400 |
| H | -0.74563200 | -4.60428000 | 0.83409400 |
| H | 0.93139700 | -2.75332100 | 0.98510200 |
| N | -0.39963600 | -1.28543900 | 0.44328200 |
| N | -0.89385900 | 1.34558300 | -0.04936600 |
| C | -1.13618500 | 2.63298500 | 0.31386800 |
| C | 1.18640000 | 3.28196500 | -0.59915200 |
| C | 2.01107900 | 2.09537200 | -0.17152000 |
| H | 2.53229100 | 2.29154300 | 0.78227100 |
| O | -2.25310000 | 3.07981200 | 0.62516100 |
| Pd | 0.91798800 | 0.42968400 | 0.20175400 |
| O | 2.77494300 | -0.58737600 | 0.54044100 |
| C | 3.28779900 | -1.30948900 | -0.32011000 |
| O | 2.76634100 | -1.47940800 | -1.51850500 |
| C | 4.53983300 | -2.07057200 | -0.09478300 |
| H | 5.26652600 | -1.82594900 | -0.87821300 |
| H | 4.32652500 | -3.14425000 | -0.16944600 |
| H | 4.95360800 | -1.84212100 | 0.88963900 |

| | | | |
|---|---|---|---|
| H | 1.93754400 | -0.95286300 | -1.59888800 |
| H | 2.75957200 | 1.80483700 | -0.92709200 |
| C | 0.04799800 | 3.57630900 | 0.36284200 |
| H | 0.43152200 | 3.57570400 | 1.39787900 |
| H | -0.36401700 | 4.58018200 | 0.19380000 |
| H | 0.78280300 | 3.12813100 | -1.61269000 |
| H | 1.82696300 | 4.17947900 | -0.65957200 |
